# Atlantic oceanic islands and archipelagos: Physical structures, plant diversity, and affinities of the bryofloras

**DOI:** 10.3897/BDJ.13.e141577

**Published:** 2025-02-28

**Authors:** Denise Pinheiro da Costa, Cecília Sérgio

**Affiliations:** 1 Instituto de Pesquisas Jardin Botanico do Rio de Janeiro, Rio de Janeiro, Brazil Instituto de Pesquisas Jardin Botanico do Rio de Janeiro Rio de Janeiro Brazil; 2 Centre for Ecology, Evolution and Environmental Changes (CEEC), Global Change and Sustainability Institute (CHANGE), Natural History and Systematics (NHS), Research Group, Rua da Escola Politécnica 56/58, 1250–102, Lisboa, Portugal Centre for Ecology, Evolution and Environmental Changes (CEEC), Global Change and Sustainability Institute (CHANGE), Natural History and Systematics (NHS), Research Group, Rua da Escola Politécnica 56/58, 1250–102 Lisboa Portugal

**Keywords:** Atlantic Islands, mosses, liverworts and hornworts, distribution, endemism, similarity

## Abstract

We investigated the species richness, endemism, and similarities of the bryofloras on ten islands and archipelagos in the Atlantic Ocean. We address the following questions: 1) How many bryophyte taxa are there on each island and archipelago? 2) Do mosses and liverworts share similar patterns of diversity? 3) What are their taxonomic distribution patterns? 4) How many endemic taxa are found on the islands? 5) Is there a high degree of similarity amost these bryofloras? We encountered 1,498 taxa, 408 genera, and 204 families, with the seven families representing 35% of all species. Over 14% of the bryophytes are African elements, 8% have global distributions, ca. 3% are Macaronesia elements, 13% are endemic, and 62% with other distributions. We present a checklist of 1,498 bryophyte taxa, of which 201 are endemic (13%). Bryophyte richness and diversity differ significantly amongst the ten islands and archipelagos, and their low similarities illustrate their high degrees of heterogeneity.

## Introduction

Scientists have long been fascinated by the unique nature of remote oceanic islands, especially after Charles Darwin’s study of the Galapagos Islands. Young oceanic islands of volcanic origin represent the summits of large volcanoes that have risen from the ocean floor. They are isolated from other landmasses by deep seas and have never been connected to continental landmasses, making their environments unique products of trans-oceanic dispersal and subsequent speciation (Wace and Holdgate 1976, Cowie and Holland 2006). Islands therefore represent natural laboratories that simplify the complexities of the natural world (Whittaker and Fernández-Palacios 2007). However, the introduction of non-native species poses a constant threat to island biota. It is widely recognized that warmer temperatures can enhance the likelihood of alien invasive species settling, reproducing and successfully outcompeting with the native biota, leading to significant biodiversity losses (Gritti et al. 2006, Burgier and Muir 2010).

**Figure 1.**
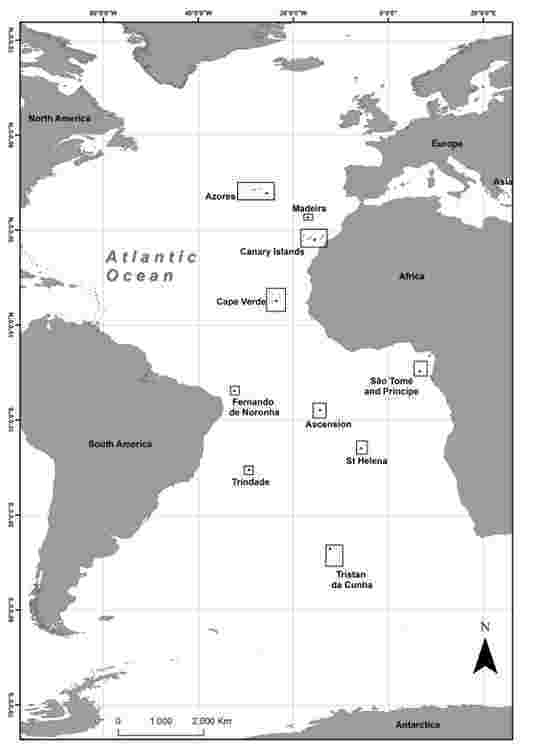
Locations of islands and archipelagos analyzed in the Northern and Southern Atlantic Oceans.

There are numerous islands and archipelagos in the Atlantic Ocean (Hansom 2010): including the archipelagos of the Azores and Madeira within the territorial waters of Portugal; the archipelago of the Canary Islands (Spain); the archipelagos of Cape Verde and São Tomé and Príncipe (Africa); Fernando de Noronha, Trindade and Martin Vaz (Brazil); and the islands of St Helena, Ascension and the archipelago of Tristan da Cunha (England) (Fig. 1).

The bryofloras of the ten islands and archipelagos were inventoried: Azores, Madeira, São Tomé and Príncipe, Cape Verde, and Canary islands (Northern Atlantic) and Trindade, Fernando de Noronha, Ascension, Saint Helena and Tristan da Cunha (Southern Atlantic) to: (1) provide an overview of their bryofloras; (2) describe the species richness of mosses and liverworts and incorporate new reports of bryophyte taxa; (3) analyze the distribution patterns and endemism of those species; (4) analyze the similarities amongst northern and southern islands; and (5) establish a diversity baseline for comparisons. We describe here the diversity, endemism, and similarities of mosses and liverworts on ten islands and archipelagos located in the Atlantic Ocean.

### Studied islands and archipelagos (physical structures and bryofloras)

The **Portuguese archipelagos** are of volcanic origin and represent important geological sites. The **Azores** archipelago comprises nine islands, located approximately 1,600 km from the European coast. It is a chain of submarine volcanic islands with sources of fresh water, and the prevailing climate is temperate (Elias et al. 2016). The archipelago hosts a total of 471 bryophyte taxa, with the diversity among the different islands varying greatly, ranging from 104 to 324 taxa; only 5% of the archipelago is covered by forest vegetation (Tables 1, 3). The **Madeira** archipelago is located approximately 978 km from Portugal and about 700 km from Africa, with climate Mediterranean (Elias et al. 2016). It is also of volcanic origin, with two main islands (Madeira and Porto Santo), as well as two groups of uninhabited islands (Desertas and Selvagens). The highest points there are the Pico Ruivo (1862 m a.s.l.), Pico das Torres (1851 m a.s.l.), and Pico do Arieiro (1818 m a.s.l.). The mountainous landscape and their exposure to constant winds mean that the small islands have different microclimates and higher precipitation rates on the northern coast; few places have original intact vegetation and they have no sources of fresh water. The Madeira archipelago has 586 taxa of bryophytes (Tables 1, 3).

The **African archipelagos** are likewise of volcanic origin and include two islands (**São Tomé and Príncipe**) and some islets. São Tomé is the largest island in the archipelago and consists of two islands formed during the tertiary era. The archipelago is located off the coast of central Africa (ca. 250 km distant), close to the Equator. The island of São Tomé is the capital of the archipelago, with an area of ca. 1,001 km². The climate of São Tomé is equatorial, hot, and humid, with temperatures varying according to altitude and exposure, ranging from 22 to 30°C. The island is steep, with a plain zone, high mountains to the south and west, a flat landscape to the north, and relatively fertile soil. It holds a total of 292 taxa of bryophytes. **Cape Verde** is an island country that is part of an archipelago formed by 10 islands covering ca. 4,033 km², located off the coast of West Africa. A total of 207 taxa of bryophytes have been recorded there, with the islands of Santo Antão, Santiago, and Fogo having the highest numbers of plant taxa. Santo Antão has the highest species richness of bryophytes, with 111 taxa (54% of the archipelago's total bryoflora), (Tables 1, 3).

The **Spanish archipelago** of the **Canary Islands** is of volcanic origin, having emerged 8-12 million years ago and covers an area of 7,747 km^2^. The climate is Mediterranean (Elias et al. 2016). The archipelago forms part of Macaronesia, along with Madeira, the Azores and Cape Verde. A total of 522 bryophyte taxa are recognized in the Canary Islands (Tables 1, 3).

The **Brazilian archipelagos** are also of volcanic origin and represent important national and international geological sites. The **Trindade archipelago** is located approximately 1,100 km from the Brazilian coast and has a tropical climate. Its mountainous islands belong to a chain of submarine volcanoes, with a source of fresh water. Most of the vegetation on the archipelago is herbaceous, with only 5% of the vegetation being forest; it harbours 35 species of bryophytes, without endemic species. The **Fernando de Noronha archipelago** consists of 21 small volcanic islands located approximately 350 km from the Brazilian coast, with tropical climate. There are few areas of original vegetation, and the islands lack a source of fresh water. Most of the vegetation is dry forest similar to Caatinga, dominated by small trees, shrubs, and grasses. This archipelago supports 29 bryophyte taxa, including one endemic species (Tables 1, 3).

The two islands and the English archippelago are also of volcanic origin. Ascension is a small island covering only 91 km², located 1,660 km from Africa and 2,250 km from South America; it is approximately 1 million years old and has a dry tropical oceanic climate with little seasonal variation; its highest peak is Green Mountain (859 m a.s.l.). The island has three vegetation zones related to altitude: a bare and rocky dry zone at 0-330 m a.s.l.; the base surroundings of the mountain are at altitudes between 330-660 m, with few bryophytes species; and the top of Green Mountain, altitude > 660 m, with a cloud forest and vegetation dominated by ferns and bryophytes (84 taxa of bryophytes, with 15 endemic), (Tables 1, 3). The Island of Saint Helena is a volcanic island covering 122 km^2^, situated 4,000 km east of Rio de Janeiro (Brazil) and 1,950 km west of Africa; it is approximately 7 million years old, has a rugged terrain and is one of the most remote regions in the world. The climate is tropical, marine, and mild, with continuous winds, with an average annual temperature of 17-28°C. The highest point is Pico Diana (818 m a.s.l.), whose high-altitude areas concentrate endemic species. It has three vegetation zones (tree fern forests; dry and eroded pastures and coastal zones; medium elevations with highly altered vegetation), and a total of 101 bryophyte taxa, with 27 endemic (Tables 1, 3). Tristan da Cunha is an archipelago located in the temperate South Atlantic, of volcanic origin and formed by three main islands: Gough (cool-temperate climate), Inaccessible (cool-temperate climate) and Nightingale. The archipelago hosts a total of 303 bryophyte taxa, including 51 endemic species, (Tables 1, 3).

### Methods

**Data collection**. A dataset was compiled in an Excel spreadsheet containing information on the taxa growing on the archipelagos of the Azores, Madeira, São Tomé and Príncipe, Cape Verde and the Canary Islands, based on the literature (Suppl. materials 1, 2 – Supplemental Data). The plant names were updated to include information concerning their distributions, endemism and conservation status, resulting in a total of 1,498 recorded taxa. The dataset also included information on the bryophyte taxa found on the islands and archipelagos of Trindade, Fernando de Noronha, Ascension, Saint Helena and Tristan da Cunha, with a final total of 1,498 taxa (624 hornworts and liverworts and 874 mosses). Due to the very low number of hornwort species, these were grouped with liverworts.

***Herbarium collections***. Subsequently, approximately 600 specimens from the archipelago of São Tomé and Príncipe, around 80 from the archipelago of Cape Verde, and ca. 330 samples from the Azores archipelago were verified at the LISU herbarium. The nomenclature and synonyms for each species were verified using databases such as TROPICOS (Missouri Botanical Garden (2024)) and some new literature. Nomenclature was standardized following Hodgetts and Lockhart (2020) for mosses and liverworts and Mouton et al. (2023).

**Data analysis (diversity and floristic affinities)**. To determine bryophyte diversity on the ten islands and archipelagos, a floristic matrix was prepared using incidence data (presence/absence) to quantify diversity (Suppl. materials 1, 2 – Supplemental Data). Rare species (those occurring on only a single island or archipelago) were included. We used this matrix to calculate the Jaccard index, assess floristic similarities, and build a dendrogram of plant diversity amongstthe ten islands and archipelagos. These statistical analyses were performed using PAST programme version 4.12 (Hammer et al. 2001). The phytogeographic patterns of the taxa were characterized, based on their global distributions, according to information available in the scientific literature and databases (O’Shea 2006, Patiño–Llorente and Gonzales–Mancebo 2005, Wigginton 2004, Wigginton 2018, Missouri Botanical Garden 2024, and the general literature). These datasets included the more important literature and were used to analyze differences in the bryofloras of the ten islands and archipelagos in the Atlantic Ocean in relation to their total numbers of taxa, endemic taxa, exotic taxa, and possibly extinct taxa (Table 3), as well as the predominant families and any exclusive ones (Table 2).

## Results and Discussion

**Diversity of the ten islands and archipelagos of the Atlantic Ocean (alpha, beta and gamma diversity).** A total of 1,498 taxa (gamma diversity) were listed for the ten islands and archipelagos in the Northern and Southern Atlantic Ocean, including 874 taxa of mosses and 624 taxa of liverworts and hornworts. Of these, 201 taxa are endemic (73 taxa of liverworts and hornworts and 128 taxa of mosses), representing 13% of all taxa surveyed (Suppl. material 2); only 10 taxa were shared by all five archipelagos. The number of endemic taxa in the five North Atlantic Ocean archipelagos (107 spp.) was higher than the five islands and archipelagos of the South Atlantic Ocean (91 spp.), highlighting their importance to the conservation of bryophyte biodiversity on Atlantic Ocean islands. This is especially critical given that these islands are likely to experience the consequences of climate change, compounded by ongoing pressures such as tourism, pollution, population growth, the introduction of alien species and habitat destruction (Fernández-Palacios et al. 2021). Bryophyte richness and diversity differed significantly amongst study sites illustrating their high degrees of heterogeneity and reflected the importance of habitat differences for high diversity.

with the inclusion of taxa from the South Atlantic Ocean islands and archipelagos (Fernando de Noronha, Trindade, Saint Helena, Ascension, and Tristan da Cunha) for analysis and comparison with those of the North Atlantic Ocean (**gamma diversity**), the total number of taxa increased to 1,498 taxa (24 hornworts, 600 liverworts, and 874 mosses).

**Figure 2.**
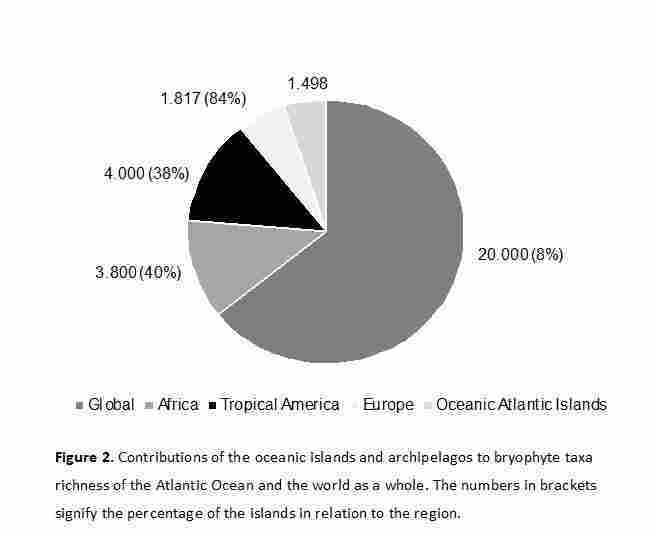
Contributions of oceanic islands and archipelagos to the bryophyte taxa richness of the Atlantic Ocean and the world as a whole. The numbers in brackets indicate the percentage of islands relative to the region.

Species richness varied strongly amongst the ten southern Atlantic islands and archipelagos, ranging from 29 taxa on Fernando de Noronha, to 586 on Madeira (Table 3). The bryophyte diversity on the ten islands and archipelagos studied here (gamma diversity) was high when compared to Africa, Tropical America, and the world (Wigginton 2018, O’Shea 2006, Shaw and Goffinet 2000, Gradstein et al. 2001), (Fig. 2).

**Phytogeographical patterns and endemism.** The islands and archipelagos of North and South Atlantic Oceans were analyzed separately and together with the results presented in Suppl. materials 3, 4. The phytogeographical patterns of the **North Atlantic Ocean** islands and archipelagos were categorized into ten groups: 1) Global (107 taxa; 21 liverworts and 86 mosses); 2) Africa (182 taxa; 99 liverworts and 83 mosses); 3) Endemic (107 taxa; 38 liverworts and 69 mosses); 4) Europe (40 taxa of mosses); 5) North America Europe, Asia (46 taxa; 26 liverworts and 20 mosses); 6) Europe, Macaronesia, Africa (8 taxa; 7 liverworts and 1 moss); 7) Europe, Macaronesia, Asia (16 taxa; 8 liverworts and 8 mosses); 8) Europe and Macaronesia (14 taxa; 5 liverworts and 9 mosses); 9) South America and Africa (5 taxa of liverworts); 10) others (597 taxa; 215 liverworts and 382 mosses). The African, Endemic and Global elements were the largest, comprising 35% of the total taxa (Suppl. material 3).

Distinct phytogeographical patterns were observed for liverworts and mosses on the North Atlantic Ocean islands (Suppl. material 3). Liverworts exhibited low numbers of European and Macaronesia taxa (5 taxa), but the highest numbers of African elements (99), followed by endemic taxa (38) and global elements (21). In contrast, mosses exhibited a high number of global elements (86), followed by African and endemic elements (83 and 69, respectively). The phytogeographical patterns of the South Atlantic Ocean islands and archipelagos were classified into seven categories for this analysis: 1) Global (50 taxa; 15 liverworts and 35 mosses); 2) Pantropical (20 taxa; 10 liverworts and 10 mosses); 3) Tropical America (21 taxa; 11 liverworts and 10 mosses); 4) Africa (19 taxa; 6 liverworts and 13 mosses); 5) South America (91 taxa; 75 liverworts and 16 mosses); 6) Endemic (91 taxa; 36 liverworts and 55 mosses); 7) others (171 taxa; 86 liverworts and 85 mosses). Endemic elements represented the largest category, comprising approximately 37% of all taxa (Suppl. material 4).

Liverworts and mosses exhibited distinct phytogeographical patterns on the South Atlantic Ocean islands and archipelagos (Suppl. material 4). Liverworts had a high number of taxa with South American distributions (75 taxa), followed by endemic taxa (36), but low number of taxa with global distributions (15); mosses had high number of endemic taxa (55) and taxa with global distributions (35).

**Figure 3.**
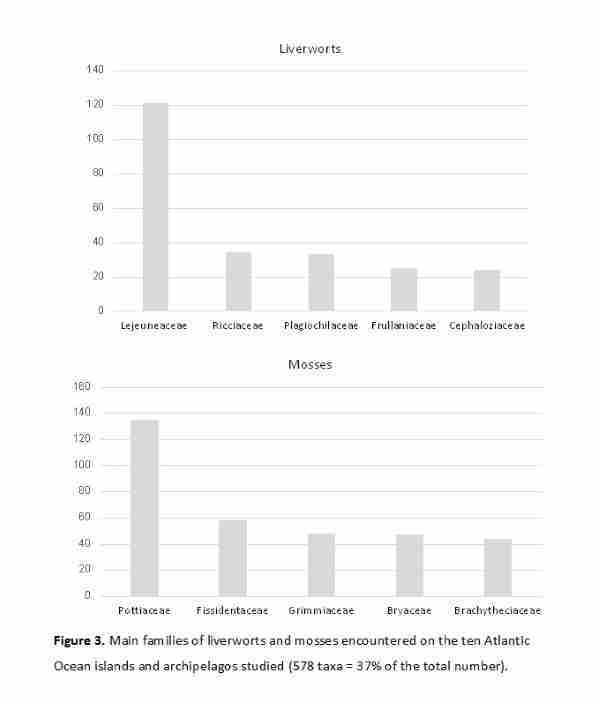
Principal families of liverworts and mosses identified on the ten Atlantic Ocean islands and archipelagos studied (578 taxa, representing 38% of the total).

A notable feature of the bryofloras of the **North Atlantic Ocean** archipelagos is that species richness was concentrated in a few families (Table 2, Fig. 3). Amongst mosses, the families Brachytheciaceae (44 taxa), Bryaceae (47), Fissidentaceae (58), and Pottiaceae (135) stood out, collectively accounting for 284 taxa – fully 31% of the total of moss taxa (901) recorded for the five archipelagos. For liverworts, just three families (Lejeuneaceae [121 taxa], Plagiochilaceae [33], and Ricciaceae [34]) represented 30% of the total liverwort species (629) recorded for these archipelagos. Thus, the richness of bryophyte taxa was concentrated in seven families that collectively accounted for 40% of the total taxa (1,161).

The taxa richness of the bryofloras of the South Atlantic Ocean islands and archipelagos was concentrated in six families (Table 2, Fig. 3), Lejeuneaceae (38 taxa), Lophocoleaceae (40), Bryaceae (25), Fissidentaceae (28), Grimmiaceae (11), and Pottiaceae (25), which together totalled 167 taxa, representing 27% of the total.

There were differences amongst the islands and archipelagos with unique families, or amongst those which had significantly different numbers of taxa (**beta diversity**) (Tables 1, 3). The Brazilian archipelagos in the South Atlantic Ocean (Trindade and Fernando de Noronha) illustrated how habit destruction by humans can influence bryophyte species diversity. These archipelagos had only 35 and 29 taxa, respectively, the lowest diversity of bryophyte taxa amongst the ten islands and archipelagos studied and neither contained any endemic taxon. This situation contrasts with the two English islands and one archipelago (Ascension, Saint Helena, and Tristan da Cunha), which have between 84 and 303 bryophytes taxa, including 15 to 51 endemic taxa – demonstrating their higher degrees of preservation. The African archipelagos of Cape Verde and São Tomé and Príncipe in the North Atlantic Ocean have between 207 and 292 bryophyte taxa, including 8 to 19 endemic taxa. The Portuguese archipelagos of Azores and Madeira and the Spanish Canary Islands archipelago evidenced the highest diversity and shared more taxa, with the Canary Islands being the most preserved (with a total of 522 bryophyte taxa, 47 of which are endemic).

**AZORES.** Three bryophyte families occur exclusively in this archipelago. The family Jamesoniellaceae is represented here by a species that is otherwise restricted to the Neotropics: *Syzygiellarubricaulis* (Nees) Steph. The family Sphagnaceae is also notable in this archipelago, with 17 taxa (Corley et al. 1981, Grolle 1983, Sjögren 2001, Gabriel et al. 2010, Hodgetts and Lockhart 2020). The Azores share the highest number of taxa with the **Madeira** archipelago (372 taxa, including 37 endemic species).

**MADEIRA.** Three families occur exclusively on this archipelago: Blasiaceae (Liverworts), Aongstroemiaceae, and Flexitrichaceae (Mosses). Additionally, the families Brachytheciaceae (34 species) and Grimmiaceae (27) are particularly prominent (Fontinha et al. 2020, Hodgetts and Lockhart 2020, Sérgio et al. 2006, Sérgio et al. 2008). As noted earlier, Madeira shares the highest number of taxa with the Azores.

**SÃO TOMÉ and PRÍNCIPE.** This archipelago has the highest number of restricted families. It is the only archipelago where the hornwort family Dendrocerotaceae is present, alongside moisture-indicating families such as Hypopterygiaceae and Meteoriaceae. São Tomé and Príncipe share 26 taxa with Cape Verde, 24 with the Canary Islands, 24 with the Azores and 26 with the Madeira.

**CAPE VERDE.** Two bryophyte families occur exclusively in this archipelago. A total of 69 exclusive taxa are found here, with seven being endemic. Cape Verde is the most unique archipelago amongst the five studied in the North Atlantic Ocean, with the smallest total number of taxa and endemic taxa. However, it shares a significant number of taxa with the Canary Islands (125 taxa).

**CANARY ISLANDS.** Three bryophyte families occur exclusively in this archipelago. The Canary Islands share the most taxa with the Azores and Madeira (298 taxa, 37 of which are endemic) and with Cape Verde (125 taxa). Together with Madeira, the Canary Islands have the best-documented bryofloras, which have been extensively detailed in numerous publications (Table 3).

**TRINDADE.** No bryophyte family occurs exclusively on this island. Its native vegetation has been almost completely destroyed through the grazing of introduced animals. E. Knight visited the island in 1881 and 1889 and reported its already degraded vegetation and a large waterfall on the west coast. Vegetation restoration efforts were initiated in 1990 through the elimination of goats (Silva and Alves 2011). Since then, the vegetation has slowly recovered, and the creeks now have more water. The vegetation above 400 m is considered a “Giant Fern Forest" and is dominated by a single species, *Cyatheadelgadii* Sternb.; it is the most diverse region for bryophytes. Most of the taxa are Neotropical, followed by cosmopolitan taxa; there are no endemic taxa (Costa and Rezende 2022). The growth of *Campylopusintroflexus* (Hedw.) Brid. was probably facilitated by human impacts on the original vegetation (Gama et al. 2016). This archipelago shares four taxa with Ascension Island and four taxa with the Fernando de Noronha archipelago.

**FERNANDO DE NORONHA.** Only one moss family, Stereophyllaceae, occurs exclusively on this island. Charles Darwin visited the island and made the first botanical collections there. A later expedition by Ridley (1890) reported the predominance of herbaceous vegetation due to the intensive cutting of tall trees that could be used to build rafts and aid prisoner escapes. *Fissidens*, with seven taxa, is the richest genus. Cosmopolitan taxa compose approximately 26% of vegetation; there are only one endemic taxa, *Fissidensnoronhensis* Teixeria, Bordin and Carv.-Silv. This archipelago shares seven taxa with Ascension Island (Costa and Rezende 2022, Costa et al. 2021, Pressel et al. 2017, Yano and Mello 2016, Pereira and Câmara 2015).

**ASCENSION.** No bryophyte family occurs exclusively on this island. The island harbours over 200 introduced taxa ranging from large trees to shrubs and herbs; many native plants, such as the endemic fern *Pitsanapurpurascens* (de Vriese) Murdock, are becoming severely outcompeted. *Campylopus*, with seven taxa, is the richest genus. There is a notable absence of cosmopolitan taxa (*Ceratodonpurpureus* (Hedw.) Brid., *Tortulamuralis* Hedw., *Octoblepharumalbidum* Hedw., etc.), (Pressel et al. 2017). There are no representatives of Grimmiales, despite the highly suitable volcanic rock substrate. The island has ten endemic bryophyte taxa. A handful of taxa are probable human introductions, but, despite the small size of the island and the destruction of its natural habitats, relatively few bryophytes are threatened with extinction. This island shares 24 taxa with Saint Helena Island, three of which are endemic (Pressel et al. 2017, Wigginton 2012).

**SAINT HELENA.** No bryophyte family occurs exclusively on this island, but there is a notable absence of cosmopolitan taxa, *Ceratodonpurpureus*, *Tortulamuralis*, *Octoblepharumalbidum*, etc. There are no representatives of Grimmiales despite the highly suitable volcanic rock substrate there. *Fissidens*, with 11 taxa, is the richest genus. The destruction of the natural vegetation began with the introduction of goats and the establishment of permanent settlements there introduced exotic vegetation. Native trees were cut for different purposes (house building, fuel etc.) and many endemic taxa are now extinct or critically endangered (Wigginton 2012). This archipelago shares 17 taxa with the Tristan da Cunha archipelago, two of which are endemic (Table 3).

**TRISTAN DA CUNHA.** Eleven bryophyte families occur only on this archipelago (Table 2). There are currently very few introduced taxa on the archipelago. The family Orthotrichaceae is represented by several endemic taxa, and *Dicranoloma* (Renauld) Renauld by three endemic taxa (Váňa and Engel 2013, Wace and ​​​​​​​Dickson 1965, Wace 1961, Dixon 1960). This archipelago shares 35 taxa with the **Azores** archipelago and 32 with **Madeira**, none are endemic. There are strong indications of a close connection with South America, as mosses, liverworts and hornworts can be dispersed by the western winds – ‘West Wind Drift’ – and connections with South America are conspicuous in all groups of non-vascular plants, except the algae (Muñoz et al. 2004, Galloway 1996).

Sunding (1979) considered Cape Verde to be different from the other archipelagos of Macaronesia, which was subsequently corroborated by Vanderpoorten et al. (2007). According to these authors, Macaronesia (comprising the archipelagos of Azores, Madeira, Canary Islands and Cape Verde) is considered a single biogeographical unit, with their floras being relics of a widely distributed Tertiary subtropical flora. These authors rejected the concept of Macaronesia *sensu lato* and viewed the Cape Verde archipelago as being more associated with tropical Africa. Liverworts in the other Macaronesian archipelagos support an Azores-Madeira-Canary clade (Macaronesia *sensu stricto*), while mosses support the Canary Islands as being related to North Africa, rejecting the concept of Macaronesia *s.s.* for the group. The exchange of taxa with neighbouring continental areas better explains the relationships between the cryptogamic flora of Cape Verde and the mossy flora of the Canary Islands. In contrast, the relic flora is consistent with a monophyletic Macaronesia *s.s.* group of liverworts. These congruent patterns, however, can hide a complex mix of relict distributions and more recent speciation and dispersal events. According to Mouton et al. (2023), the differences in beta diversity patterns, caused by different responses of Macaronesian land plant lineages to the main factors shaping their community composition, explain their different biogeographic affinities.

**Figure 4.**
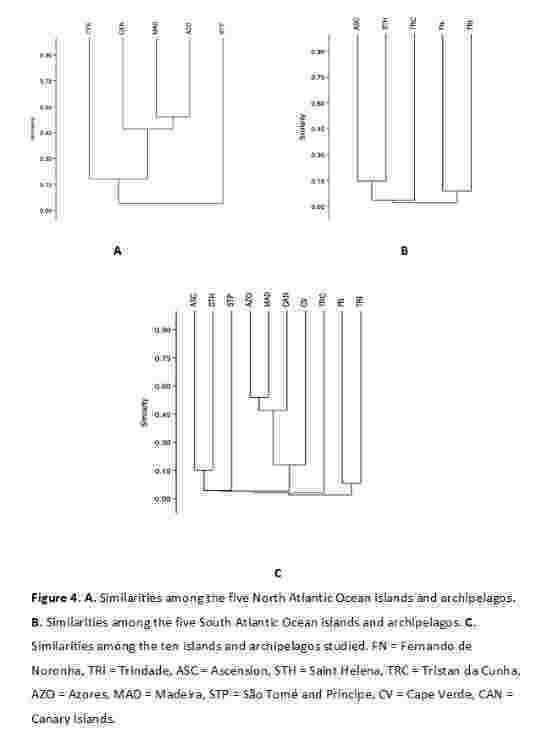
**A** Similarities amongst the five North Atlantic Ocean islands and archipelagos; **B** Similarities amongst the five South Atlantic Ocean islands and archipelagos; **C** Similarities amongst the ten islands and archipelagos studied. FN = Fernando de Noronha, TRI = Trindade, ASC = Ascension, STH = Saint Helena, TRC = Tristan da Cunha, AZO = Azores, MAD = Madeira, STP = São Tomé and Príncipe, CV = Cape Verde, CAN = Canary Islands.

**Similarities amongst the ten islands and archipelagos** (beta diversity). The results of our study, when comparing the bryofloras of the ten Atlantic Ocean islands and archipelagos, demonstrated that their bryofloras were very dissimilar, with low floristic affinities (Fig. 4). Amongst the ten islands and archipelagos, the ones demonstrating the greatest similarity were the archipelagos of Macaronesia – Azores, Madeira and the Canary Islands – which share 278 taxa (Fig. 4A–C). Our study also suggests, based on the compilation of bryophytes, Macaronesia as being formed by the Azores, Madeira, and the Canary Islands archipelagos, with Cape Verde outside the clade when the islands and archipelagos of the **North Atlantic Ocean** were analyzed separately or together with the islands and archipelagos of the South Atlantic Ocean (Fig. 4A, C). As expected, the Azores, Madeira, and Canary Islands archipelagos (Macaronesia *s.s.*) share several taxa and evidenced the highest similarity. The archipelago of São Tomé and Príncipe was found to be highly dissimilar to the others, sharing few taxa with any of the North Atlantic Ocean archipelagos (Fig. 4A, C).

The results for the **South Atlantic Ocean** also show low similarity amongst most islands and archipelagos. However, Saint Helena and Ascension demonstrated the highest similarity and formed a distinct clade (with Tristan da Cunha outside of it). Saint Helena and Ascension share several taxa. The archipelagos of Trindade and Fernando de Noronha also formed a separate clade, despite sharing few taxa; this can be attributed to the several ecological degradations suffered by these two archipelagos since their discovery and occupation (Fig. 4B, C).

When we analyzed all ten Atlantic Ocean islands and archipelagos together, their similarities were very low, except among Madeira, the Azores, and the Canary Islands (Marcaronesia *s.s.*), each of which demonstrates a largely unique bryoflora. Our results also corroborate the exclusion of Cape Verde from Macaronesia (Fig. 4C).

**Conservation.** The ten islands and archipelagos studied here have proven to be rich areas for bryophytes (with the exception of Fernando de Noronha and Trindade). Their bryophyte diversity represents approximately 40% of all taxa known from Africa (3,800 species), 38% of all bryophyte taxa known from tropical America (4,000 species) and 8% of all species globally (Wigginton 2018, O’Shea 2003, O’Shea 2006, Gradstein et al. 2001). Our study indicated that the vegetation on the islands and archipelagos must be preserved and protected, as 200 taxa are only known from them. However, when analyzed separately, the Brazilian islands are not particularly rich in bryophyte diversity – this observation holds true only when considering all ten islands together. Additionally, more botanical collections should be undertaken on Cape Verde, Tristan da Cunha and São Tomé and Príncipe. Our results emphasize the importance of the bryofloras of the Atlantic Ocean islands and archipelagos for bryophyte conservation, as they contain high percentages of the total bryophyte diversity of Africa and tropical America, as well as the taxa diversity known for the Macaronesia region, despite their small areas.

The main threats to biodiversity on all ten islands and archipelagos include species introductions, pollution (from maritime traffic, tourism and fishing), the introduction of invasive species with their potential to alter native habitats and compete with native species (as observed on Saint Helena, where New Zealand flax (*Phromiumtenax* J.R. Forst. & G. Forst., a monocotyledon from the Asphodelaceae family) and the moss *Pseudoscleropodiumpurum* (Hedw.) M. Fleisch., from the Brachytheciaceae family, pose significant threats) and rising ocean levels due to climate change (Thomas et al. 2020, Soares 2018). According to Wigginton (2012), *Pseudoscleropodiumpurum* has been used as packing material for imported plants and should be treated as an introduced species in Saint Helena.

Five of the North Atlantic Ocean islands and archipelagos studied here had 240 taxa classified as being threatened to some degree (2 EX – extinct [According to Sim-Sim et al. (2019a), Sim-Sim et al. (2019b), both from Madeira island, *Fissidensmicrostictus* Dixon & Luisier and *Nobregaealatinervis* Hedenäs]; 15 CR – critically endangered; 72 EN – endangered; 42–145 VU – vulnerable; 6 LC – least concern) – fully 20% of the total number of taxa (Hodgetts 2019). This study confirms the significance of these islands and archipelagos as key sites of bryophyte diversity for Macaronesia and Africa. However, there is a lack of adequate protection for endemic and/or endangered taxa on these islands and archipelagos. Addressing this should be a priority, as global warming is expected to accelerate the destruction of these sensitive environments worldwide.

The bryophytes of the Macaronesian laurel forests (*Laurisilva*) are also at considerable risk due to climate change (Patiño–Llorente et al. 2016, Patiño–Llorente and Vanderpoorten 2018, Carvalho et al. 2022). Many areas are predicted to become water-deficient and forest fires are becoming more frequent – with projections indicating significantly increased risks to important endemic taxa (Hodgetts 2019).

Many other bryophyte taxa are under conservation threat, with tropical species often known solely from type specimens or small private collections. For example, approximately 45 bryophyte taxa reported for São Tomé and Príncipe have not been collected again since the 19^th^ century (Costa et al. 2024, Garcia et al. 2012, Sérgio and Garcia 2011). The ecology, habitat specificity, and distribution patterns of many bryophyte taxa remain poorly understood, while threats to forest habitats in São Tomé and Príncipe continue to escalate (including habitat destruction and competition from invasive taxa (for example Figueiredo et al. 2011) can affect the survival of many bryophyte taxa (Garcia et al. 2022).

## Conclusions and conservation implications

This study represents the first comprehensive survey of bryophyte diversity across ten Atlantic Ocean islands and archipelagos. The findings have significantly enhanced our understanding of the bryofloras in island habitats, introducing numerous novelties and new records. These data are crucial for the conservation of bryophyte diversity on Atlantic Ocean islands and archipelagos and can contribute to achieving the targets of the Global Strategy for Plant Conservation (GSPC). The survey provides a deeper understanding of the floristic compositions of these islands and archipelagos, increasing our estimates of taxa richness and distributions. However, this information remains incomplete, highlighting the need for additional inventories in unexplored regions of the Cape Verde, Tristan da Cunha, and São Tomé and Príncipe archipelagos (see references and Table 3). Bryophyte richness and diversity differed significantly amongst study sites (alpha diversity), and their low similarities (beta diversity) illustrated their high degrees of heterogeneity and reflected the importance of habitat differences for high diversity. Understanding the factors influencing taxa richness and endemism on oceanic islands is vital for effective conservation efforts and biodiversity management, particularly in the context of global climate change (Grabherr et al. 2010). Climate change is expected to result in strong impacts on oceanic islands and archipelagos, with habitat losses and declines and extinction risks, as isolated bryophyte taxa in these regions have no access to refuges. The complexity of island ecosystems, characterized by high diversity and endemism, requires researchers to generate primary data and integrate geological and climatic information into diversity models. Further studies are essential for developing proactive conservation plans, for these environments harbour a significant portion of the world’s biodiversity. The main threats to taxa diversity, forest structure, and habitats include urbanization, habitat destruction and fragmentation, the introduction of invasive taxa that can affect the survival of native taxa, uncontrolled tourism and rising oceans (which will mainly affect islands smaller than 1,000 ha - Médail (2017)). Our findings establish baselines for bryophyte diversity and endemism across ten Atlantic Ocean islands and archipelagos, providing benchmarks for future surveys. These data can help determine whether taxa can adapt or face extinction in response to climate change. The unique characteristics of those oceanic islands and archipelagos necessitate conservation strategies and address their biological and environmental diversities while recognizing their importance as protected areas.

## Supplementary Material

5B6D0CC4-D026-563B-AE22-900CE5019FB8 10.3897/BDJ.13.e141577.suppl1 Supplementary material 1 Table S1. Matrix of presence and absence of liverworts and hornworts taxa Data type Table Brief description Matrix of presence and absence of liverworts and hornworts taxa on the ten islands studied. *= Endemic File: oo_1163475.docx https://binary.pensoft.net/file/1163475 DP Costa & C Sérgio

E3D44B25-C81D-544E-8906-420F0591E48B 10.3897/BDJ.13.e141577.suppl2 Supplementary material 2 Table S2. Matrix of presence and absence of moss taxa Data type Table Brief description Matrix of presence and absence of moss taxa on the ten islands studied. *= Endemic File: oo_1163476.docx https://binary.pensoft.net/file/1163476 DP Costa & C Sérgio

703CC245-057A-52DA-9E1A-027C0B560371 10.3897/BDJ.13.e141577.suppl3 Supplementary material 3 Table S3. Phytogeographical patterns of the bryofloras in the North Atlantic Ocean Data type Table Brief description Phytogeographical patterns of the bryofloras of the five North Atlantic Ocean islands and archipelagos. File: oo_1163479.docx https://binary.pensoft.net/file/1163479 DP Costa & C Sérgio

81D5E763-9EAC-5B0D-AB2A-3016C980991B 10.3897/BDJ.13.e141577.suppl4 Supplementary material 4 Table S4. Phytogeographical patterns of the bryofloras in the South Atlantic Ocean Data type Table Brief description Phytogeographical patterns of the bryofloras of the South Atlantic Ocean islands and archipelagos (Trindade, Fernando de Noronha, Ascension, Saint Helena, and Tristan da Cunha). File: oo_1163480.docx https://binary.pensoft.net/file/1163480 DP Costa & C Sérgio

## Figures and Tables

**Table 1.** General data on the ten islands and archipelagos studied. AZO = Azores, MAD = Madeira, STP = São Tomé and Príncipe, CV = Cape Verde, CAN = Canary Islands, FN = Fernando de Noronha, TRI = Trindade, ASC = Ascension, STH = Saint Helena, TRC = Tristan da Cunha, END = Endemic.

**AREA**	**ORIGIN**	**CLIMATE**	**MAIN HABITAT**	**MOSSES**	**LIVERWORT**	**HORNWORT**	**TOTAL**
**AZO**	Volcanic (9 islands), 0.3-0.8 mya, 2,351 Km^2^, population: 236,440 permanent residents, highest point is Pico (2,351 m a.s.l.)	Subtropical oceanic, 17°C, 700-900 mm precipitation	Laurissilva forest (humid forest composed mainly of tress of the Lauraceae family and endemic to Macaronesia). It is found in isolated patches on all the islands, the largest being on Pico.	300 spp.	165 spp.	6 spp.	471 spp. (45 END – 10%)
**MAD**	Volcanic (4 islands), 5 mya, 724 724 Km^2^, population: 262,456 permanent residents (Madeira Island), highest peak is Ruivo (1,862 m a.s.l.)	Mediterranean and Temperate in the highest parts, 20°C, 3,400 mm precipitation	Laurissilva forest (best preserved evergreen forests in Macaronesia), with a great diversity of bryophytes covering the trunks and branches of trees and shrubs. Occupies ca. 15,000 hectares (20% of the island).	397 spp.	183 spp.	6 spp.	586 spp. (56 END – 10%)
**STP**	Volcanic (3 islands), 31 mya, 991 Km^2^, population: 213,000 permanent residents, highest peak is São Tomé (2,024 m a.s.l.)	Equatorial oceanic (hot and wet), 22-30°C, 3,200 mm precipitation	Tropical moist broadleaf forest	118 spp.	167 spp.	7 spp.	292 spp. (19 END – 6%)
**CV**	Volcanic (10 islands, and one active vulcan in the Fogo Island), 8-20 mya, 4,033 Km^2^, population: 561,901 (nine islands have permanent residents), highest peak is Fogo (2,829 m a.s.l.)	Arid to semi-arid, 22-27°C, 214 mm precipitation	Savannah or steppe, and tropical climate depending on elevation. Laurissilva forest appears in small patches only on the islands furthest from the African coast and at higher elevations	150 spp.	54 spp.	2 spp.	207 spp. (8 END – 3%)
**CAN**	Volcanic (7 islands), 8-20 mya, 7,493 Km^2^, population: 2,2 million permanent residents, highest peak is Teide (3,715 m a.s.l.)	Warm subtropical and semi-desertic, 16-27°C, 1,321 mm	Subtropical, with xerophytic, humid forest, the laurel forest, fayal-brezal, the pine forest and high mountain vegetation. The Laurissilva forest patches are found on the islands of Gomera, La Palma, Tenerife	350 spp.	166 spp.	6 spp.	522 spp. (47 END – 10%)
**TRI**	Volcanic (5 islands), 3 mya, 10.4 Km^2^, population: 32 soldiers + 8 research (no permanent residents), highest peak is Desejado (620 m a.s.l.)	Tropical oceanic, 25°C, 923 mm precipitation	Exotic plants (100-150 m), lowland (up to 400 m) grass fields, Giant Fern Forest or Giant Ferns Nebular Forest (> 400 m). Less than 5% of the island is covered by forest and ca. 60% by herbaceous vegetation	15 spp.	19 spp.	1 sp.	35 spp. (0%)
**FN**	Volcanic (21 islands and islets), 30 mya, 26 Km^2^, population: 3,100 permanent residents, highest peak is Desejado (323 m a.s.l.)	Tropical wet and dry, 26.5°C, 1,350 mm precipitation	Seasonal deciduous forest, with subxerophytic species (typical of the north-eastern agreste region). Dry Forest (Ponta da Sapata), covering 25% of the shrubs and trees on the main island. Mangrove (the only occurrence of an insular mangrove in the South Atlantic Ocean) is in the Sueste Bay. Creepers covering native bushes and trees during the rainy season.	20 spp.	7 spp.	2 spp.	29 spp. (1 END – 3%)
**ASC**	Volcanic, 1 mya, 91 Km^2^, population: 800 inhabitants (no permanent residents), highest peak is Green (859 m a.s.l.)	Dry tropical oceanic climate, 20-31°C, 130-680 mm precipitation	Dryland (0-300 m), mid-altitude area on Green Mountain (330-600 m), high-altitude with cloud forest area top of Green Mountain (> 660 m)	56 spp.	24 spp.	4 spp.	84 spp. (15 END – 18%)
**STH**	Volcanic (one island), 7 mya, 122 Km^2^, population: 4,500 permanent residents, highest peak is Diana (818 m a.s.l.)	Tropical, 17-28°C, 750-1,000 mm precipitation	Tree fern forest, pastures and coastal zones, dry and eroded, and middle elevations now destroyed	58 spp.	41 spp.	2 spp.	101 spp. (27 END – 26 %)
**TRC**	Volcanic (6 small islands -Nightingale – >18 mya, Inaccessible – 3-4 mya, Tristan da Cunha – 200,000 mya; Gough – 3-5 mya), 207 Km^2^, population: 264 permanent residents (only in Tristan da Cunha), highest peak is Queen Mary (2,062 m a.s.l.)	Cool temperate, 16-25°C, 1670 mm precipitation	Tussock grassland, pastures, fern bush, wet heath, feldmark and alpine, bogs and other wetland, and lava field	142 spp.	161 spp.	4 spp.	303 spp. (51 END – 16%)

**Table 2.** Total number of bryophyte families and genera, as well as the main families in each archipelago or island. **Bold** = families standing out in terms of their numbers of taxa.

**Islands or Archipelagos**	**Families**	**Genera**	**Main families**
Azores	90	128	LIVERWORTS: Aneuraceae, Calypogeiaceae, Cephaloziaceae, **Cephaloziellaceae**, Fossombroniaceae, Geocalycaceae, **Lejeuneaceae**, Lepidoziaceae, **Lophocoleaceae**, Plagiochilaceae, Radulaceae, **Ricciaceae**, Scapaniaceae. MOSSES: Bartramiaceae, **Brachytheciaceae**, Bryaceae, Ditrichaceae, **Fissidentaceae, Grimmiaceae**, Hypnaceae, **Leucobryaceae**, Neckeraceae, Polytrichaceae, **Pottiaceae**, **Sphagnaceae**
Madeira	92	197	HORNWORTS: Anthocerotaceae LIVERWORTS: **Cephaloziaceae**, Cephaloziellaceae, Fossombroniaceae, Frullaniaceae, **Lejeuneaceae**, Marchantiaceae, **Plagiochilaceae**, **Ricciaceae**, Scapaniaceae MOSSES: Amblystegiaceae, Bartramiaceae, **Brachytheciaceae**, **Bryaceae**, Ditrichaceae, **Fissidentaceae**, Funariaceae, **Grimmiaceae**, Leucobryaceae, Mniaceae, Neckeraceae, **Orthotrichaceae**, Polytrichaceae, **Pottiaceae**, Sphagnaceae
São Tomé and Príncipe	62	104	HORNWORTS: Dendrocerotaceae LIVERWORTS: **Frullaniaceae, Lejeuneaceae**, Metzgeriaceae, **Plagiochilaceae**, Radulaceae, Ricciaceae MOSSES: **Bryaceae, Calymperaceae, Fissidentaceae**, Hypnaceae, Orthostichellaceae, Pilotrichaceae, Pottiaceae
Cape Verde	49	105	LIVERWORTS: Frullaniaceae, **Lejeuneaceae, Ricciaceae** MOSSES: Bartramiaceae, **Brachytheciaceae, Bryaceae, Fissidentaceae**, Orthotrichaceae, **Pottiaceae**
Canary	90	187	HORNWORTS: Anthocerotaceae LIVERWORTS: Cephaloziellaceae, **Frullaniaceae, Lejeuneaceae**, Lophocoleaceae, Plagiochilaceae, Radulaceae, **Ricciaceae**, Riellaceae, Sphaerocarpaceae MOSSES: Bartramiaceae, **Brachytheciaceae, Bryaceae**, Ditrichaceae, **Fissidentaceae**, Funariaceae, **Grimmiaceae**, Leucobryaceae, Neckeraceae, Orthotrichaceae, **Polytrichaceae**, **Pottiaceae**,
Trindade	16	25	LIVERWORTS: **Lejeuneaceae** (12 spp.)
Fernando de Noronha	14	18	HORNWORTS: Notothyladaceae (2 spp.) MOSSES: Bryaceae (4 spp.), **Fissidentaceae** (7 spp.)
Ascension	23	45	HORNWORTS: Notothyladaceae (3 spp.) LIVERWORTS: Lejeuneaceae (5 spp.) MOSSES: **Bryaceae** (8 spp.), Fissidentaceae (4 spp.), **Leucobryaceae** (7 spp.), **Pottiaceae** (12 spp.)
Saint Helena	40	63	LIVERWORTS: **Lejeuneaceae** (15 spp.) MOSSES: Bryaceae (7 spp.), **Fissidentaceae** (11 spp.), Leucobryaceae (4 spp.), **Pottiaceae** (9 spp.)
Tristan da Cunha	68	142	LIVERWORTS: **Adelanthaceae** (10 spp.), Anastrophyllaceae (9 spp.), Aneuraceae (13 spp.), Cephaloziellaceae (10 spp.), Lejeuneaceae (14 spp.), Lepidoziaceae (14 spp.), **Lophocoleaceae** (36 spp.), Plagiochilaceae (9 spp.) MOSSES: **Andreaeaceae** (7 spp.), Bartramiaceae (10 spp.), Bryaceae (10 spp.), Ditrichaceae (8 spp.), **Grimmiaceae** (11 spp.), **Polytrichaceae** (6 spp.), Pottiaceae (6 spp.)

**Table 3.** Diversity data for each studied island and archipelago. Spp = Total number of taxa, Ext = Extinct taxa, End = Endemic taxa, Exo = Exotic taxa.

**Islands and archipelagos**	**Spp.**	**Ext**	**End**	**Exo**	**Main references**
Azores	471	?	45	1	Corley et al. (1981), Grolle (1983), Sjögren (2001), Gabriel et al. (2010), Dirkse et al. (2018) and results of this study
Madeira	586	?	56	1	Persson (1939), Sjögren (1975), Sjögren (2001), Sérgio et al. (2006), Sérgio et al. (2008), Dirkse et al. (2018), Fontinha et al. 2020) and results of this study
São Tomé and Príncipe	292	?	19	0	Sérgio and Garcia (2011), Garcia et al. (2012), Garcia et al. 2022, Costa et al. (2024) and results of this study
Cape Verde	207	?	8	?	Frahm et al. (1996), Leyens and Lobin (1996), Patiño–Llorente and Gonzales–Mancebo (2005), Arechavaleta et al. (2005), Medina and Gomes (2015), Dirkse et al. (2018), Garcia et al. (2021), Sim-Sim et al. (2024), Costa and Sérgio (2024) and results of this study
Canary Islands	522	?	47	1	Persson (1939), Eggers (1982), Dirkse et al. (1993), Dirkse et al. (2018), Losada-Lima et al. (2010), Dirkse and Losada-Lima (2011)
Trindade	35	?	0	?	Serafin et al. 2010 Faria et al. 2012 Costa and Rezende 2022
Fernando de Noronha	29	?	1	?	Vital et al. (1991), Serafin et al. (2010), Pereira and Câmara (2015), Costa et al. (2018), Teixeira et al. (2022)
Ascension Island	84	3	15	3	Pressel et al. (2014)
Saint Helena Island	101	0	27	1	Wigginton (2012) and results of this study
Tristan da Cunha	303	0	51	?	Dixon (1960), Wace (1961), Wace and ​​​​​​​Dickson (1965), Váňa and Engel (2013)
