# Supplementary material for: Atlantic oceanic islands and archipelagos: Physical structures, plant diversity, and affinities of the bryofloras
Source: Biodivers Data J. 2025 Feb 28;13:e141577. doi: 10.3897/BDJ.13.e141577 (PMC11889432; doi:10.3897/BDJ.13.e141577)
Supplement: Supplementary material 1 — Table S1. Matrix of presence and absence of liverworts and hornworts taxa [file bdj-13-e141577-s001.docx]

**Supplemental Data**

**Table S1**. Matrix of presence and absence of liverworts and hornworts taxa on the ten islands studied. *= Endemic

| TAXA / ISLANDS OR ARCHIPELAGOS | AZO | MAD | STP | CVE | CAN | TRI | FN | ASC | STH | TRC |
| --- | --- | --- | --- | --- | --- | --- | --- | --- | --- | --- |
| Acanthocoleus aberrans | 1 | 1 | 0 | 0 | 1 | 1 | 0 | 0 | 0 | 0 |
| Acrobolbus azoricus* | 1 | 0 | 0 | 0 | 0 | 0 | 0 | 0 | 0 | 0 |
| Acrobolbus madeirensis* | 0 | 1 | 0 | 0 | 0 | 0 | 0 | 0 | 0 | 0 |
| Acrobolbus ochrophyllus | 0 | 0 | 0 | 0 | 0 | 0 | 0 | 0 | 0 | 1 |
| Acrobolbus viridis | 0 | 0 | 0 | 0 | 0 | 0 | 0 | 0 | 0 | 1 |
| Acrobolbus wilsonii | 1 | 1 | 0 | 0 | 0 | 0 | 0 | 0 | 0 | 0 |
| Acrolejeunea emergens | 0 | 0 | 0 | 1 | 1 | 0 | 0 | 0 | 0 | 0 |
| Adelanthus bisectus | 0 | 0 | 0 | 0 | 0 | 0 | 0 | 0 | 0 | 1 |
| Adelanthus lindenbergianus | 0 | 0 | 0 | 0 | 0 | 0 | 0 | 0 | 0 | 1 |
| Adelanthus lingulatus* | 0 | 0 | 0 | 0 | 0 | 0 | 0 | 0 | 0 | 1 |
| Allisoniella subbipartita | 0 | 0 | 0 | 0 | 0 | 0 | 0 | 0 | 0 | 1 |
| Allisoniella tasmanica | 0 | 0 | 0 | 0 | 0 | 0 | 0 | 0 | 0 | 1 |
| Alophosia azorica* | 1 | 1 | 0 | 0 | 0 | 0 | 0 | 0 | 0 | 0 |
| Amphicephalozia amplexicaulis | 0 | 0 | 0 | 0 | 0 | 0 | 0 | 0 | 0 | 1 |
| Anastrophyllopsis subcomplicata | 0 | 0 | 0 | 0 | 0 | 0 | 0 | 0 | 1 | 1 |
| Anastrophyllopsis involutifolia | 0 | 0 | 0 | 0 | 0 | 0 | 0 | 0 | 0 | 1 |
| Anastrophyllum auritum | 0 | 0 | 0 | 0 | 0 | 0 | 0 | 0 | 0 | 1 |
| Anastrophyllum piligerum | 0 | 0 | 0 | 0 | 0 | 0 | 0 | 1 | 0 | 1 |
| Andrewsianthus marionensis | 0 | 0 | 0 | 0 | 0 | 0 | 0 | 0 | 0 | 1 |
| Aneura latissima | 0 | 1 | 1 | 0 | 0 | 0 | 0 | 0 | 0 | 0 |
| Aneura pinguis | 1 | 1 | 1 | 1 | 1 | 0 | 0 | 0 | 0 | 0 |
| Aneura subcanaliculata* | 0 | 0 | 0 | 0 | 0 | 0 | 0 | 0 | 0 | 1 |
| Anthoceros agrestis | 1 | 1 | 0 | 0 | 1 | 0 | 0 | 0 | 0 | 0 |
| Anthoceros caucasicus | 1 | 1 | 0 | 0 | 0 | 0 | 0 | 0 | 0 | 0 |
| Anthoceros cristatus* | 0 | 0 | 0 | 0 | 0 | 0 | 0 | 1 | 0 | 0 |
| Anthoceros floribundus | 0 | 0 | 0 | 0 | 0 | 0 | 1 | 1 | 0 | 0 |
| Anthoceros patagonicus subsp. gremmenii* | 0 | 0 | 0 | 0 | 0 | 0 | 0 | 0 | 0 | 1 |
| Anthoceros pinnatus | 0 | 1 | 1 | 0 | 0 | 0 | 0 | 0 | 0 | 0 |
| Anthoceros punctatus | 1 | 1 | 0 | 1 | 1 | 1 | 0 | 0 | 0 | 0 |
| Anthoceros tristanianus | 0 | 0 | 0 | 0 | 0 | 0 | 0 | 0 | 0 | 1 |
| Anthoceros sp. | 0 | 0 | 0 | 0 | 0 | 0 | 0 | 0 | 1 | 0 |
| Aphanolejeunea microscopica | 0 | 0 | 0 | 0 | 0 | 0 | 0 | 0 | 0 | 1 |
| Apopellia endiviifolia | 0 | 1 | 0 | 0 | 0 | 0 | 0 | 0 | 0 | 0 |
| Asterella africana | 1 | 1 | 0 | 0 | 1 | 0 | 0 | 0 | 0 | 0 |
| Bazzania azorica* | 1 | 0 | 0 | 0 | 0 | 0 | 0 | 0 | 0 | 0 |
| Bazzania decrescens subsp. molleri | 0 | 0 | 1 | 0 | 0 | 0 | 0 | 0 | 0 | 0 |
| Bazzania nitida | 0 | 0 | 1 | 0 | 0 | 0 | 0 | 0 | 0 | 0 |
| Bazzania peruviana | 0 | 0 | 0 | 0 | 0 | 0 | 0 | 0 | 0 | 1 |
| Bazzania praerupta | 0 | 0 | 0 | 0 | 0 | 0 | 0 | 0 | 1 | 0 |
| Bazzania trilobata | 0 | 1 | 0 | 0 | 0 | 0 | 0 | 0 | 0 | 0 |
| Blasia pusilla | 0 | 1 | 0 | 0 | 0 | 0 | 0 | 0 | 0 | 0 |
| Blepharidophyllum densifolium | 0 | 0 | 0 | 0 | 0 | 0 | 0 | 0 | 0 | 1 |
| Blepharostoma trichophyllum | 1 | 0 | 0 | 0 | 0 | 0 | 0 | 0 | 0 | 0 |
| Brachiolejeunea laxifolia | 0 | 0 | 1 | 0 | 0 | 0 | 0 | 0 | 0 | 0 |
| Calypogeia annabonensis | 0 | 0 | 0 | 0 | 0 | 0 | 0 | 0 | 0 | 1 |
| Calypogeia arguta | 1 | 0 | 1 | 0 | 1 | 0 | 0 | 0 | 0 | 0 |
| Calypogeia azorica* | 1 | 1 | 0 | 0 | 0 | 0 | 0 | 0 | 0 | 0 |
| Calypogeia bidentula | 0 | 0 | 0 | 0 | 0 | 0 | 0 | 0 | 0 | 1 |
| Calypogeia fissa | 1 | 1 | 1 | 0 | 1 | 0 | 0 | 0 | 0 | 0 |
| Calypogeia integristipula | 1 | 0 | 0 | 0 | 0 | 0 | 0 | 0 | 0 | 0 |
| Calypogeia muelleriana | 1 | 1 | 0 | 0 | 0 | 0 | 0 | 0 | 0 | 0 |
| Calypogeia neesiana | 1 | 0 | 0 | 0 | 0 | 0 | 0 | 0 | 0 | 0 |
| Calypogeia peruviana | 0 | 0 | 1 | 0 | 0 | 0 | 0 | 0 | 0 | 0 |
| Calypogeia sphagnicola | 1 | 1 | 0 | 0 | 1 | 0 | 0 | 0 | 0 | 0 |
| Calypogeia suecica | 1 | 0 | 0 | 0 | 1 | 0 | 0 | 0 | 0 | 0 |
| Caudalejeunea africana | 0 | 0 | 0 | 0 | 1 | 0 | 0 | 0 | 0 | 0 |
| Caudalejeunea dusenii | 0 | 0 | 0 | 0 | 1 | 0 | 0 | 0 | 0 | 0 |
| Caudalejeunea hanningtonii | 0 | 0 | 0 | 0 | 1 | 0 | 0 | 0 | 0 | 0 |
| Caudalejeunea lehmanniana | 0 | 0 | 0 | 0 | 1 | 0 | 0 | 0 | 0 | 0 |
| Caudalejeunea yangambiensis | 0 | 0 | 0 | 0 | 1 | 0 | 0 | 0 | 0 | 0 |
| Cephalozia acutiloba | 0 | 0 | 0 | 0 | 0 | 0 | 0 | 0 | 0 | 1 |
| Cephalozia bicuspidata subsp. bicuspidata | 1 | 1 | 0 | 0 | 1 | 0 | 0 | 0 | 0 | 0 |
| Cephalozia bicuspidata subsp. lammersiana | 0 | 1 | 0 | 0 | 0 | 0 | 0 | 0 | 0 | 0 |
| Cephalozia crossii | 0 | 0 | 0 | 0 | 1 | 0 | 0 | 0 | 0 | 0 |
| Cephalozia sanctae-helenae* | 0 | 0 | 0 | 0 | 0 | 0 | 0 | 0 | 1 | 0 |
| Cephalozia tubulata | 0 | 0 | 0 | 0 | 0 | 0 | 0 | 0 | 0 | 1 |
| Cephaloziella baumgartneri | 1 | 1 | 0 | 0 | 1 | 0 | 0 | 0 | 0 | 0 |
| Cephaloziella calyculata | 1 | 1 | 0 | 0 | 1 | 0 | 0 | 0 | 0 | 0 |
| Cephaloziella dentata | 1 | 1 | 0 | 0 | 1 | 0 | 0 | 0 | 0 | 0 |
| Cephaloziella divaricata | 1 | 1 | 1 | 0 | 1 | 0 | 0 | 1 | 0 | 0 |
| Cephaloziella granatensis | 0 | 1 | 0 | 0 | 0 | 0 | 0 | 0 | 0 | 0 |
| Cephaloziella hampeana | 1 | 1 | 0 | 0 | 1 | 0 | 0 | 0 | 0 | 0 |
| Cephaloziella rubella | 1 | 1 | 0 | 0 | 1 | 0 | 0 | 0 | 0 | 0 |
| Cephaloziella stellulifera | 0 | 1 | 0 | 0 | 1 | 0 | 0 | 0 | 0 | 0 |
| Cephaloziella turneri | 1 | 1 | 0 | 0 | 1 | 0 | 0 | 0 | 0 | 0 |
| Cephaloziella umtaliensis | 1 | 1 | 0 | 0 | 1 | 0 | 0 | 0 | 0 | 0 |
| Cephaloziella varians | 0 | 0 | 0 | 0 | 0 | 0 | 0 | 0 | 1 | 0 |
| Cephaloziella sp. | 0 | 0 | 0 | 0 | 0 | 0 | 0 | 0 | 1 | 0 |
| Ceratolejeunea coarina | 0 | 0 | 0 | 0 | 1 | 0 | 0 | 0 | 0 | 0 |
| Ceratolejeunea cornuta | 0 | 0 | 0 | 0 | 1 | 0 | 0 | 0 | 0 | 0 |
| Ceratolejeunea fallax | 0 | 0 | 0 | 0 | 1 | 0 | 0 | 0 | 0 | 0 |
| Ceratolejeunea floribunda* | 0 | 0 | 0 | 0 | 1 | 0 | 0 | 0 | 0 | 0 |
| Ceratolejeunea papuliflora | 0 | 0 | 0 | 0 | 1 | 0 | 0 | 0 | 0 | 0 |
| Ceratolejeunea umbonata | 0 | 0 | 0 | 0 | 1 | 0 | 0 | 0 | 0 | 0 |
| Cheilolejeunea ascensionis* | 0 | 0 | 0 | 0 | 0 | 0 | 0 | 1 | 1 | 0 |
| Cheilolejeunea cedercreutzii* | 0 | 0 | 1 | 1 | 0 | 0 | 0 | 0 | 0 | 0 |
| Cheilolejeunea intertexta | 0 | 0 | 0 | 0 | 1 | 0 | 0 | 0 | 0 | 0 |
| Cheilolejeunea krakakammae | 0 | 0 | 0 | 0 | 1 | 0 | 0 | 0 | 0 | 0 |
| Cheilolejeunea larsenii | 0 | 0 | 0 | 0 | 1 | 0 | 0 | 0 | 0 | 0 |
| Cheilolejeunea microscypha* | 0 | 0 | 0 | 0 | 0 | 0 | 0 | 0 | 1 | 0 |
| Cheilolejeunea montagnei | 0 | 0 | 0 | 0 | 1 | 0 | 0 | 0 | 0 | 0 |
| Cheilolejeunea rigidula | 0 | 0 | 0 | 0 | 1 | 0 | 0 | 0 | 0 | 0 |
| Cheilolejeunea rotalis* | 0 | 0 | 0 | 0 | 0 | 0 | 0 | 0 | 1 | 0 |
| Cheilolejeunea shevockii* | 0 | 0 | 1 | 0 | 0 | 0 | 0 | 0 | 0 | 0 |
| Cheilolejeunea surrepens | 0 | 0 | 0 | 0 | 1 | 0 | 0 | 0 | 0 | 0 |
| Cheilolejeunea trifaria | 0 | 0 | 0 | 0 | 1 | 1 | 0 | 0 | 0 | 0 |
| Cheilolejeunea unciloba | 0 | 0 | 0 | 0 | 0 | 1 | 0 | 0 | 0 | 1 |
| Cheilolejeunea usambarana | 0 | 0 | 0 | 0 | 1 | 0 | 0 | 0 | 0 | 0 |
| Cheilolejeunea xanthocarpa | 0 | 0 | 0 | 0 | 1 | 0 | 0 | 0 | 0 | 0 |
| Chiloscyphus attenuatus | 0 | 0 | 0 | 0 | 0 | 0 | 0 | 0 | 0 | 1 |
| Chiloscyphus austrigenus | 0 | 0 | 0 | 0 | 0 | 0 | 0 | 0 | 0 | 1 |
| Chiloscyphus coadunatus | 0 | 0 | 0 | 0 | 0 | 0 | 0 | 0 | 1 | 1 |
| Chiloscyphus granditextus | 0 | 0 | 0 | 0 | 0 | 0 | 0 | 0 | 0 | 1 |
| Chiloscyphus leptanthus | 0 | 0 | 0 | 0 | 0 | 0 | 0 | 0 | 0 | 1 |
| Chiloscyphus novae-zeelandiae | 0 | 0 | 0 | 0 | 0 | 0 | 0 | 0 | 0 | 1 |
| Chiloscyphus otiphyllus | 0 | 0 | 0 | 0 | 0 | 0 | 0 | 0 | 0 | 1 |
| Chiloscyphus pallescens | 1 | 1 | 0 | 0 | 0 | 0 | 0 | 0 | 0 | 0 |
| Chiloscyphus patulistipus | 0 | 0 | 0 | 0 | 0 | 0 | 0 | 0 | 0 | 1 |
| Chiloscyphus polyanthos | 1 | 1 | 0 | 0 | 0 | 0 | 0 | 0 | 0 | 0 |
| Chiloscyphus serratus | 1 | 0 | 0 | 0 | 0 | 0 | 0 | 0 | 0 | 1 |
| Chiloscyphus textilis | 1 | 0 | 0 | 0 | 0 | 0 | 0 | 0 | 0 | 0 |
| Chiloscyphus tristanianus* | 0 | 0 | 0 | 0 | 0 | 0 | 0 | 0 | 0 | 1 |
| Chiloscyphus wacei* | 0 | 0 | 0 | 0 | 0 | 0 | 0 | 0 | 0 | 1 |
| Cladoradula boryana | 0 | 0 | 1 | 0 | 0 | 0 | 1 | 1 | 0 | 0 |
| Clasmatocolea humilis | 0 | 0 | 0 | 0 | 0 | 0 | 0 | 0 | 0 | 1 |
| Clasmatocolea humilis var. polymorpha* | 0 | 0 | 0 | 0 | 0 | 0 | 0 | 0 | 0 | 1 |
| Clasmatocolea humilis var. suspecta | 0 | 0 | 0 | 0 | 0 | 0 | 0 | 0 | 0 | 1 |
| Clasmatocolea minutiretis | 0 | 0 | 0 | 0 | 0 | 0 | 0 | 0 | 0 | 1 |
| Clasmatocolea navistipula var. parceramosa | 0 | 0 | 0 | 0 | 0 | 0 | 0 | 0 | 0 | 1 |
| Clasmatocolea obvoluta | 0 | 0 | 0 | 0 | 0 | 0 | 0 | 0 | 0 | 1 |
| Clasmatocolea vermicularis | 0 | 0 | 0 | 0 | 0 | 0 | 0 | 1 | 0 | 1 |
| Clevea spathysii | 0 | 0 | 0 | 0 | 1 | 0 | 0 | 0 | 0 | 0 |
| Cololejeunea africana | 0 | 0 | 1 | 0 | 0 | 0 | 0 | 0 | 0 | 0 |
| Cololejeunea azorica | 1 | 1 | 0 | 0 | 0 | 0 | 0 | 0 | 0 | 0 |
| Cololejeunea calcarea | 0 | 1 | 0 | 0 | 0 | 0 | 0 | 0 | 0 | 0 |
| Cololejeunea cardiocarpa | 0 | 0 | 0 | 0 | 0 | 0 | 0 | 1 | 0 | 0 |
| Cololejeunea cuneifolia | 0 | 0 | 1 | 0 | 0 | 0 | 0 | 0 | 0 | 0 |
| Cololejeunea dianae* | 0 | 0 | 0 | 0 | 0 | 0 | 0 | 0 | 1 | 0 |
| Cololejeunea diaphana | 0 | 0 | 1 | 0 | 0 | 0 | 0 | 0 | 0 | 0 |
| Cololejeunea elegans | 0 | 0 | 1 | 0 | 0 | 0 | 0 | 0 | 0 | 0 |
| Cololejeunea grossestyla* | 0 | 0 | 0 | 0 | 0 | 0 | 0 | 0 | 1 | 0 |
| Cololejeunea iradieri | 0 | 0 | 1 | 0 | 0 | 0 | 0 | 0 | 0 | 0 |
| Cololejeunea lanceolata | 0 | 0 | 1 | 0 | 0 | 0 | 0 | 0 | 0 | 0 |
| Cololejeunea leloutrei | 0 | 0 | 1 | 0 | 0 | 0 | 0 | 0 | 0 | 0 |
| Cololejeunea madeirensis* | 1 | 1 | 0 | 0 | 1 | 0 | 0 | 0 | 0 | 0 |
| Cololejeunea marginata | 0 | 0 | 0 | 1 | 0 | 0 | 0 | 0 | 0 | 0 |
| Cololejeunea microscopica | 1 | 0 | 1 | 0 | 0 | 0 | 0 | 0 | 1 | 0 |
| Cololejeunea minutissima | 0 | 0 | 0 | 0 | 0 | 0 | 0 | 0 | 1 | 0 |
| Cololejeunea mocambiquensis | 0 | 0 | 0 | 0 | 1 | 0 | 0 | 0 | 0 | 0 |
| Cololojeunea obliqua | 0 | 0 | 1 | 0 | 0 | 0 | 0 | 0 | 0 | 0 |
| Cololejeunea obtusifolia | 0 | 0 | 0 | 0 | 1 | 0 | 0 | 0 | 0 | 0 |
| Cololejeunea papilliloba | 0 | 0 | 0 | 0 | 1 | 0 | 0 | 0 | 0 | 0 |
| Cololejeunea platyneura | 0 | 0 | 0 | 0 | 1 | 0 | 0 | 0 | 0 | 0 |
| Cololejeunea pseudofloccosa | 0 | 0 | 0 | 0 | 1 | 0 | 0 | 0 | 0 | 0 |
| Cololejeunea pusilla | 0 | 0 | 0 | 0 | 1 | 0 | 0 | 0 | 0 | 0 |
| Cololejeunea sanctae-helenae* | 0 | 0 | 0 | 0 | 0 | 0 | 0 | 0 | 1 | 0 |
| Cololejeunea schaeferi* | 1 | 1 | 0 | 0 | 1 | 0 | 0 | 0 | 0 | 0 |
| Cololejeunea sintenisii | 1 | 1 | 0 | 0 | 1 | 0 | 0 | 0 | 0 | 0 |
| Cololejeunea zenkeri | 0 | 0 | 1 | 0 | 0 | 0 | 0 | 0 | 0 | 0 |
| Colura calderae | 0 | 0 | 1 | 0 | 0 | 0 | 0 | 0 | 0 | 0 |
| Colura calyptrifolia | 1 | 1 | 0 | 0 | 1 | 0 | 0 | 0 | 1 | 1 |
| Colura digitalis | 0 | 0 | 1 | 0 | 0 | 0 | 0 | 0 | 0 | 0 |
| Colura hattoriana | 0 | 0 | 1 | 0 | 0 | 0 | 0 | 0 | 0 | 0 |
| Colura mosenii | 0 | 0 | 1 | 0 | 0 | 0 | 0 | 0 | 0 | 0 |
| Colura obesa | 0 | 0 | 1 | 0 | 0 | 0 | 0 | 0 | 0 | 0 |
| Colura tenuicornis | 0 | 0 | 1 | 0 | 0 | 0 | 0 | 1 | 1 | 0 |
| Colura thomeensis* | 0 | 0 | 1 | 0 | 0 | 0 | 0 | 0 | 0 | 0 |
| Conocephalum conicum | 1 | 1 | 0 | 0 | 1 | 0 | 0 | 0 | 0 | 0 |
| Conocephalum salebrosum | 1 | 1 | 0 | 0 | 0 | 0 | 0 | 0 | 0 | 0 |
| Conoscyphus trapezioides | 0 | 0 | 1 | 0 | 0 | 0 | 0 | 0 | 0 | 0 |
| Corsinia coriandrina | 1 | 1 | 0 | 0 | 1 | 0 | 0 | 0 | 0 | 0 |
| Cryptolophocoela connata | 0 | 0 | 1 | 0 | 0 | 0 | 0 | 0 | 0 | 0 |
| Cryptolophocolea martiana | 1 | 0 | 1 | 0 | 0 | 1 | 1 | 0 | 0 | 0 |
| Cyathodium cavernarum | 0 | 0 | 1 | 1 | 0 | 0 | 0 | 0 | 0 | 0 |
| Cylindrocolea sanctae-helenae* | 0 | 0 | 0 | 0 | 0 | 0 | 0 | 0 | 1 | 0 |
| Deceptifrons plagiochiloides* | 0 | 0 | 0 | 0 | 0 | 0 | 0 | 0 | 0 | 1 |
| Dendroceros adglutinatus* | 0 | 0 | 0 | 0 | 0 | 0 | 0 | 0 | 1 | 0 |
| Dendroceros crispatus | 0 | 0 | 1 | 0 | 0 | 0 | 0 | 0 | 0 | 0 |
| Dendroceros herasii | 0 | 0 | 1 | 0 | 0 | 0 | 0 | 0 | 0 | 0 |
| Dendroceros paivae* | 0 | 0 | 1 | 0 | 0 | 0 | 0 | 0 | 0 | 0 |
| Dibrachiella africana | 0 | 0 | 1 | 0 | 0 | 0 | 0 | 0 | 0 | 0 |
| Dibrachiella autoica | 0 | 0 | 1 | 0 | 0 | 0 | 0 | 0 | 0 | 0 |
| Diplasiolejeunea aulae | 0 | 0 | 1 | 0 | 0 | 0 | 0 | 0 | 0 | 0 |
| Diplasiolejeunea cavifolia | 0 | 0 | 1 | 0 | 0 | 0 | 0 | 0 | 0 | 0 |
| Diplophyllum albicans | 1 | 1 | 0 | 0 | 1 | 0 | 0 | 0 | 0 | 0 |
| Diplophyllum obtusifolium | 0 | 1 | 0 | 0 | 0 | 0 | 0 | 0 | 0 | 0 |
| Drepanolejeunea araucariae | 0 | 0 | 0 | 0 | 0 | 0 | 0 | 0 | 0 | 1 |
| Drepanolejeunea capulata | 0 | 0 | 1 | 0 | 0 | 0 | 0 | 0 | 0 | 0 |
| Drepanolejeunea cultrella | 0 | 0 | 1 | 0 | 0 | 0 | 0 | 0 | 0 | 0 |
| Drepanolejeunea hamatifolia | 1 | 1 | 0 | 0 | 1 | 0 | 0 | 0 | 0 | 0 |
| Drepanolejeunea mosenii | 0 | 0 | 1 | 0 | 0 | 0 | 0 | 0 | 0 | 0 |
| Drepanolejeunea physifolia | 0 | 0 | 1 | 0 | 0 | 0 | 0 | 0 | 0 | 0 |
| Dumortiera hirsuta | 1 | 1 | 1 | 0 | 1 | 0 | 0 | 0 | 0 | 0 |
| Exormotheca martins-loussaone | 0 | 0 | 0 | 1 | 0 | 0 | 0 | 0 | 0 | 0 |
| Exormotheca pustulosa | 1 | 1 | 0 | 1 | 1 | 0 | 0 | 0 | 1 | 0 |
| Folioceros incurvus | 0 | 0 | 1 | 0 | 0 | 0 | 0 | 0 | 0 | 0 |
| Fossombronia angulosa | 1 | 1 | 0 | 1 | 1 | 0 | 0 | 0 | 0 | 0 |
| Fossombronia caespitiformis subsp. caespitiformis | 1 | 1 | 0 | 0 | 1 | 0 | 0 | 0 | 0 | 0 |
| Fossombronia caespitiformis subsp. multispira | 1 | 1 | 0 | 1 | 1 | 0 | 0 | 0 | 0 | 0 |
| Fossombronia echinata | 1 | 1 | 0 | 0 | 1 | 0 | 0 | 0 | 0 | 0 |
| Fossombronia fernandeziensis | 0 | 0 | 0 | 0 | 0 | 0 | 0 | 0 | 0 | 1 |
| Fossombronia husnotii | 0 | 0 | 0 | 0 | 0 | 0 | 0 | 1 | 0 | 0 |
| Fossombronia indica | 0 | 0 | 1 | 0 | 0 | 0 | 0 | 1 | 0 | 0 |
| Fossombronia pusilla | 1 | 1 | 0 | 1 | 1 | 0 | 0 | 0 | 0 | 0 |
| Fossombronia wondraczekii | 1 | 0 | 0 | 0 | 0 | 0 | 0 | 0 | 0 | 0 |
| Fossombronia sp. | 0 | 0 | 0 | 0 | 0 | 0 | 0 | 0 | 1 | 0 |
| Frullania acicularis* | 1 | 1 | 0 | 0 | 0 | 0 | 0 | 0 | 0 | 0 |
| Frullania angulata | 0 | 0 | 1 | 0 | 0 | 0 | 0 | 1 | 0 | 0 |
| Frullania apicalis | 0 | 0 | 1 | 0 | 0 | 0 | 0 | 1 | 0 | 0 |
| Frullania apiculata | 0 | 0 | 1 | 0 | 0 | 0 | 0 | 0 | 0 | 0 |
| Frullania azorica* | 1 | 1 | 0 | 0 | 1 | 0 | 0 | 0 | 0 | 0 |
| Frullania beyrichiana | 0 | 0 | 0 | 0 | 0 | 1 | 0 | 0 | 0 | 0 |
| Frullania brasiliensis | 0 | 0 | 0 | 0 | 0 | 0 | 0 | 0 | 0 | 1 |
| Frullania caffraria | 0 | 0 | 1 | 0 | 0 | 0 | 0 | 0 | 0 | 0 |
| Frullania calcarifera* | 0 | 0 | 0 | 0 | 1 | 0 | 0 | 0 | 0 | 0 |
| Frullania caulisequa | 0 | 0 | 0 | 0 | 0 | 1 | 0 | 0 | 0 | 0 |
| Frullania depressa | 0 | 0 | 1 | 0 | 0 | 0 | 0 | 0 | 1 | 0 |
| Frullania dilatata | 0 | 1 | 0 | 1 | 1 | 0 | 0 | 0 | 0 | 0 |
| Frullania diptera | 0 | 0 | 1 | 0 | 0 | 0 | 0 | 0 | 0 | 0 |
| Frullania ecklonii | 0 | 0 | 1 | 0 | 0 | 0 | 0 | 0 | 0 | 0 |
| Frullania ericoides | 0 | 1 | 1 | 1 | 1 | 0 | 1 | 1 | 0 | 0 |
| Frullania fragilifolia | 0 | 0 | 0 | 0 | 1 | 0 | 0 | 0 | 0 | 0 |
| Frullania gibbosa | 0 | 0 | 0 | 0 | 0 | 1 | 0 | 0 | 0 | 0 |
| Frullania kunzei | 0 | 0 | 0 | 0 | 0 | 1 | 0 | 0 | 0 | 0 |
| Frullania lindenbergii | 0 | 0 | 0 | 0 | 0 | 0 | 0 | 0 | 0 | 1 |
| Frullania magellanica | 0 | 0 | 0 | 0 | 0 | 0 | 0 | 0 | 0 | 1 |
| Frullania magellanica subsp. tristaniana* | 0 | 0 | 0 | 0 | 0 | 0 | 0 | 0 | 0 | 1 |
| Frullania microphylla* | 1 | 1 | 0 | 0 | 1 | 0 | 0 | 0 | 0 | 0 |
| Frullania obscura | 0 | 0 | 1 | 0 | 0 | 0 | 0 | 0 | 0 | 0 |
| Frullania obscurifolia | 0 | 0 | 1 | 0 | 0 | 0 | 0 | 0 | 0 | 0 |
| Frullania polysticta* | 0 | 1 | 0 | 0 | 1 | 0 | 0 | 0 | 0 | 0 |
| Frullania purpurea | 0 | 0 | 1 | 0 | 0 | 0 | 0 | 0 | 0 | 0 |
| Frullania riojaneirensis | 0 | 0 | 1 | 0 | 0 | 0 | 0 | 0 | 0 | 0 |
| Frullania rostrata | 0 | 0 | 0 | 0 | 0 | 0 | 0 | 0 | 0 | 1 |
| Frullania sergioae* | 0 | 1 | 0 | 0 | 0 | 0 | 0 | 0 | 0 | 0 |
| Frullania serrata | 0 | 0 | 1 | 0 | 0 | 0 | 0 | 0 | 0 | 0 |
| Frullania socotrana | 0 | 0 | 0 | 1 | 0 | 0 | 0 | 0 | 0 | 0 |
| Frullania spongiosa | 0 | 0 | 1 | 1 | 0 | 0 | 0 | 0 | 0 | 0 |
| Frullania stipatiloba | 0 | 0 | 0 | 0 | 0 | 0 | 0 | 0 | 0 | 1 |
| Frullania tamarisci | 1 | 1 | 0 | 1 | 1 | 0 | 0 | 0 | 0 | 0 |
| Frullania teneriffae | 1 | 1 | 0 | 0 | 1 | 0 | 0 | 0 | 0 | 0 |
| Frullanoides corticalis | 0 | 0 | 0 | 0 | 0 | 1 | 0 | 0 | 0 | 0 |
| Fuscocephaloziopsis catenulata | 0 | 1 | 0 | 0 | 0 | 0 | 0 | 0 | 0 | 0 |
| Fuscocephaloziopsis connivens | 1 | 1 | 0 | 0 | 0 | 0 | 0 | 0 | 0 | 0 |
| Fuscocephaloziopsis connivens subsp. fissa | 0 | 0 | 0 | 0 | 0 | 0 | 0 | 0 | 0 | 1 |
| Fuscocephaloziopsis crassifolia | 1 | 1 | 0 | 0 | 0 | 0 | 0 | 0 | 0 | 0 |
| Fuscocephaloziopsis lunulifolia | 1 | 1 | 0 | 0 | 0 | 0 | 0 | 0 | 0 | 0 |
| Fuscocephaloziopsis pleniceps | 0 | 0 | 0 | 0 | 0 | 0 | 0 | 0 | 0 | 1 |
| Geocalyx graveolens | 1 | 1 | 0 | 0 | 0 | 0 | 0 | 0 | 0 | 0 |
| Gongylanthus ericetorum | 1 | 1 | 0 | 0 | 1 | 0 | 0 | 0 | 0 | 0 |
| Gymnocolea inflata | 1 | 1 | 0 | 0 | 1 | 0 | 0 | 0 | 0 | 0 |
| Gymnomitrion adustum | 1 | 1 | 0 | 0 | 0 | 0 | 0 | 0 | 0 | 0 |
| Harpalejeunea hasselii | 0 | 0 | 0 | 0 | 0 | 0 | 0 | 0 | 0 | 1 |
| Harpalejeunea marginalis | 0 | 0 | 0 | 0 | 0 | 0 | 0 | 0 | 0 | 1 |
| Harpalejeunea molleri* | 1 | 1 | 1 | 0 | 1 | 0 | 0 | 0 | 0 | 1 |
| Herbertus dicranus | 1 | 0 | 1 | 0 | 0 | 0 | 0 | 0 | 0 | 0 |
| Herbertus juniperoideus | 0 | 0 | 1 | 0 | 0 | 0 | 0 | 0 | 0 | 0 |
| Herbertus oldfieldianus | 0 | 0 | 0 | 0 | 0 | 0 | 0 | 0 | 0 | 1 |
| Herbertus runcinatus | 0 | 0 | 0 | 0 | 0 | 0 | 0 | 0 | 0 | 1 |
| Herbertus sendtneri | 1 | 0 | 0 | 0 | 0 | 0 | 0 | 0 | 0 | 1 |
| Herzogobryum atrocapillum | 0 | 0 | 0 | 0 | 0 | 0 | 0 | 0 | 0 | 1 |
| Herzogobryum molle | 0 | 0 | 0 | 0 | 0 | 0 | 0 | 0 | 0 | 1 |
| Heteroscyphus denticulatus* | 1 | 1 | 0 | 0 | 1 | 0 | 0 | 0 | 0 | 0 |
| Heteroscyphus dubius | 0 | 0 | 1 | 0 | 0 | 0 | 0 | 0 | 0 | 0 |
| Heteroscyphus integrifolius | 0 | 0 | 0 | 0 | 0 | 0 | 0 | 0 | 0 | 1 |
| Hyalolepidozia bicuspidata | 0 | 0 | 0 | 0 | 0 | 0 | 0 | 0 | 0 | 1 |
| Hygrobiella laxifolia | 1 | 1 | 0 | 0 | 0 | 0 | 0 | 0 | 0 | 0 |
| Isotachis aubertii | 0 | 0 | 0 | 0 | 0 | 0 | 0 | 1 | 0 | 0 |
| Isotachis grossidens | 0 | 0 | 0 | 0 | 0 | 0 | 0 | 0 | 0 | 1 |
| Isotachis humectata | 0 | 0 | 0 | 0 | 0 | 0 | 0 | 0 | 0 | 1 |
| Isotachis spegazziniana | 0 | 0 | 0 | 0 | 0 | 0 | 0 | 0 | 0 | 1 |
| Jensenia difformis | 0 | 0 | 0 | 0 | 0 | 0 | 0 | 0 | 0 | 1 |
| Jensenia spinosa | 0 | 0 | 0 | 0 | 0 | 0 | 0 | 0 | 1 | 0 |
| Jubula hutchinsiae subsp. hutchinsiae | 1 | 1 | 0 | 0 | 1 | 0 | 0 | 0 | 0 | 0 |
| Jungermannia atrovirens | 1 | 1 | 0 | 0 | 1 | 0 | 0 | 0 | 0 | 0 |
| Jungermannia pumila | 1 | 1 | 0 | 0 | 1 | 0 | 0 | 0 | 0 | 0 |
| Kurzia capillaris | 0 | 0 | 0 | 0 | 0 | 0 | 0 | 0 | 0 | 1 |
| Kurzia nemoides* | 0 | 0 | 0 | 0 | 0 | 0 | 0 | 0 | 1 | 0 |
| Kurzia pauciflora | 1 | 1 | 0 | 0 | 0 | 0 | 0 | 0 | 0 | 0 |
| Kurzia setiformis | 0 | 0 | 0 | 0 | 0 | 0 | 0 | 0 | 0 | 1 |
| Leiomitra elegans | 0 | 0 | 0 | 0 | 0 | 0 | 0 | 0 | 0 | 1 |
| Lejeunea abyssinica | 0 | 0 | 1 | 0 | 0 | 0 | 0 | 0 | 0 | 0 |
| Lejeunea acuta | 0 | 0 | 1 | 0 | 0 | 0 | 0 | 0 | 0 | 0 |
| Lejeunea adpressa | 0 | 0 | 0 | 0 | 0 | 1 | 0 | 0 | 0 | 0 |
| Lejeunea anisophylla | 0 | 0 | 1 | 0 | 0 | 0 | 0 | 0 | 0 | 0 |
| Lejeunea aphanes | 0 | 0 | 0 | 0 | 0 | 0 | 0 | 0 | 1 | 0 |
| Lejeunea brenanii | 0 | 0 | 1 | 0 | 0 | 0 | 0 | 0 | 0 | 0 |
| Lejeunea canariensis* | 0 | 1 | 0 | 0 | 1 | 0 | 0 | 0 | 0 | 0 |
| Lejeunea capensis | 0 | 0 | 1 | 1 | 1 | 0 | 0 | 0 | 0 | 0 |
| Lejeunea cavifolia | 0 | 1 | 0 | 0 | 1 | 0 | 0 | 0 | 0 | 0 |
| Lejeunea conformis | 0 | 0 | 1 | 0 | 0 | 0 | 0 | 0 | 0 | 0 |
| Lejeunea eckloniana | 1 | 1 | 1 | 1 | 1 | 0 | 0 | 0 | 1 | 1 |
| Lejeunea flava | 1 | 1 | 1 | 1 | 1 | 1 | 0 | 0 | 0 | 1 |
| Lejeunea flava subsp. tabularis | 0 | 0 | 1 | 0 | 0 | 0 | 0 | 0 | 0 | 0 |
| Lejeunea grossecristata | 0 | 0 | 1 | 0 | 0 | 0 | 0 | 0 | 0 | 0 |
| Lejeunea helenae | 0 | 0 | 1 | 0 | 0 | 0 | 0 | 0 | 0 | 0 |
| Lejeunea hibernica | 1 | 1 | 0 | 0 | 0 | 0 | 0 | 0 | 0 | 0 |
| Lejeunea ibadana | 0 | 0 | 1 | 0 | 0 | 0 | 0 | 0 | 0 | 0 |
| Lejeunea jungneri | 0 | 0 | 1 | 0 | 0 | 0 | 0 | 0 | 0 | 0 |
| Lejeunea laetevirens | 0 | 0 | 0 | 0 | 0 | 0 | 1 | 1 | 0 | 0 |
| Lejeunea lamacerina | 1 | 1 | 0 | 1 | 1 | 0 | 0 | 0 | 0 | 1 |
| Lejeunea longicollis* | 0 | 0 | 0 | 0 | 0 | 0 | 0 | 0 | 0 | 1 |
| Lejeunea lyratiflora | 0 | 0 | 1 | 0 | 0 | 0 | 0 | 0 | 0 | 0 |
| Lejeunea magnoliae | 0 | 0 | 0 | 0 | 0 | 1 | 0 | 0 | 0 | 0 |
| Lejeunea mandonii* | 0 | 1 | 0 | 0 | 1 | 0 | 0 | 0 | 0 | 0 |
| Lejeunea obtusata | 0 | 0 | 1 | 0 | 0 | 0 | 0 | 0 | 0 | 0 |
| Lejeunea okomuensis | 0 | 0 | 1 | 0 | 0 | 0 | 0 | 0 | 0 | 0 |
| Lejeunea papilionacea | 0 | 0 | 1 | 0 | 0 | 0 | 0 | 0 | 0 | 0 |
| Lejeunea patagonica | 0 | 0 | 0 | 0 | 0 | 0 | 0 | 0 | 0 | 1 |
| Lejeunea patens | 1 | 1 | 0 | 0 | 0 | 0 | 0 | 0 | 0 | 0 |
| Lejeunea phyllobola | 0 | 0 | 1 | 0 | 0 | 0 | 0 | 0 | 0 | 0 |
| Lejeunea pulchriflora | 0 | 0 | 1 | 0 | 0 | 0 | 0 | 0 | 0 | 0 |
| Lejeunea ramosissima | 0 | 0 | 1 | 0 | 0 | 0 | 0 | 0 | 0 | 0 |
| Lejeunea sanctae-helenae* | 0 | 0 | 0 | 0 | 0 | 0 | 0 | 0 | 1 | 0 |
| Lejeunea setacea | 0 | 0 | 1 | 0 | 0 | 0 | 0 | 0 | 0 | 0 |
| Lejeunea tuberculosa | 0 | 0 | 1 | 0 | 0 | 0 | 0 | 0 | 0 | 0 |
| Lepicolea ochroleuca | 0 | 0 | 0 | 0 | 0 | 0 | 0 | 0 | 0 | 1 |
| Lepidozia cupressina | 1 | 1 | 0 | 0 | 1 | 0 | 0 | 0 | 0 | 0 |
| Lepidozia cupressina var. africana | 0 | 0 | 0 | 0 | 0 | 0 | 0 | 0 | 1 | 1 |
| Lepidozia laevifolia | 0 | 0 | 0 | 0 | 0 | 0 | 0 | 0 | 0 | 1 |
| Lepidozia pearsonii | 1 | 0 | 0 | 0 | 0 | 0 | 0 | 0 | 0 | 0 |
| Lepidozia reptans | 1 | 1 | 0 | 0 | 0 | 0 | 0 | 0 | 0 | 0 |
| Lepidozia stuhlmannii | 1 | 0 | 0 | 0 | 0 | 0 | 0 | 0 | 0 | 0 |
| Lepidozia succida | 0 | 0 | 1 | 0 | 0 | 0 | 0 | 0 | 0 | 0 |
| Lepidozia ubangiensis | 0 | 0 | 1 | 0 | 0 | 0 | 0 | 0 | 0 | 0 |
| Leptolejeunea astroidea | 0 | 0 | 1 | 0 | 0 | 0 | 0 | 0 | 0 | 0 |
| Leptolejeunea epiphylla | 0 | 0 | 1 | 0 | 0 | 0 | 0 | 0 | 0 | 0 |
| Leptolejeunea maculata | 0 | 0 | 1 | 0 | 0 | 0 | 0 | 0 | 0 | 0 |
| Leptoscyphus aequatus | 0 | 0 | 0 | 0 | 0 | 0 | 0 | 0 | 0 | 1 |
| Leptoscyphus chilensis | 0 | 0 | 0 | 0 | 0 | 0 | 0 | 0 | 0 | 1 |
| Leptoscyphus cuneifolius | 1 | 1 | 0 | 0 | 0 | 0 | 0 | 0 | 0 | 0 |
| Leptoscyphus cuneifolius subsp. fragilis | 0 | 0 | 0 | 0 | 0 | 0 | 0 | 0 | 0 | 1 |
| Leptoscyphus expansus | 0 | 0 | 0 | 0 | 0 | 0 | 0 | 0 | 0 | 1 |
| Leptoscyphus porphyrius subsp. azoricus* | 1 | 0 | 0 | 0 | 0 | 0 | 0 | 0 | 0 | 0 |
| Lethocolea radicosa | 0 | 0 | 0 | 0 | 0 | 0 | 0 | 0 | 0 | 1 |
| Liochlaena lanceolata | 0 | 1 | 0 | 0 | 1 | 0 | 0 | 0 | 0 | 0 |
| Lophocolea anomala | 0 | 0 | 0 | 0 | 0 | 0 | 0 | 0 | 0 | 1 |
| Lophocolea ascensionis* | 0 | 0 | 0 | 0 | 0 | 0 | 0 | 1 | 0 | 0 |
| Lophocolea bidentata | 1 | 1 | 1 | 1 | 1 | 0 | 0 | 0 | 0 | 1 |
| Lophocolea coadunata | 1 | 0 | 0 | 0 | 1 | 0 | 0 | 0 | 0 | 0 |
| Lophocolea difformis | 0 | 0 | 1 | 0 | 0 | 0 | 0 | 0 | 1 | 0 |
| Lophocolea fragrans | 1 | 1 | 0 | 0 | 1 | 0 | 0 | 0 | 0 | 0 |
| Lophocolea heterophylla | 1 | 1 | 0 | 0 | 1 | 0 | 0 | 0 | 0 | 0 |
| Lophocolea humistrata* | 0 | 0 | 0 | 0 | 0 | 0 | 0 | 0 | 1 | 0 |
| Lophocolea minor | 1 | 1 | 0 | 0 | 1 | 0 | 0 | 0 | 0 | 0 |
| Lophocolea muricata | 0 | 0 | 0 | 0 | 0 | 0 | 0 | 0 | 0 | 1 |
| Lophocolea sabuletorum | 0 | 0 | 0 | 0 | 0 | 0 | 0 | 0 | 0 | 1 |
| Lophocolea semiteres | 0 | 0 | 0 | 0 | 0 | 0 | 0 | 0 | 0 | 1 |
| Lopholejeunea nigricans | 0 | 0 | 1 | 0 | 0 | 0 | 0 | 0 | 0 | 0 |
| Lopholejeunea subfusca | 0 | 0 | 1 | 0 | 0 | 0 | 0 | 0 | 0 | 0 |
| Lophonardia tristaniana* | 0 | 0 | 0 | 0 | 0 | 0 | 0 | 0 | 0 | 1 |
| Lophozia longiflora | 1 | 0 | 0 | 0 | 0 | 0 | 0 | 0 | 0 | 0 |
| Lophozia ventricosa | 1 | 0 | 0 | 0 | 0 | 0 | 0 | 0 | 0 | 0 |
| Lophoziopsis excisa | 0 | 0 | 0 | 0 | 1 | 0 | 0 | 0 | 0 | 1 |
| Lunularia cruciata | 1 | 1 | 0 | 1 | 1 | 0 | 0 | 0 | 0 | 1 |
| Mannia androgyna | 1 | 1 | 0 | 1 | 1 | 0 | 0 | 0 | 0 | 0 |
| Mannia fragrans | 0 | 1 | 0 | 0 | 0 | 0 | 0 | 0 | 0 | 0 |
| Marchantia berteroana | 0 | 0 | 0 | 0 | 0 | 0 | 0 | 1 | 1 | 1 |
| Marchantia debilis | 0 | 0 | 1 | 0 | 0 | 0 | 0 | 0 | 0 | 0 |
| Marchantia paleaceae | 1 | 1 | 0 | 1 | 1 | 0 | 0 | 0 | 0 | 0 |
| Marchantia pappeana subsp. pappeana | 0 | 0 | 1 | 1 | 0 | 0 | 0 | 0 | 0 | 0 |
| Marchantia polymorpha | 1 | 1 | 0 | 1 | 1 | 0 | 0 | 0 | 0 | 0 |
| Marchantia polymorpha subsp. montivagans | 0 | 1 | 0 | 0 | 0 | 0 | 0 | 0 | 0 | 0 |
| Marchantia polymorpha subsp. ruderalis | 0 | 1 | 0 | 0 | 0 | 0 | 0 | 0 | 0 | 0 |
| Marchesinia brachiata | 0 | 0 | 0 | 0 | 0 | 1 | 0 | 0 | 1 | 0 |
| Marchesinia excavata | 0 | 0 | 1 | 0 | 0 | 0 | 0 | 0 | 0 | 0 |
| Marchesinia mackaii | 1 | 1 | 0 | 1 | 1 | 0 | 0 | 0 | 0 | 0 |
| Marchesinia principensis* | 0 | 0 | 1 | 0 | 0 | 0 | 0 | 0 | 0 | 0 |
| Marsupella aquatica | 1 | 0 | 0 | 0 | 0 | 0 | 0 | 0 | 0 | 0 |
| Marsupella emarginata | 1 | 1 | 0 | 0 | 1 | 0 | 0 | 0 | 0 | 0 |
| Marsupella funckii | 1 | 1 | 0 | 0 | 1 | 0 | 0 | 0 | 0 | 0 |
| Marsupella profunda | 1 | 1 | 0 | 0 | 1 | 0 | 0 | 0 | 0 | 0 |
| Marsupella sparsifolia | 1 | 0 | 0 | 0 | 0 | 0 | 0 | 0 | 0 | 0 |
| Marsupella sphacelata | 1 | 0 | 0 | 0 | 0 | 0 | 0 | 0 | 0 | 0 |
| Marsupella sprucei | 0 | 1 | 0 | 0 | 1 | 0 | 0 | 0 | 0 | 0 |
| Mastigophora diclados | 0 | 0 | 1 | 0 | 0 | 0 | 0 | 1 | 0 | 1 |
| Megaceros flagellaris | 0 | 0 | 1 | 0 | 0 | 0 | 0 | 0 | 0 | 0 |
| Metalejeunea cucullata | 0 | 0 | 1 | 0 | 0 | 0 | 0 | 0 | 0 | 0 |
| Metzgeria albinea | 0 | 0 | 1 | 0 | 0 | 0 | 0 | 0 | 0 | 0 |
| Metzgeria ciliata | 0 | 0 | 0 | 0 | 0 | 0 | 0 | 0 | 0 | 1 |
| Metzgeria consanguinea | 1 | 1 | 0 | 0 | 1 | 0 | 0 | 0 | 0 | 1 |
| Metzgeria epiphylla | 0 | 0 | 0 | 0 | 0 | 0 | 0 | 0 | 0 | 1 |
| Metzgeria furcata | 1 | 1 | 1 | 0 | 1 | 0 | 0 | 0 | 0 | 0 |
| Metzgeria leptoneura | 1 | 1 | 1 | 0 | 1 | 0 | 0 | 0 | 0 | 1 |
| Metzgeria lindbergii | 0 | 1 | 1 | 0 | 1 | 0 | 0 | 0 | 0 | 0 |
| Metzgeria cf. madagassa | 0 | 0 | 1 | 0 | 0 | 0 | 0 | 0 | 0 | 0 |
| Metzgeria nudifrons | 0 | 0 | 1 | 0 | 0 | 0 | 0 | 0 | 0 | 0 |
| Metzgeria violacea | 1 | 1 | 0 | 0 | 0 | 0 | 0 | 0 | 0 | 1 |
| Microlejeunea aff. acutifolia | 0 | 0 | 1 | 0 | 0 | 0 | 0 | 0 | 0 | 0 |
| Microlejeunea africana | 0 | 0 | 1 | 0 | 0 | 0 | 0 | 1 | 1 | 0 |
| Microlejeunea ankasica | 0 | 0 | 1 | 0 | 0 | 0 | 0 | 0 | 0 | 0 |
| Microlejeunea bullata | 0 | 0 | 0 | 0 | 0 | 1 | 0 | 0 | 0 | 1 |
| Microlejeunea kamerunensis | 0 | 0 | 1 | 0 | 0 | 0 | 0 | 0 | 0 | 0 |
| Microlejeunea ulicina | 1 | 1 | 0 | 1 | 1 | 0 | 0 | 0 | 0 | 1 |
| Mnioloma fuscum | 1 | 0 | 0 | 0 | 0 | 0 | 0 | 0 | 1 | 1 |
| Myriocoleopsis minutissima | 1 | 1 | 0 | 1 | 1 | 0 | 0 | 0 | 0 | 0 |
| Nardia geoscyphus | 1 | 1 | 0 | 0 | 1 | 0 | 0 | 0 | 0 | 0 |
| Nardia scalaris | 1 | 1 | 0 | 0 | 1 | 0 | 0 | 0 | 0 | 0 |
| Neohodgsonia mirabilis | 0 | 0 | 0 | 0 | 0 | 0 | 0 | 0 | 0 | 1 |
| Noteroclada confluens | 0 | 0 | 0 | 1 | 0 | 0 | 0 | 0 | 0 | 1 |
| Nothogymnomitrion erosum | 0 | 0 | 0 | 0 | 0 | 0 | 0 | 0 | 0 | 1 |
| Notoscyphus lutescens | 0 | 0 | 1 | 0 | 0 | 0 | 0 | 0 | 0 | 0 |
| Notothylas breutelii | 0 | 0 | 0 | 0 | 0 | 0 | 1 | 0 | 0 | 0 |
| Notothylas javanica | 0 | 0 | 0 | 0 | 0 | 0 | 1 | 0 | 0 | 0 |
| Notothylas orbicularis | 0 | 0 | 0 | 0 | 0 | 0 | 1 | 0 | 0 | 0 |
| Nowellia curvifolia | 1 | 1 | 0 | 0 | 0 | 0 | 0 | 0 | 0 | 0 |
| Odontolejeunea lunulata | 0 | 0 | 1 | 0 | 0 | 0 | 0 | 0 | 0 | 0 |
| Odontoschisma denudatum | 1 | 1 | 0 | 0 | 0 | 0 | 0 | 0 | 0 | 0 |
| Odontoschisma francisci | 1 | 1 | 0 | 0 | 0 | 0 | 0 | 0 | 0 | 0 |
| Odontoschisma sphagni | 1 | 1 | 0 | 0 | 0 | 0 | 0 | 0 | 0 | 0 |
| Oxymitra incrassata | 0 | 1 | 0 | 0 | 1 | 0 | 0 | 0 | 0 | 0 |
| Pachyglossa dissitifolia | 0 | 0 | 0 | 0 | 0 | 0 | 0 | 0 | 0 | 1 |
| Pachyglossa exilis | 0 | 0 | 0 | 0 | 0 | 0 | 0 | 0 | 0 | 1 |
| Pachyglossa spegazziniana | 0 | 0 | 0 | 0 | 0 | 0 | 0 | 0 | 0 | 1 |
| Pallavicinia lyellii | 1 | 1 | 0 | 1 | 0 | 0 | 0 | 0 | 0 | 0 |
| Paracromastigum macrostipum | 0 | 0 | 0 | 0 | 0 | 0 | 0 | 0 | 0 | 1 |
| Paracromastigum subsimplex | 0 | 0 | 0 | 0 | 0 | 0 | 0 | 0 | 0 | 1 |
| Paracromastigum tristanianum | 0 | 0 | 0 | 0 | 0 | 0 | 0 | 0 | 0 | 1 |
| Pellia epiphylla | 1 | 1 | 0 | 1 | 0 | 0 | 0 | 0 | 0 | 0 |
| Perdusenia rheophila | 0 | 0 | 0 | 0 | 0 | 0 | 0 | 0 | 0 | 1 |
| Phaeoceros carolinianus | 1 | 1 | 1 | 1 | 1 | 0 | 0 | 1 | 1 | 0 |
| Phaeoceros dendroceroides | 0 | 0 | 0 | 0 | 0 | 0 | 0 | 0 | 0 | 1 |
| Phaeoceros evanidus* | 0 | 0 | 0 | 0 | 0 | 0 | 0 | 1 | 0 | 0 |
| Phaeoceros laevis | 1 | 1 | 0 | 0 | 1 | 0 | 0 | 1 | 0 | 1 |
| Phaeomegaceros plicatus* | 0 | 0 | 0 | 0 | 0 | 0 | 0 | 0 | 0 | 1 |
| Plagiochasma eximium | 0 | 0 | 0 | 1 | 0 | 0 | 0 | 0 | 0 | 0 |
| Plagiochasma rupestre | 1 | 1 | 0 | 1 | 1 | 0 | 0 | 1 | 1 | 0 |
| Plagiochila africana | 0 | 0 | 1 | 0 | 0 | 0 | 0 | 0 | 0 | 0 |
| Plagiochila angulata | 0 | 0 | 0 | 0 | 0 | 0 | 0 | 0 | 0 | 1 |
| Plagiochila badia | 0 | 0 | 0 | 0 | 0 | 0 | 0 | 0 | 0 | 1 |
| Plagiochila barteri | 0 | 0 | 1 | 0 | 0 | 0 | 0 | 0 | 0 | 0 |
| Plagiochila barteri var. valida | 0 | 0 | 1 | 0 | 0 | 0 | 0 | 0 | 0 | 0 |
| Plagiochila bicuspidata | 0 | 0 | 0 | 0 | 0 | 0 | 0 | 0 | 0 | 1 |
| Plagiochila bifaria | 1 | 1 | 0 | 0 | 1 | 0 | 0 | 0 | 0 | 0 |
| Plagiochila brunneola | 0 | 0 | 1 | 0 | 0 | 0 | 0 | 0 | 0 | 0 |
| Plagiochila corrugata | 0 | 0 | 0 | 0 | 0 | 1 | 0 | 0 | 0 | 0 |
| Plagiochila equitans | 0 | 0 | 0 | 0 | 0 | 0 | 0 | 0 | 0 | 1 |
| Plagiochila exigua | 1 | 1 | 0 | 0 | 1 | 0 | 0 | 0 | 0 | 0 |
| Plagiochila flabellata | 0 | 0 | 1 | 0 | 0 | 0 | 0 | 0 | 0 | 0 |
| Plagiochila fusifera | 0 | 0 | 1 | 0 | 0 | 0 | 0 | 0 | 0 | 0 |
| Plagiochila gibbiflora* | 0 | 0 | 1 | 0 | 0 | 0 | 0 | 0 | 0 | 0 |
| Plagiochila heterostipa | 0 | 0 | 1 | 0 | 0 | 0 | 0 | 0 | 0 | 0 |
| Plagiochila hookeriana | 0 | 0 | 0 | 0 | 0 | 0 | 0 | 0 | 0 | 1 |
| Plagiochila infuscata* | 0 | 0 | 0 | 0 | 0 | 0 | 0 | 0 | 0 | 1 |
| Plagiochila integerrima | 0 | 1 | 0 | 0 | 0 | 0 | 0 | 0 | 0 | 0 |
| Plagiochila lechleri | 0 | 0 | 0 | 0 | 0 | 0 | 0 | 0 | 0 | 1 |
| Plagiochila loloensis | 0 | 0 | 1 | 0 | 0 | 0 | 0 | 0 | 0 | 0 |
| Plagiochila longispina | 1 | 0 | 0 | 0 | 0 | 0 | 0 | 0 | 0 | 0 |
| Plagiochila maderensis* | 0 | 1 | 0 | 0 | 1 | 0 | 0 | 0 | 0 | 0 |
| Plagiochila moenkemeyeri | 0 | 1 | 0 | 0 | 0 | 0 | 0 | 0 | 0 | 0 |
| Plagiochila neckeroidea | 0 | 1 | 0 | 0 | 0 | 0 | 0 | 0 | 0 | 0 |
| Plagiochila papillifolia | 1 | 0 | 0 | 0 | 0 | 0 | 0 | 0 | 0 | 0 |
| Plagiochila pectinata | 0 | 0 | 1 | 0 | 0 | 0 | 0 | 0 | 0 | 0 |
| Plagiochila pinniflora | 0 | 0 | 1 | 0 | 0 | 0 | 0 | 0 | 0 | 0 |
| Plagiochila porelloides | 0 | 1 | 0 | 0 | 1 | 0 | 0 | 0 | 0 | 0 |
| Plagiochila praemorsa | 0 | 0 | 1 | 0 | 0 | 0 | 0 | 0 | 0 | 0 |
| Plagiochila punctata | 1 | 1 | 0 | 0 | 1 | 0 | 0 | 0 | 0 | 0 |
| Plagiochila retrorsa | 0 | 1 | 0 | 0 | 0 | 0 | 0 | 0 | 0 | 0 |
| Plagiochila sarmentosa | 0 | 0 | 1 | 0 | 0 | 0 | 0 | 0 | 0 | 0 |
| Plagiochila spinulosa | 0 | 1 | 0 | 0 | 0 | 0 | 0 | 0 | 0 | 0 |
| Plagiochila stricta | 0 | 1 | 0 | 0 | 1 | 0 | 0 | 0 | 0 | 0 |
| Plagiochila strictifolia | 0 | 0 | 1 | 0 | 0 | 0 | 0 | 0 | 0 | 0 |
| Plagiochila terebrans | 0 | 0 | 1 | 0 | 0 | 0 | 0 | 0 | 0 | 0 |
| Plagiochila tricuspis | 0 | 0 | 1 | 0 | 0 | 0 | 0 | 0 | 0 | 0 |
| Plagiochila tristaniana* | 0 | 0 | 0 | 0 | 0 | 0 | 0 | 0 | 0 | 1 |
| Plagiochila virginica | 0 | 1 | 0 | 0 | 1 | 0 | 0 | 0 | 0 | 0 |
| Plagiochila wacei* | 0 | 0 | 0 | 0 | 0 | 0 | 0 | 0 | 0 | 1 |
| Plagiochila winteri* | 0 | 0 | 1 | 0 | 0 | 0 | 0 | 0 | 0 | 0 |
| Pleurozia gigantea | 0 | 0 | 1 | 1 | 0 | 0 | 0 | 0 | 1 | 0 |
| Plicanthus hirtellus | 0 | 0 | 1 | 0 | 0 | 0 | 0 | 0 | 0 | 0 |
| Porella abyssinica var. hoehnelii | 0 | 0 | 1 | 0 | 0 | 0 | 0 | 0 | 0 | 0 |
| Porella arboris-vita | 0 | 0 | 0 | 1 | 0 | 0 | 0 | 0 | 0 | 0 |
| Porella canariensis* | 1 | 1 | 0 | 1 | 1 | 0 | 0 | 0 | 0 | 0 |
| Porella capensis | 0 | 0 | 1 | 0 | 0 | 0 | 0 | 0 | 0 | 0 |
| Porella chilensis | 0 | 0 | 0 | 0 | 0 | 0 | 0 | 0 | 0 | 1 |
| Porella cordaeana | 0 | 1 | 0 | 0 | 0 | 0 | 0 | 0 | 0 | 0 |
| Porella inaequalis | 0 | 1 | 0 | 0 | 0 | 0 | 0 | 0 | 0 | 0 |
| Porella obtusata | 1 | 1 | 0 | 0 | 1 | 0 | 0 | 0 | 0 | 0 |
| Porella platyphylla | 0 | 0 | 0 | 0 | 1 | 0 | 0 | 0 | 0 | 0 |
| Porella subdentata var. camerunensis | 0 | 0 | 1 | 0 | 0 | 0 | 0 | 0 | 0 | 0 |
| Porella subdentata var. subdentata | 0 | 0 | 1 | 0 | 0 | 0 | 0 | 0 | 0 | 0 |
| Prionolejeunea grata | 0 | 0 | 1 | 0 | 0 | 0 | 0 | 0 | 0 | 0 |
| Prionolejeunea principensis* | 0 | 0 | 1 | 0 | 0 | 0 | 0 | 0 | 0 | 0 |
| Pseudocephalozia quadriloba | 0 | 0 | 0 | 0 | 0 | 0 | 0 | 0 | 0 | 1 |
| Pseudolepicolea temnomoides* | 0 | 0 | 0 | 0 | 0 | 0 | 0 | 0 | 0 | 1 |
| Pseudomarsupidium decipiens | 1 | 1 | 0 | 0 | 0 | 0 | 0 | 0 | 1 | 1 |
| Radula ankefinensis | 0 | 0 | 1 | 0 | 0 | 0 | 0 | 0 | 0 | 0 |
| Radula appressa | 0 | 0 | 1 | 0 | 0 | 0 | 0 | 0 | 0 | 0 |
| Radula aquilegia | 1 | 1 | 0 | 0 | 1 | 0 | 0 | 0 | 0 | 0 |
| Radula carringtonii* | 1 | 1 | 0 | 0 | 1 | 0 | 0 | 0 | 0 | 0 |
| Radula diversifolia | 0 | 0 | 0 | 0 | 0 | 0 | 0 | 0 | 0 | 1 |
| Radula fernandezana | 0 | 0 | 0 | 0 | 0 | 0 | 0 | 0 | 0 | 1 |
| Radula flaccida | 0 | 0 | 1 | 0 | 0 | 0 | 0 | 0 | 0 | 0 |
| Radula fulvifolia | 0 | 0 | 1 | 0 | 0 | 0 | 0 | 0 | 1 | 0 |
| Radula hastata | 0 | 0 | 0 | 0 | 0 | 0 | 0 | 0 | 0 | 1 |
| Radula helix | 0 | 0 | 0 | 0 | 0 | 0 | 0 | 0 | 0 | 1 |
| Radula holtii* | 1 | 1 | 0 | 0 | 1 | 0 | 0 | 0 | 0 | 0 |
| Radula jonesii* | 0 | 1 | 0 | 0 | 1 | 0 | 0 | 0 | 0 | 0 |
| Radula lindenbergiana | 1 | 1 | 0 | 1 | 1 | 0 | 0 | 0 | 0 | 0 |
| Radula nudicaulis | 1 | 1 | 0 | 0 | 0 | 0 | 0 | 0 | 0 | 0 |
| Radula plumosa | 0 | 0 | 0 | 0 | 0 | 0 | 0 | 0 | 0 | 1 |
| Radula quadrata | 0 | 0 | 1 | 0 | 0 | 0 | 0 | 0 | 0 | 0 |
| Radula stenocalyx | 0 | 0 | 1 | 0 | 0 | 0 | 0 | 0 | 0 | 0 |
| Radula striata | 0 | 0 | 0 | 0 | 0 | 0 | 0 | 0 | 0 | 1 |
| Radula wichurae* | 1 | 1 | 0 | 0 | 1 | 0 | 0 | 0 | 0 | 0 |
| Radulina borbonica | 0 | 0 | 1 | 0 | 0 | 0 | 0 | 0 | 0 | 0 |
| Reboulia hemisphaerica | 1 | 1 | 0 | 1 | 1 | 0 | 0 | 0 | 0 | 1 |
| Riccardia alcicornis | 0 | 0 | 0 | 0 | 0 | 0 | 0 | 0 | 0 | 1 |
| Riccardia amazonica | 0 | 0 | 1 | 0 | 0 | 0 | 0 | 1 | 0 | 0 |
| Riccardia autoica | 0 | 0 | 0 | 0 | 0 | 0 | 0 | 0 | 0 | 1 |
| Riccardia chamedryfolia | 1 | 1 | 0 | 0 | 0 | 0 | 0 | 0 | 1 | 0 |
| Riccardia crassicrispa | 0 | 0 | 0 | 0 | 0 | 0 | 0 | 0 | 0 | 1 |
| Riccardia floribunda | 0 | 0 | 0 | 0 | 0 | 0 | 0 | 0 | 0 | 1 |
| Riccardia georgiensis | 0 | 0 | 0 | 0 | 0 | 0 | 0 | 0 | 0 | 1 |
| Riccardia latifrons | 1 | 1 | 0 | 0 | 0 | 0 | 0 | 0 | 0 | 0 |
| Riccardia longispica | 0 | 0 | 1 | 0 | 0 | 0 | 0 | 0 | 0 | 0 |
| Riccardia mejlandii* | 0 | 0 | 0 | 0 | 0 | 0 | 0 | 0 | 0 | 1 |
| Riccardia multifida | 1 | 1 | 0 | 0 | 1 | 0 | 0 | 0 | 0 | 0 |
| Riccardia mycophora | 0 | 0 | 0 | 0 | 0 | 0 | 0 | 0 | 0 | 1 |
| Riccardia pallidevirens | 0 | 0 | 0 | 0 | 0 | 0 | 0 | 0 | 0 | 1 |
| Riccardia palmata | 1 | 1 | 0 | 0 | 0 | 0 | 0 | 0 | 0 | 0 |
| Riccardia patens | 0 | 0 | 0 | 0 | 0 | 0 | 0 | 0 | 0 | 1 |
| Riccardia prehensilis | 0 | 0 | 0 | 0 | 0 | 0 | 0 | 0 | 0 | 1 |
| Riccardia tenerrima | 0 | 0 | 0 | 0 | 0 | 0 | 0 | 0 | 0 | 1 |
| Riccardia tristaniana* | 0 | 0 | 0 | 0 | 0 | 0 | 0 | 0 | 0 | 1 |
| Riccardia sp. | 0 | 0 | 0 | 0 | 0 | 0 | 0 | 1 | 1 | 0 |
| Riccia atlantica* | 0 | 1 | 0 | 0 | 0 | 0 | 0 | 0 | 0 | 0 |
| Riccia atromarginata | 0 | 1 | 0 | 1 | 1 | 0 | 0 | 0 | 0 | 0 |
| Riccia atropurpurea | 0 | 0 | 0 | 0 | 0 | 0 | 0 | 0 | 0 | 1 |
| Riccia bifurca | 1 | 1 | 0 | 0 | 1 | 0 | 0 | 0 | 0 | 0 |
| Riccia boumanii* | 0 | 0 | 0 | 0 | 1 | 0 | 0 | 0 | 0 | 0 |
| Riccia cavernosa | 0 | 1 | 0 | 1 | 1 | 0 | 0 | 1 | 0 | 0 |
| Riccia ciliata | 0 | 1 | 0 | 1 | 1 | 0 | 0 | 0 | 0 | 0 |
| Riccia ciliifera | 0 | 1 | 0 | 0 | 1 | 0 | 0 | 0 | 0 | 0 |
| Riccia congoana | 0 | 0 | 1 | 1 | 0 | 0 | 0 | 0 | 0 | 0 |
| Riccia crinita | 0 | 0 | 0 | 1 | 0 | 0 | 0 | 0 | 0 | 0 |
| Riccia crozalsii | 1 | 1 | 0 | 0 | 1 | 0 | 0 | 0 | 0 | 0 |
| Riccia crystalina | 1 | 1 | 0 | 1 | 1 | 0 | 0 | 0 | 0 | 0 |
| Riccia discolor | 0 | 0 | 1 | 0 | 0 | 0 | 0 | 0 | 0 | 0 |
| Riccia fluitans | 0 | 0 | 1 | 0 | 0 | 0 | 0 | 0 | 0 | 0 |
| Riccia frostii | 0 | 0 | 0 | 1 | 0 | 0 | 0 | 0 | 0 | 0 |
| Riccia glauca | 1 | 1 | 0 | 0 | 1 | 0 | 0 | 0 | 0 | 0 |
| Riccia gougetiana | 0 | 1 | 0 | 1 | 1 | 0 | 0 | 0 | 0 | 0 |
| Riccia huebeneriana | 1 | 0 | 0 | 0 | 0 | 0 | 0 | 0 | 0 | 0 |
| Riccia lamellosa | 0 | 1 | 0 | 0 | 1 | 0 | 0 | 0 | 0 | 0 |
| Riccia lanceolata | 0 | 0 | 1 | 0 | 0 | 0 | 0 | 0 | 0 | 0 |
| Riccia ligula | 1 | 1 | 0 | 0 | 1 | 0 | 0 | 0 | 0 | 0 |
| Riccia macrocarpa | 0 | 1 | 0 | 1 | 1 | 0 | 0 | 0 | 0 | 0 |
| Riccia membranacea | 0 | 0 | 0 | 0 | 0 | 1 | 0 | 0 | 0 | 0 |
| Riccia moenkemeyeri | 0 | 0 | 1 | 0 | 0 | 0 | 0 | 0 | 0 | 0 |
| Riccia nigrella | 1 | 1 | 0 | 1 | 1 | 0 | 0 | 0 | 0 | 0 |
| Riccia papillosa | 0 | 0 | 0 | 0 | 1 | 0 | 0 | 0 | 0 | 0 |
| Riccia ridleyi | 0 | 0 | 0 | 0 | 0 | 0 | 1 | 0 | 0 | 0 |
| Riccia sorocarpa | 1 | 1 | 0 | 1 | 1 | 0 | 0 | 0 | 0 | 0 |
| Riccia sorocarpa var. heegii | 0 | 0 | 0 | 1 | 0 | 0 | 0 | 0 | 0 | 0 |
| Riccia stricta | 0 | 0 | 1 | 0 | 0 | 0 | 0 | 0 | 0 | 0 |
| Riccia subbifurca | 0 | 1 | 0 | 0 | 1 | 0 | 0 | 0 | 0 | 0 |
| Riccia trabutiana | 1 | 1 | 0 | 1 | 1 | 0 | 0 | 0 | 0 | 0 |
| Riccia warnstorfii | 1 | 1 | 0 | 0 | 0 | 0 | 0 | 0 | 0 | 0 |
| Riccia sp. | 0 | 1 | 0 | 0 | 0 | 0 | 0 | 0 | 0 | 0 |
| Riella affinis | 0 | 0 | 0 | 0 | 1 | 0 | 0 | 0 | 0 | 0 |
| Riella cossoniana | 1 | 0 | 0 | 0 | 1 | 0 | 0 | 0 | 0 | 0 |
| Riella echinata | 0 | 0 | 0 | 0 | 1 | 0 | 0 | 0 | 0 | 0 |
| Riella notarisii | 0 | 0 | 0 | 0 | 1 | 0 | 0 | 0 | 0 | 0 |
| Saccogyna viticulosa | 1 | 1 | 0 | 0 | 1 | 0 | 0 | 0 | 0 | 0 |
| Scapania compacta | 1 | 1 | 0 | 0 | 1 | 0 | 0 | 0 | 0 | 0 |
| Scapania curta | 1 | 1 | 0 | 0 | 1 | 0 | 0 | 0 | 0 | 0 |
| Scapania gracilis | 1 | 1 | 0 | 0 | 1 | 0 | 0 | 0 | 0 | 0 |
| Scapania nemorea | 1 | 1 | 0 | 0 | 1 | 0 | 0 | 0 | 0 | 0 |
| Scapania scandica | 1 | 0 | 0 | 0 | 0 | 0 | 0 | 0 | 0 | 0 |
| Scapania subalpina | 0 | 1 | 0 | 0 | 0 | 0 | 0 | 0 | 0 | 0 |
| Scapania umbrosa | 0 | 1 | 0 | 0 | 0 | 0 | 0 | 0 | 0 | 0 |
| Scapania undulata | 1 | 1 | 0 | 0 | 1 | 0 | 0 | 0 | 0 | 0 |
| Schiffneriolejeunea occulta | 0 | 0 | 1 | 0 | 0 | 0 | 0 | 0 | 0 | 0 |
| Schiffneriolejeunea pappeana var. pappeana | 0 | 0 | 1 | 0 | 0 | 0 | 0 | 0 | 0 | 0 |
| Schiffneriolejeunea polycarpa | 0 | 0 | 1 | 0 | 0 | 1 | 0 | 0 | 0 | 0 |
| Schistochila alata | 0 | 0 | 0 | 0 | 0 | 0 | 0 | 0 | 0 | 1 |
| Schistochila pachyphylla* | 0 | 0 | 0 | 0 | 0 | 0 | 0 | 0 | 0 | 1 |
| Schistochila splachnophylla | 0 | 0 | 0 | 0 | 0 | 0 | 0 | 0 | 0 | 1 |
| Schistochilopsis incisa | 1 | 0 | 0 | 0 | 0 | 0 | 0 | 0 | 0 | 0 |
| Solenostoma amoenum | 0 | 0 | 0 | 0 | 0 | 1 | 0 | 0 | 0 | 0 |
| Solenostoma callithrix | 1 | 1 | 0 | 0 | 1 | 0 | 0 | 0 | 0 | 0 |
| Solenostoma crassulum | 0 | 0 | 0 | 0 | 0 | 0 | 0 | 0 | 0 | 1 |
| Solenostoma dusenii | 0 | 0 | 1 | 0 | 0 | 0 | 0 | 0 | 0 | 0 |
| Solenostoma gracillimum | 1 | 1 | 0 | 0 | 1 | 0 | 0 | 0 | 0 | 0 |
| Solenostoma hyalinum | 1 | 1 | 0 | 0 | 1 | 0 | 0 | 0 | 0 | 0 |
| Solenostoma stoloniferum | 0 | 0 | 1 | 0 | 0 | 0 | 0 | 0 | 0 | 0 |
| Southbya nigrella | 0 | 1 | 0 | 0 | 1 | 0 | 0 | 0 | 0 | 0 |
| Southbya tophacea | 1 | 1 | 0 | 0 | 1 | 0 | 0 | 0 | 0 | 0 |
| Sphaerocarpos europaeus | 1 | 0 | 0 | 0 | 1 | 0 | 0 | 0 | 0 | 0 |
| Sphenolobopsis pearsonii | 0 | 0 | 0 | 0 | 0 | 0 | 0 | 0 | 0 | 1 |
| Sphenolobus minutus | 1 | 1 | 0 | 0 | 0 | 0 | 0 | 0 | 0 | 0 |
| Spruceanthus abbreviatus | 0 | 0 | 1 | 0 | 0 | 0 | 0 | 0 | 0 | 0 |
| Spruceanthus floreus | 0 | 0 | 1 | 0 | 0 | 0 | 0 | 0 | 0 | 0 |
| Stictolejeunea balfourii | 0 | 0 | 1 | 0 | 0 | 0 | 0 | 0 | 0 | 0 |
| Symbiezidium barbiflorum | 0 | 0 | 0 | 0 | 0 | 1 | 0 | 0 | 0 | 0 |
| Symphyogyna brasiliensis | 0 | 0 | 0 | 0 | 0 | 0 | 0 | 1 | 1 | 1 |
| Symphyogyna circinata | 0 | 0 | 0 | 0 | 0 | 0 | 0 | 0 | 0 | 1 |
| Symphyogyna hochstetteri | 0 | 0 | 0 | 0 | 0 | 0 | 0 | 0 | 0 | 1 |
| Symphyogyna podophylla | 0 | 0 | 0 | 0 | 0 | 0 | 0 | 0 | 0 | 1 |
| Syzygiella colorata | 0 | 0 | 0 | 0 | 0 | 0 | 0 | 0 | 0 | 1 |
| Syzygiella colorata var. collenchymata* | 0 | 0 | 0 | 0 | 0 | 0 | 0 | 0 | 0 | 1 |
| Syzygiella concreta | 0 | 0 | 0 | 0 | 0 | 0 | 0 | 0 | 0 | 1 |
| Syzygiella manca | 0 | 0 | 1 | 1 | 0 | 0 | 0 | 0 | 0 | 0 |
| Syzygiella oenops | 0 | 0 | 0 | 0 | 0 | 0 | 0 | 0 | 0 | 1 |
| Syzygiella paludosa | 0 | 0 | 0 | 0 | 0 | 0 | 0 | 0 | 0 | 1 |
| Syzygiella rubricaulis | 1 | 0 | 0 | 0 | 0 | 0 | 0 | 0 | 0 | 0 |
| Syzygiella securifolia | 0 | 0 | 1 | 0 | 0 | 0 | 0 | 0 | 0 | 0 |
| Syzygiella sonderi | 0 | 0 | 0 | 0 | 0 | 0 | 0 | 0 | 0 | 1 |
| Syzygiella teres | 0 | 0 | 0 | 0 | 0 | 0 | 0 | 0 | 0 | 1 |
| Targionia hypophylla | 1 | 1 | 0 | 1 | 1 | 0 | 0 | 0 | 0 | 0 |
| Targionia lorbeeriana | 1 | 1 | 0 | 1 | 1 | 0 | 0 | 0 | 0 | 0 |
| Telaranea azorica* | 1 | 0 | 0 | 0 | 1 | 0 | 0 | 0 | 0 | 0 |
| Telaranea breviseta | 0 | 0 | 0 | 0 | 0 | 0 | 0 | 0 | 0 | 1 |
| Telaranea coactilis | 0 | 0 | 1 | 0 | 0 | 0 | 0 | 0 | 0 | 0 |
| Telaranea diacantha | 0 | 0 | 1 | 0 | 0 | 0 | 0 | 0 | 0 | 0 |
| Telaranea europaea* | 1 | 1 | 0 | 0 | 1 | 0 | 0 | 0 | 0 | 0 |
| Telaranea nematodes | 0 | 0 | 1 | 0 | 0 | 0 | 0 | 0 | 0 | 0 |
| Telaranea oligophylla | 0 | 0 | 0 | 0 | 0 | 0 | 0 | 0 | 0 | 1 |
| Telaranea sejuncta | 0 | 0 | 0 | 0 | 0 | 0 | 0 | 0 | 0 | 1 |
| Temnoma quadripartitum | 0 | 0 | 0 | 0 | 0 | 0 | 0 | 0 | 0 | 1 |
| Thysananthus auriculatus | 0 | 0 | 1 | 0 | 0 | 0 | 0 | 0 | 0 | 0 |
| Thysananthus niger | 0 | 0 | 1 | 0 | 0 | 0 | 0 | 0 | 0 | 0 |
| Thysananthus turgidus | 0 | 0 | 1 | 0 | 0 | 0 | 0 | 0 | 0 | 0 |
| Triandrophyllum subtrifidum | 0 | 0 | 0 | 0 | 0 | 0 | 0 | 0 | 0 | 1 |
| Trichocolea tomentella | 1 | 0 | 0 | 0 | 0 | 0 | 0 | 0 | 0 | 0 |
| Trilophozia quinquedentata | 0 | 1 | 0 | 0 | 0 | 0 | 0 | 0 | 0 | 0 |
| Tritomaria exsecta | 0 | 1 | 0 | 0 | 0 | 0 | 0 | 0 | 0 | 0 |
| Tylimanthus anisodontus* | 0 | 0 | 0 | 0 | 0 | 0 | 0 | 0 | 1 | 0 |
| Zantenia borneensis | 0 | 0 | 0 | 0 | 0 | 0 | 0 | 0 | 0 | 1 |
| Zantenia denticulata | 0 | 0 | 0 | 0 | 0 | 0 | 0 | 0 | 0 | 1 |
| Zantenia karstenii | 0 | 0 | 0 | 0 | 0 | 0 | 0 | 0 | 0 | 1 |
| Zantenia prionophylla | 0 | 0 | 0 | 0 | 0 | 0 | 0 | 0 | 0 | 1 |
|  |  |  |  |  |  |  |  |  |  |  |
