# Supplementary material for: Atlantic oceanic islands and archipelagos: Physical structures, plant diversity, and affinities of the bryofloras
Source: Biodivers Data J. 2025 Feb 28;13:e141577. doi: 10.3897/BDJ.13.e141577 (PMC11889432; doi:10.3897/BDJ.13.e141577)
Supplement: Supplementary material 2 — Table S2. Matrix of presence and absence of moss taxa [file bdj-13-e141577-s002.docx]

**Supplemental Data**

**Table S2**. Matrix of presence and absence of moss taxa on the ten islands studied. *= Endemic

| TAXA / ISLANDS OR ARCHIPELAGOS | AZO | MAD | STP | CVE | CAN | TRI | FN | ASC | STH | TRC |
| --- | --- | --- | --- | --- | --- | --- | --- | --- | --- | --- |
| Acaulon mediterraneum | 0 | 0 | 0 | 0 | 1 | 0 | 0 | 0 | 0 | 0 |
| Acaulon muticum | 0 | 1 | 0 | 0 | 1 | 0 | 0 | 0 | 0 | 0 |
| Acaulon triquetrum | 0 | 1 | 0 | 0 | 1 | 0 | 0 | 0 | 0 | 0 |
| Acroporium megasporum | 0 | 0 | 0 | 0 | 0 | 0 | 0 | 1 | 0 | 0 |
| Alleniella complanata* | 1 | 1 | 0 | 0 | 1 | 0 | 0 | 0 | 0 | 0 |
| Aloina aloides | 0 | 1 | 0 | 0 | 1 | 0 | 0 | 0 | 0 | 0 |
| Aloina ambigua | 1 | 1 | 0 | 1 | 1 | 0 | 0 | 0 | 0 | 0 |
| Aloina brevirostris | 0 | 0 | 0 | 0 | 1 | 0 | 0 | 0 | 0 | 0 |
| Aloina humilis* | 0 | 0 | 0 | 0 | 1 | 0 | 0 | 0 | 0 | 0 |
| Aloina rigida | 1 | 1 | 0 | 1 | 1 | 0 | 0 | 0 | 0 | 0 |
| Amblystegium patentiflexuosum* | 0 | 0 | 0 | 0 | 0 | 0 | 0 | 0 | 0 | 1 |
| Amblystegium serpens | 1 | 0 | 0 | 0 | 1 | 0 | 0 | 0 | 0 | 0 |
| Amblystegium strictoserpens* | 0 | 0 | 0 | 0 | 0 | 0 | 0 | 0 | 0 | 1 |
| Amphidium curvipes* | 0 | 1 | 0 | 0 | 1 | 0 | 0 | 0 | 0 | 0 |
| Amphidium lapponicum | 0 | 0 | 0 | 0 | 1 | 0 | 0 | 0 | 0 | 0 |
| Amphidium mougeotii | 1 | 1 | 0 | 0 | 1 | 0 | 0 | 0 | 0 | 0 |
| Andoa berthelotiana* | 1 | 1 | 0 | 0 | 1 | 0 | 0 | 0 | 0 | 0 |
| Andreaea alpestris* | 0 | 1 | 0 | 0 | 0 | 0 | 0 | 0 | 0 | 0 |
| Andreaea aquatica | 0 | 0 | 0 | 0 | 0 | 0 | 0 | 0 | 0 | 1 |
| Andreaea atlantica* | 0 | 0 | 0 | 0 | 0 | 0 | 0 | 0 | 0 | 1 |
| Andreaea crassinervia | 0 | 0 | 0 | 0 | 1 | 0 | 0 | 0 | 0 | 0 |
| Andreaea flexuosa subsp. luisieri* | 0 | 1 | 0 | 0 | 0 | 0 | 0 | 0 | 0 | 0 |
| Andreaea grimmioides | 0 | 0 | 0 | 0 | 0 | 0 | 0 | 0 | 0 | 1 |
| Andreaea heinemannii | 0 | 1 | 0 | 0 | 1 | 0 | 0 | 0 | 0 | 0 |
| Andreaea heinemannii subsp. crassifolia | 0 | 1 | 0 | 0 | 1 | 0 | 0 | 0 | 0 | 0 |
| Andreaea parallela | 0 | 0 | 0 | 0 | 0 | 0 | 0 | 0 | 0 | 1 |
| Andreaea regularis | 0 | 0 | 0 | 0 | 0 | 0 | 0 | 0 | 0 | 1 |
| Andreaea rothii | 0 | 1 | 0 | 0 | 0 | 0 | 0 | 0 | 0 | 0 |
| Andreaea rupestris | 1 | 1 | 0 | 0 | 0 | 0 | 0 | 0 | 0 | 1 |
| Andreaea squarrifolia* | 0 | 0 | 0 | 0 | 0 | 0 | 0 | 0 | 0 | 1 |
| Anoectangium aestivum | 1 | 1 | 1 | 1 | 1 | 0 | 0 | 0 | 0 | 0 |
| Anoectangium angustifolium* | 1 | 1 | 0 | 0 | 1 | 0 | 0 | 0 | 0 | 0 |
| Anoectangium stracheyanum | 0 | 0 | 1 | 0 | 0 | 0 | 0 | 0 | 0 | 0 |
| Anomobyum apiculatum | 0 | 0 | 1 | 1 | 1 | 0 | 1 | 0 | 0 | 0 |
| Anomobryum julaceum | 1 | 1 | 0 | 1 | 1 | 0 | 0 | 1 | 0 | 1 |
| Anomobryum notarisii | 1 | 1 | 1 | 1 | 1 | 0 | 0 | 0 | 0 | 0 |
| Anomodon viticulosus | 0 | 0 | 0 | 0 | 1 | 0 | 0 | 0 | 0 | 0 |
| Antitrichia californica | 0 | 1 | 0 | 0 | 1 | 0 | 0 | 0 | 0 | 0 |
| Antitrichia curtipendula | 0 | 1 | 0 | 0 | 1 | 0 | 0 | 0 | 0 | 0 |
| Archidium alternifolium | 1 | 1 | 0 | 0 | 1 | 0 | 0 | 0 | 0 | 0 |
| Atlantichella calvescens* | 1 | 1 | 0 | 0 | 1 | 0 | 0 | 0 | 0 | 0 |
| Atrichopsis tristaniensis* | 0 | 0 | 0 | 0 | 0 | 0 | 0 | 0 | 0 | 1 |
| Atrichum androgynum | 1 | 0 | 0 | 0 | 0 | 0 | 0 | 0 | 0 | 0 |
| Atrichum angustatum | 1 | 1 | 0 | 0 | 1 | 0 | 0 | 0 | 0 | 0 |
| Atrichum tenellum | 1 | 0 | 0 | 0 | 0 | 0 | 0 | 0 | 0 | 0 |
| Atrichum undulatum | 1 | 1 | 0 | 0 | 1 | 0 | 0 | 0 | 0 | 0 |
| Aulacomnium androgynum | 0 | 0 | 0 | 0 | 1 | 0 | 0 | 0 | 0 | 0 |
| Aulacomnium palustre | 1 | 0 | 0 | 0 | 0 | 0 | 0 | 0 | 0 | 0 |
| Austroleptodontium interruptum | 0 | 0 | 0 | 0 | 0 | 0 | 0 | 0 | 0 | 1 |
| Barbula arcuata | 0 | 0 | 0 | 1 | 0 | 0 | 0 | 0 | 0 | 0 |
| Barbula convoluta | 0 | 0 | 0 | 1 | 0 | 0 | 0 | 0 | 0 | 0 |
| Barbula indica | 0 | 0 | 0 | 0 | 0 | 1 | 0 | 0 | 0 | 0 |
| Barbula seramensis | 0 | 0 | 1 | 0 | 0 | 0 | 0 | 0 | 0 | 0 |
| Barbula unguiculata | 1 | 1 | 0 | 1 | 1 | 0 | 0 | 0 | 0 | 0 |
| Bartramia aprica | 0 | 0 | 0 | 1 | 0 | 0 | 0 | 0 | 0 | 0 |
| Bartramia ithyphylla subsp. patens | 0 | 0 | 0 | 0 | 0 | 0 | 0 | 0 | 0 | 1 |
| Bartramia obscura* | 0 | 0 | 0 | 0 | 0 | 0 | 0 | 0 | 0 | 1 |
| Bartramia robusta | 0 | 0 | 0 | 0 | 0 | 0 | 0 | 0 | 0 | 1 |
| Bellibarbula recurva | 0 | 0 | 0 | 0 | 0 | 0 | 1 | 0 | 0 | 0 |
| Blindia acuta | 1 | 1 | 0 | 0 | 0 | 0 | 0 | 0 | 0 | 0 |
| Blindia brachystegia* | 0 | 0 | 0 | 0 | 0 | 0 | 0 | 0 | 0 | 1 |
| Blindia magellanica | 0 | 0 | 0 | 0 | 0 | 0 | 0 | 0 | 0 | 1 |
| Brachymenium acuminatum | 0 | 0 | 0 | 1 | 0 | 0 | 0 | 0 | 0 | 0 |
| Brachymenium exile | 0 | 0 | 0 | 1 | 0 | 0 | 0 | 0 | 0 | 0 |
| Brachymenium flacidifolium | 0 | 0 | 0 | 0 | 0 | 0 | 0 | 0 | 0 | 1 |
| Brachymenium leptophyllum | 0 | 0 | 1 | 0 | 0 | 0 | 0 | 0 | 0 | 0 |
| Brachymenium nepalense | 0 | 0 | 1 | 0 | 0 | 0 | 0 | 0 | 0 | 0 |
| Brachymenium subuliferum | 0 | 0 | 1 | 0 | 0 | 0 | 0 | 0 | 0 | 0 |
| Brachymitrion moritzianum | 0 | 0 | 1 | 0 | 0 | 0 | 0 | 0 | 0 | 0 |
| Brachytheciastrum dieckii | 0 | 0 | 0 | 0 | 1 | 0 | 0 | 0 | 0 | 0 |
| Brachytheciastrum velutinum | 1 | 1 | 0 | 0 | 1 | 0 | 0 | 0 | 0 | 0 |
| Brachythecium albicans | 1 | 1 | 0 | 0 | 1 | 0 | 0 | 0 | 0 | 0 |
| Brachythecium austroglareosum | 0 | 0 | 0 | 0 | 0 | 0 | 0 | 0 | 0 | 1 |
| Brachythecium mildeanum | 1 | 1 | 0 | 0 | 0 | 0 | 0 | 0 | 0 | 0 |
| Brachythecium percurrens* | 0 | 1 | 0 | 0 | 0 | 0 | 0 | 0 | 0 | 0 |
| Brachythecium rivulare | 1 | 1 | 0 | 0 | 1 | 0 | 0 | 0 | 0 | 0 |
| Brachythecium rutabulum | 1 | 1 | 0 | 0 | 1 | 0 | 0 | 0 | 0 | 0 |
| Brachythecium rutabulum var. atlanticum* | 1 | 1 | 0 | 0 | 0 | 0 | 0 | 0 | 0 | 0 |
| Brachythecium salebrosum | 1 | 0 | 0 | 0 | 1 | 0 | 0 | 0 | 0 | 0 |
| Brachythecium subpilosum | 0 | 0 | 0 | 0 | 0 | 0 | 0 | 0 | 0 | 1 |
| Brachythecium subplicatum | 0 | 0 | 0 | 0 | 0 | 0 | 0 | 0 | 0 | 1 |
| Brachythecium sp. | 0 | 0 | 0 | 0 | 0 | 0 | 0 | 1 | 0 | 0 |
| Braunia alopecura | 0 | 0 | 0 | 1 | 0 | 0 | 0 | 0 | 0 | 0 |
| Breutelia dumosa | 0 | 0 | 0 | 0 | 0 | 0 | 0 | 0 | 0 | 1 |
| Breutelia integrifolia | 0 | 0 | 0 | 0 | 0 | 0 | 0 | 0 | 0 | 1 |
| Breutelia popinqua | 0 | 0 | 0 | 0 | 0 | 0 | 0 | 0 | 0 | 1 |
| Bryoceuthospora aethiopica | 0 | 0 | 0 | 1 | 0 | 0 | 0 | 0 | 0 | 0 |
| Bryoerythrophyllum campylocarpum | 0 | 1 | 0 | 1 | 0 | 0 | 0 | 0 | 0 | 0 |
| Bryoerythrophyllum ferruginascens | 0 | 0 | 0 | 1 | 0 | 0 | 0 | 0 | 0 | 0 |
| Bryoerythrophyllum inaequalifolium | 1 | 1 | 0 | 1 | 1 | 0 | 0 | 0 | 0 | 0 |
| Bryoerythrophyllum recurvirostrum | 0 | 1 | 0 | 0 | 1 | 0 | 0 | 0 | 0 | 0 |
| Bryoxiphium madeirense* | 0 | 1 | 0 | 0 | 0 | 0 | 0 | 0 | 0 | 0 |
| Bryoxiphium norvegicum | 1 | 0 | 0 | 0 | 0 | 0 | 0 | 0 | 0 | 0 |
| Bryum “A” | 0 | 0 | 0 | 0 | 0 | 0 | 0 | 1 | 0 | 0 |
| Bryum “C” | 0 | 0 | 0 | 0 | 0 | 0 | 0 | 0 | 0 | 1 |
| Bryum anomodon | 0 | 0 | 0 | 1 | 0 | 0 | 0 | 0 | 0 | 0 |
| Bryum arachnoideum | 0 | 0 | 0 | 0 | 0 | 1 | 0 | 1 | 0 | 0 |
| Bryum argenteum | 1 | 1 | 1 | 1 | 1 | 1 | 0 | 0 | 1 | 0 |
| Bryum atenense | 0 | 0 | 0 | 0 | 0 | 0 | 1 | 0 | 0 | 0 |
| Bryum canariense | 1 | 1 | 0 | 1 | 1 | 0 | 0 | 0 | 1 | 0 |
| Bryum coronatum | 0 | 0 | 1 | 0 | 0 | 0 | 1 | 1 | 0 | 0 |
| Bryum dichotomum | 1 | 1 | 0 | 1 | 1 | 0 | 0 | 1 | 1 | 0 |
| Bryum gemmiferum | 0 | 0 | 0 | 0 | 1 | 0 | 0 | 0 | 0 | 0 |
| Bryum gemmilucens | 0 | 0 | 0 | 0 | 1 | 0 | 0 | 0 | 0 | 0 |
| Bryum gemniparum | 1 | 1 | 0 | 0 | 1 | 0 | 0 | 0 | 0 | 0 |
| Bryum huillense | 0 | 0 | 1 | 0 | 0 | 0 | 0 | 0 | 0 | 0 |
| Bryum kikuyense | 0 | 0 | 0 | 1 | 0 | 0 | 0 | 0 | 0 | 0 |
| Bryum klinggraeffii | 0 | 1 | 0 | 1 | 0 | 0 | 0 | 0 | 0 | 0 |
| Bryum laevigatum | 0 | 0 | 0 | 0 | 0 | 0 | 0 | 0 | 0 | 1 |
| Bryum megalacrion | 0 | 0 | 0 | 0 | 0 | 0 | 0 | 0 | 0 | 1 |
| Bryum orbiculatifolium | 0 | 0 | 0 | 0 | 0 | 0 | 0 | 0 | 0 | 1 |
| Bryum pseudotriquetrum | 0 | 0 | 0 | 0 | 0 | 0 | 0 | 0 | 0 | 1 |
| Bryum radiculosum | 1 | 1 | 0 | 1 | 1 | 0 | 0 | 0 | 0 | 0 |
| Bryum rubens | 0 | 0 | 0 | 0 | 0 | 0 | 0 | 0 | 1 | 0 |
| Bryum rubicundum | 0 | 0 | 0 | 1 | 0 | 0 | 0 | 0 | 0 | 0 |
| Bryum rubrocostatum* | 0 | 0 | 0 | 0 | 0 | 0 | 0 | 1 | 0 | 0 |
| Bryum ruderale | 1 | 1 | 0 | 1 | 1 | 0 | 0 | 0 | 0 | 0 |
| Bryum sauteri | 1 | 1 | 0 | 1 | 1 | 0 | 0 | 1 | 1 | 0 |
| Bryum subapiculatum | 0 | 0 | 0 | 0 | 0 | 0 | 1 | 0 | 1 | 0 |
| Bryum subulinerve* | 0 | 0 | 0 | 0 | 0 | 0 | 0 | 0 | 0 | 1 |
| Bryum thomeanum* | 0 | 0 | 1 | 0 | 0 | 0 | 0 | 0 | 0 | 0 |
| Bryum tristaniense* | 0 | 0 | 0 | 0 | 0 | 0 | 0 | 0 | 0 | 1 |
| Bryum valparaisense | 0 | 0 | 0 | 0 | 1 | 0 | 0 | 0 | 0 | 0 |
| Bryum violaceum | 0 | 0 | 0 | 0 | 1 | 0 | 0 | 0 | 0 | 0 |
| Bryum sp. | 0 | 0 | 0 | 0 | 0 | 1 | 0 | 0 | 0 | 0 |
| Bucklandiella subsecunda | 0 | 0 | 0 | 0 | 0 | 0 | 0 | 0 | 0 | 1 |
| Caduciella mariei | 0 | 0 | 1 | 0 | 0 | 0 | 0 | 0 | 0 | 0 |
| Callicladium imponens | 1 | 0 | 0 | 0 | 0 | 0 | 0 | 0 | 0 | 0 |
| Callicostella brevipes | 0 | 0 | 1 | 0 | 0 | 0 | 0 | 0 | 0 | 0 |
| Callicostella chionophylla* | 0 | 0 | 1 | 0 | 0 | 0 | 0 | 0 | 0 | 0 |
| Callicostella fissidentella | 0 | 0 | 1 | 0 | 0 | 0 | 0 | 0 | 0 | 0 |
| Callicostella perpapillata | 0 | 0 | 1 | 0 | 0 | 0 | 0 | 0 | 0 | 0 |
| Callicostella salaziae | 0 | 0 | 1 | 0 | 0 | 0 | 0 | 0 | 0 | 0 |
| Calliergon acuminatum | 0 | 0 | 0 | 0 | 0 | 0 | 0 | 0 | 0 | 1 |
| Calliergonella cuspidata | 1 | 1 | 0 | 0 | 1 | 0 | 0 | 0 | 0 | 0 |
| Calymperes afzelii | 0 | 1 | 0 | 0 | 0 | 0 | 0 | 1 | 0 | 0 |
| Calymperes erosum | 0 | 1 | 0 | 0 | 0 | 0 | 0 | 1 | 0 | 0 |
| Calymperes gaudichaudii | 0 | 0 | 0 | 0 | 0 | 0 | 0 | 0 | 1 | 0 |
| Calymperes lonchophyllum subsp. microblastum | 0 | 1 | 0 | 0 | 0 | 0 | 0 | 0 | 0 | 0 |
| Calymperes lonchophyllum subsp. saxatile | 0 | 1 | 0 | 0 | 0 | 0 | 0 | 0 | 0 | 0 |
| Calymperes palisotii | 0 | 1 | 0 | 0 | 0 | 0 | 1 | 0 | 0 | 0 |
| Calymperes pallidum | 0 | 0 | 0 | 0 | 0 | 0 | 0 | 1 | 0 | 0 |
| Calymperes tenerum | 0 | 1 | 0 | 0 | 0 | 1 | 0 | 0 | 0 | 0 |
| Calymperes thomeanum* | 0 | 1 | 0 | 0 | 0 | 0 | 0 | 0 | 0 | 0 |
| Calyptothecium acutifolium var. breviusculum | 0 | 0 | 1 | 0 | 0 | 0 | 0 | 0 | 0 | 0 |
| Campylopus arcuatus | 0 | 0 | 0 | 0 | 0 | 0 | 0 | 1 | 1 | 1 |
| Campylopus arctocarpus subsp. madecassus | 0 | 0 | 0 | 0 | 0 | 0 | 0 | 0 | 0 | 1 |
| Campylopus atrovirens | 1 | 0 | 0 | 0 | 0 | 0 | 0 | 0 | 0 | 0 |
| Campylopus aureonitens | 0 | 0 | 0 | 0 | 0 | 0 | 0 | 1 | 0 | 0 |
| Campylopus brevipilus | 1 | 1 | 0 | 0 | 0 | 0 | 0 | 0 | 0 | 0 |
| Campylopus carolinae | 0 | 0 | 0 | 0 | 0 | 0 | 0 | 1 | 0 | 0 |
| Campylopus cygneus | 1 | 1 | 0 | 0 | 0 | 0 | 0 | 0 | 0 | 0 |
| Campylopus dicranoides | 0 | 0 | 0 | 0 | 0 | 0 | 0 | 1 | 0 | 0 |
| Campylopus flaccidus | 1 | 0 | 0 | 0 | 0 | 0 | 0 | 0 | 0 | 0 |
| Campylopus flexuosus | 1 | 1 | 1 | 0 | 0 | 0 | 0 | 0 | 1 | 0 |
| Campylopus fragilis | 1 | 1 | 0 | 0 | 1 | 1 | 0 | 0 | 0 | 0 |
| Campylopus incrassatus | 1 | 1 | 0 | 0 | 1 | 0 | 0 | 1 | 0 | 1 |
| Campylopus introflexus | 1 | 1 | 0 | 0 | 1 | 1 | 0 | 1 | 1 | 1 |
| Campylopus pilifer | 1 | 1 | 0 | 1 | 1 | 0 | 0 | 1 | 1 | 0 |
| Campylopus pyrifomis | 1 | 1 | 0 | 1 | 0 | 0 | 0 | 1 | 0 | 1 |
| Campylopus savannarum | 0 | 0 | 1 | 0 | 0 | 0 | 0 | 0 | 0 | 0 |
| Campylopus shawii | 1 | 0 | 0 | 0 | 0 | 0 | 0 | 0 | 0 | 0 |
| Campylopus subulatus | 1 | 1 | 0 | 0 | 0 | 0 | 0 | 0 | 0 | 0 |
| Campylopus vesticaulis | 0 | 0 | 0 | 0 | 0 | 0 | 0 | 0 | 0 | 1 |
| Campylostelium pitardii | 0 | 0 | 0 | 0 | 1 | 0 | 0 | 0 | 0 | 0 |
| Campylostelium strictum* | 0 | 0 | 0 | 0 | 1 | 0 | 0 | 0 | 0 | 0 |
| Catagonium nitidum | 0 | 0 | 0 | 0 | 0 | 0 | 0 | 0 | 0 | 1 |
| Ceratodon conicus | 0 | 0 | 1 | 0 | 0 | 0 | 0 | 0 | 0 | 0 |
| Ceratodon purpureus subsp. convolutus | 1 | 1 | 0 | 1 | 1 | 1 | 0 | 0 | 0 | 0 |
| Ceratodon purpureus subsp. purpureus | 1 | 1 | 0 | 0 | 1 | 0 | 0 | 0 | 0 | 1 |
| Ceratodon purpureus subsp. stenocarpus | 1 | 1 | 0 | 0 | 1 | 0 | 0 | 0 | 0 | 0 |
| Chenia leptophylla | 1 | 1 | 0 | 1 | 1 | 0 | 0 | 1 | 1 | 0 |
| Chionoloma angustatum | 0 | 0 | 0 | 0 | 0 | 0 | 0 | 1 | 0 | 0 |
| Chionoloma bombayense | 0 | 1 | 0 | 0 | 0 | 0 | 0 | 1 | 1 | 0 |
| Chionoloma tenuirostre | 0 | 1 | 0 | 1 | 0 | 0 | 0 | 0 | 0 | 0 |
| Chrysoblastella chilensis | 0 | 0 | 0 | 0 | 0 | 0 | 0 | 0 | 0 | 1 |
| Cinclidotus fontinaloides | 0 | 1 | 0 | 0 | 1 | 0 | 0 | 0 | 0 | 0 |
| Cirriphyllum crassinervium* | 0 | 1 | 0 | 0 | 1 | 0 | 0 | 0 | 0 | 0 |
| Cirriphyllum piliferum | 1 | 0 | 0 | 0 | 0 | 0 | 0 | 0 | 0 | 0 |
| Coscinodon cribrosus | 0 | 0 | 0 | 0 | 1 | 0 | 0 | 0 | 0 | 0 |
| Cratoneuron filicinum | 0 | 1 | 0 | 0 | 1 | 0 | 0 | 0 | 0 | 0 |
| Cratoneuron sp. | 0 | 0 | 0 | 0 | 0 | 0 | 0 | 0 | 0 | 1 |
| Crossidium aberrans | 0 | 0 | 0 | 0 | 1 | 0 | 0 | 0 | 0 | 0 |
| Crossidium crassinervium | 0 | 1 | 0 | 1 | 1 | 0 | 0 | 0 | 0 | 0 |
| Crossidium davidai | 0 | 0 | 0 | 0 | 1 | 0 | 0 | 0 | 0 | 0 |
| Crossidium geheebii | 0 | 0 | 0 | 1 | 1 | 0 | 0 | 0 | 0 | 0 |
| Crossidium squamiferum | 0 | 1 | 0 | 1 | 1 | 0 | 0 | 0 | 0 | 0 |
| Cryphaea heteromalla | 1 | 0 | 0 | 1 | 1 | 0 | 0 | 0 | 0 | 0 |
| Cryptoleptodon longisetus | 0 | 0 | 0 | 1 | 0 | 0 | 0 | 0 | 0 | 0 |
| Ctenidium molluscum | 1 | 0 | 0 | 0 | 1 | 0 | 0 | 0 | 0 | 0 |
| Cyclodictyon filicuspis | 0 | 0 | 1 | 0 | 0 | 0 | 0 | 0 | 0 | 0 |
| Cyclodictyon laetevirens | 1 | 1 | 1 | 0 | 1 | 0 | 0 | 0 | 0 | 1 |
| Cynodontium bruntonii | 0 | 0 | 0 | 0 | 1 | 0 | 0 | 0 | 0 | 0 |
| Daltonia lindigiana | 1 | 0 | 0 | 0 | 0 | 0 | 0 | 0 | 0 | 0 |
| Daltonia splachnoides | 0 | 1 | 0 | 0 | 0 | 0 | 0 | 0 | 1 | 0 |
| Daltonia tristaniensis* | 0 | 0 | 0 | 0 | 0 | 0 | 0 | 0 | 0 | 1 |
| Deslooveria quintasii | 0 | 0 | 1 | 0 | 0 | 0 | 0 | 0 | 0 | 0 |
| Deslooveria saotomensis* | 0 | 0 | 1 | 0 | 0 | 0 | 0 | 0 | 0 | 0 |
| Dialytrichia mucronata | 1 | 1 | 0 | 0 | 1 | 0 | 0 | 0 | 0 | 0 |
| Dialytrichia saxicola* | 0 | 1 | 0 | 0 | 0 | 0 | 0 | 0 | 0 | 0 |
| Dichodontium flavescens | 0 | 1 | 0 | 0 | 0 | 0 | 0 | 0 | 0 | 0 |
| Dichodontium pellucidum | 0 | 1 | 0 | 0 | 0 | 0 | 0 | 0 | 0 | 0 |
| Dicranella ascensionica* | 0 | 0 | 0 | 0 | 0 | 0 | 0 | 1 | 0 | 0 |
| Dicranella campylophylla | 0 | 1 | 0 | 0 | 0 | 0 | 0 | 0 | 0 | 0 |
| Dicranella falcularia | 0 | 0 | 1 | 0 | 0 | 0 | 0 | 0 | 0 | 0 |
| Dicranella fuegiana | 0 | 0 | 0 | 0 | 0 | 0 | 0 | 0 | 0 | 1 |
| Dicranella goughii* | 0 | 0 | 0 | 0 | 0 | 0 | 0 | 1 | 0 | 0 |
| Dicranella heteromalla | 1 | 1 | 0 | 0 | 1 | 0 | 0 | 0 | 0 | 0 |
| Dicranella hookeri | 0 | 0 | 0 | 0 | 0 | 0 | 0 | 0 | 0 | 1 |
| Dicranella howei | 1 | 1 | 0 | 0 | 1 | 0 | 0 | 0 | 0 | 0 |
| Dicranella humilis | 0 | 1 | 0 | 0 | 0 | 0 | 0 | 0 | 0 | 0 |
| Dicranella lindigiana | 0 | 0 | 0 | 0 | 0 | 1 | 0 | 0 | 0 | 0 |
| Dicranella minuta | 0 | 0 | 0 | 0 | 0 | 0 | 0 | 1 | 0 | 0 |
| Dicranella proscripta* | 0 | 0 | 0 | 0 | 0 | 0 | 0 | 0 | 1 | 0 |
| Dicranella rufescens | 0 | 1 | 0 | 0 | 0 | 0 | 0 | 0 | 0 | 0 |
| Dicranella schreberiana | 1 | 0 | 0 | 0 | 0 | 0 | 0 | 0 | 0 | 0 |
| Dicranella staphylina* | 0 | 0 | 0 | 0 | 1 | 0 | 0 | 0 | 0 | 0 |
| Dicranella subulata | 1 | 0 | 0 | 0 | 0 | 0 | 0 | 0 | 0 | 0 |
| Dicranella vaginata | 0 | 0 | 0 | 0 | 0 | 0 | 0 | 0 | 0 | 1 |
| Dicranella varia | 0 | 1 | 0 | 0 | 1 | 0 | 1 | 0 | 0 | 0 |
| Dicranella sp. | 0 | 0 | 0 | 0 | 0 | 0 | 0 | 1 | 1 | 0 |
| Dicranodontium insulare* | 1 | 0 | 0 | 0 | 0 | 0 | 0 | 0 | 0 | 0 |
| Dicranodontium tristaniense* | 1 | 0 | 0 | 0 | 0 | 0 | 0 | 0 | 0 | 0 |
| Dicranoloma atlanticum* | 0 | 0 | 0 | 0 | 0 | 0 | 0 | 0 | 0 | 1 |
| Dicranoloma hariotii | 0 | 0 | 0 | 0 | 0 | 0 | 0 | 0 | 0 | 1 |
| Dicranoloma imponens | 0 | 0 | 0 | 0 | 0 | 0 | 0 | 0 | 0 | 1 |
| Dicranoloma perdecurrens* | 0 | 0 | 0 | 0 | 0 | 0 | 0 | 0 | 0 | 1 |
| Dicranoweisia antarctica | 0 | 0 | 0 | 0 | 0 | 0 | 0 | 0 | 0 | 1 |
| Dicranoweisia cirrata | 1 | 1 | 0 | 0 | 0 | 0 | 0 | 0 | 0 | 0 |
| Dicranoweisia falcifolia* | 0 | 0 | 0 | 0 | 0 | 0 | 0 | 0 | 0 | 1 |
| Dicranum bonjeanii | 1 | 0 | 0 | 0 | 0 | 0 | 0 | 0 | 0 | 0 |
| Dicranum flagellare | 1 | 1 | 0 | 0 | 1 | 0 | 0 | 0 | 0 | 0 |
| Dicranum fuscescens | 0 | 1 | 0 | 0 | 0 | 0 | 0 | 0 | 0 | 0 |
| Dicranum montanum | 1 | 1 | 0 | 0 | 0 | 0 | 0 | 0 | 0 | 0 |
| Dicranum scoparium | 1 | 1 | 0 | 0 | 1 | 0 | 0 | 0 | 0 | 0 |
| Dicranum scottianum* | 1 | 1 | 0 | 0 | 1 | 0 | 0 | 0 | 0 | 0 |
| Didymodon acutus | 1 | 1 | 0 | 1 | 1 | 0 | 0 | 0 | 0 | 0 |
| Didymodon australasiae | 0 | 1 | 0 | 1 | 1 | 0 | 0 | 0 | 0 | 0 |
| Didymodon austroalpigena | 0 | 0 | 0 | 0 | 0 | 0 | 0 | 0 | 0 | 1 |
| Didymodon brachyphyllus | 0 | 0 | 0 | 0 | 1 | 0 | 0 | 1 | 0 | 0 |
| Didymodon caboverdeanus* | 0 | 0 | 0 | 1 | 0 | 0 | 0 | 0 | 0 | 0 |
| Didymodon fallax | 0 | 1 | 0 | 1 | 1 | 0 | 0 | 0 | 0 | 0 |
| Didymodon hastatus | 0 | 0 | 0 | 1 | 0 | 0 | 0 | 0 | 0 | 0 |
| Didymodon insulanus | 1 | 1 | 0 | 1 | 1 | 0 | 0 | 0 | 0 | 0 |
| Didymodon nicholsonii | 0 | 0 | 0 | 0 | 1 | 0 | 0 | 0 | 0 | 0 |
| Didymodon revolutus | 0 | 0 | 0 | 1 | 0 | 0 | 0 | 0 | 0 | 0 |
| Didymodon rigidulus | 1 | 1 | 0 | 1 | 1 | 0 | 0 | 0 | 0 | 0 |
| Didymodon tophaceus | 1 | 1 | 0 | 1 | 1 | 0 | 0 | 0 | 0 | 0 |
| Didymodon umbrosus | 0 | 0 | 0 | 0 | 1 | 0 | 0 | 0 | 0 | 0 |
| Didymodon vinealis | 1 | 1 | 0 | 1 | 1 | 0 | 0 | 0 | 0 | 0 |
| Didymodon sp. | 0 | 0 | 0 | 0 | 0 | 0 | 0 | 0 | 1 | 0 |
| Diphyscium foliosum | 1 | 1 | 0 | 0 | 1 | 0 | 0 | 0 | 0 | 0 |
| Distichium capillaceum | 0 | 0 | 0 | 0 | 1 | 0 | 0 | 0 | 0 | 0 |
| Distichophyllum fasciculatum | 0 | 0 | 0 | 0 | 0 | 0 | 0 | 0 | 0 | 1 |
| Ditrichum conicum | 0 | 0 | 0 | 0 | 0 | 0 | 0 | 0 | 0 | 1 |
| Ditrichum difficile | 0 | 0 | 0 | 0 | 0 | 0 | 0 | 0 | 0 | 1 |
| Ditrichum hyalinum | 0 | 0 | 0 | 0 | 0 | 0 | 0 | 0 | 0 | 1 |
| Ditrichum pallidum | 1 | 1 | 0 | 0 | 0 | 0 | 0 | 0 | 0 | 0 |
| Ditrichum punctulatum | 1 | 1 | 0 | 0 | 0 | 0 | 0 | 0 | 0 | 0 |
| Ditrichum pusillum | 0 | 0 | 0 | 0 | 1 | 0 | 0 | 0 | 0 | 0 |
| Ditrichum subulatum | 1 | 0 | 0 | 0 | 1 | 0 | 0 | 0 | 0 | 1 |
| Ditricum strictum | 0 | 0 | 0 | 0 | 0 | 0 | 0 | 0 | 0 | 1 |
| Ditrichum tenuinerve* | 0 | 0 | 0 | 0 | 0 | 0 | 0 | 0 | 0 | 1 |
| Ditrichum sp. | 0 | 0 | 0 | 1 | 0 | 0 | 0 | 0 | 0 | 0 |
| Drepanocladus aduncus | 0 | 0 | 0 | 0 | 1 | 0 | 0 | 0 | 0 | 0 |
| Drepanocladus polygamus | 0 | 0 | 0 | 0 | 0 | 0 | 0 | 0 | 0 | 1 |
| Echinodium renauldii | 1 | 0 | 0 | 0 | 0 | 0 | 0 | 0 | 0 | 0 |
| Echinodium setigerum* | 0 | 1 | 0 | 0 | 0 | 0 | 0 | 0 | 0 | 0 |
| Echinodium spinosum* | 0 | 1 | 0 | 0 | 1 | 0 | 0 | 0 | 0 | 0 |
| Ectropothecium brevifalcatum | 0 | 0 | 1 | 0 | 0 | 0 | 0 | 0 | 0 | 0 |
| Ectropothecium diffusum | 0 | 0 | 1 | 0 | 0 | 0 | 0 | 0 | 0 | 0 |
| Ectropothecium drepanophyllum | 0 | 0 | 1 | 0 | 0 | 0 | 0 | 0 | 0 | 0 |
| Encalypta streptocarpa | 0 | 0 | 0 | 0 | 1 | 0 | 0 | 0 | 0 | 0 |
| Encalypta vulgaris | 0 | 1 | 0 | 0 | 1 | 0 | 0 | 0 | 0 | 0 |
| Entodon dregeanus | 0 | 0 | 0 | 0 | 0 | 0 | 0 | 0 | 1 | 0 |
| Entodon pseudoseductrix | 0 | 0 | 0 | 0 | 1 | 0 | 0 | 0 | 0 | 0 |
| Entodon schleicheri | 0 | 0 | 0 | 0 | 1 | 0 | 0 | 0 | 0 | 0 |
| Entodontopsis leucostega | 0 | 0 | 0 | 1 | 0 | 0 | 0 | 0 | 0 | 0 |
| Entosthodon attenuatus | 1 | 1 | 0 | 0 | 1 | 0 | 0 | 0 | 0 | 0 |
| Entosthodon borbonicus | 0 | 0 | 0 | 0 | 0 | 0 | 0 | 1 | 0 | 0 |
| Enthostodon commutatus | 0 | 1 | 0 | 0 | 1 | 0 | 0 | 0 | 0 | 0 |
| Entosthodon convexus | 0 | 1 | 0 | 0 | 1 | 0 | 0 | 0 | 0 | 0 |
| Entosthodon durieuii | 0 | 0 | 0 | 0 | 1 | 0 | 0 | 0 | 0 | 0 |
| Entosthodon fascicularis | 0 | 1 | 0 | 0 | 1 | 0 | 0 | 0 | 0 | 0 |
| Entosthodon hungaricus | 0 | 0 | 0 | 0 | 1 | 0 | 0 | 0 | 0 | 0 |
| Enthostodon kroonkurk* | 0 | 0 | 0 | 1 | 1 | 0 | 0 | 0 | 0 | 0 |
| Entosthodon muhlenbergii | 0 | 1 | 0 | 0 | 0 | 0 | 0 | 0 | 0 | 0 |
| Entosthodon obtusus | 1 | 1 | 0 | 0 | 1 | 0 | 0 | 0 | 0 | 0 |
| Entosthodon pulchellus | 1 | 1 | 0 | 0 | 1 | 0 | 0 | 0 | 0 | 0 |
| Entosthodon schimperi | 0 | 0 | 0 | 0 | 1 | 0 | 0 | 0 | 0 | 0 |
| Entosthodon sp. | 0 | 0 | 0 | 0 | 0 | 0 | 0 | 0 | 1 | 0 |
| Ephemerum cohaerens | 1 | 0 | 0 | 0 | 0 | 0 | 0 | 0 | 0 | 0 |
| Ephemerum crassinervium | 0 | 1 | 0 | 0 | 0 | 0 | 0 | 0 | 0 | 0 |
| Ephemerum serratum | 0 | 1 | 0 | 0 | 0 | 0 | 0 | 0 | 0 | 0 |
| Ephemerum stoloniferum | 0 | 1 | 0 | 0 | 0 | 0 | 0 | 0 | 0 | 0 |
| Epipterygium atlanticum* | 1 | 0 | 0 | 0 | 0 | 0 | 0 | 0 | 0 | 0 |
| Epipterygium tozeri | 1 | 1 | 0 | 1 | 1 | 0 | 0 | 0 | 0 | 0 |
| Eucladium verticillatum | 1 | 1 | 0 | 1 | 1 | 0 | 0 | 0 | 0 | 0 |
| Eurhynchium speciosum | 0 | 0 | 0 | 1 | 0 | 0 | 0 | 0 | 0 | 0 |
| Eurhynchium striatum | 1 | 1 | 0 | 0 | 1 | 0 | 0 | 0 | 0 | 0 |
| Eustichia longirostris | 0 | 0 | 0 | 0 | 0 | 0 | 0 | 0 | 0 | 1 |
| Exsertotheca crispa | 1 | 1 | 0 | 0 | 0 | 0 | 0 | 0 | 0 | 0 |
| Exsertotheca intermedia* | 1 | 1 | 0 | 0 | 0 | 0 | 0 | 0 | 0 | 0 |
| Fabronia leikipiae | 0 | 0 | 0 | 1 | 0 | 0 | 0 | 0 | 0 | 0 |
| Fabronia pusilla | 0 | 1 | 0 | 0 | 1 | 0 | 0 | 0 | 0 | 0 |
| Fissidens adianthoides | 1 | 1 | 0 | 0 | 1 | 0 | 0 | 0 | 0 | 0 |
| Fissidens allorgei* | 0 | 0 | 0 | 1 | 0 | 0 | 0 | 0 | 0 | 0 |
| Fissidens androgynus | 0 | 0 | 0 | 1 | 0 | 0 | 0 | 0 | 0 | 0 |
| Fissidens asplenioides | 1 | 1 | 1 | 0 | 1 | 0 | 0 | 0 | 0 | 1 |
| Fissidens azoricus* | 1 | 0 | 0 | 0 | 0 | 0 | 0 | 0 | 0 | 0 |
| Fissidens bogosicus | 0 | 0 | 0 | 1 | 0 | 0 | 0 | 0 | 0 | 0 |
| Fissidens borgenii | 0 | 0 | 0 | 0 | 1 | 0 | 0 | 0 | 0 | 0 |
| Fissidens bryoides var. bryoides | 1 | 1 | 0 | 0 | 1 | 0 | 1 | 0 | 0 | 0 |
| Fissidens bryoides var. caespitans | 0 | 1 | 0 | 0 | 0 | 0 | 0 | 0 | 0 | 0 |
| Fissidens chioneurus* | 0 | 0 | 0 | 0 | 0 | 0 | 0 | 0 | 1 | 0 |
| Fissidens coacervatus* | 1 | 1 | 0 | 0 | 1 | 0 | 0 | 0 | 0 | 0 |
| Fissidens crassipes subsp. crassipes | 1 | 1 | 0 | 0 | 1 | 0 | 0 | 0 | 0 | 0 |
| Fissidens crassipes subsp. warnstorfii | 1 | 1 | 0 | 0 | 0 | 0 | 0 | 0 | 0 | 0 |
| Fissidens crispulus var. crispulus | 0 | 0 | 1 | 0 | 0 | 0 | 0 | 0 | 0 | 0 |
| Fissidens crispulus var. robinsonii | 0 | 0 | 1 | 0 | 0 | 0 | 0 | 0 | 0 | 0 |
| Fissidens crispus | 1 | 1 | 1 | 1 | 1 | 0 | 1 | 0 | 0 | 0 |
| Fissidens cryptoneuron | 0 | 0 | 0 | 0 | 0 | 0 | 1 | 0 | 0 | 0 |
| Fissidens curvatus subsp. curvatus | 1 | 1 | 0 | 0 | 1 | 0 | 1 | 0 | 0 | 0 |
| Fissidens curvatus subsp. sanctae-helenae* | 0 | 0 | 0 | 0 | 0 | 0 | 0 | 0 | 1 | 0 |
| Fissidens danckelmannii | 0 | 0 | 0 | 1 | 0 | 0 | 0 | 0 | 0 | 0 |
| Fissidens darntyi | 0 | 0 | 1 | 0 | 0 | 0 | 0 | 1 | 1 | 0 |
| Fissidens dubius | 1 | 1 | 0 | 0 | 1 | 0 | 0 | 0 | 0 | 0 |
| Fissidens elegans | 0 | 0 | 0 | 0 | 0 | 0 | 0 | 0 | 1 | 0 |
| Fissidens enervis | 0 | 0 | 1 | 0 | 0 | 0 | 0 | 0 | 0 | 0 |
| Fissidens exilis | 0 | 0 | 0 | 1 | 0 | 0 | 0 | 0 | 0 | 0 |
| Fissidens flaccidus | 0 | 0 | 1 | 1 | 0 | 0 | 0 | 0 | 0 | 0 |
| Fissidens fluitans* | 0 | 0 | 0 | 0 | 0 | 0 | 0 | 0 | 0 | 1 |
| Fissidens fontanus | 0 | 1 | 0 | 0 | 0 | 0 | 0 | 0 | 0 | 0 |
| Fissidens glaucissimus | 0 | 0 | 1 | 0 | 0 | 0 | 0 | 0 | 0 | 0 |
| Fissidens gracilifolius | 0 | 0 | 1 | 0 | 0 | 0 | 0 | 0 | 0 | 0 |
| Fissidens hornschuchii | 0 | 0 | 0 | 0 | 0 | 1 | 0 | 0 | 0 | 0 |
| Fissidens incurvus | 1 | 1 | 0 | 0 | 1 | 0 | 0 | 0 | 0 | 0 |
| Fissidens intromarginatus | 0 | 0 | 1 | 0 | 0 | 0 | 0 | 0 | 0 | 0 |
| Fissidens megalotis subsp. helictocaulos | 0 | 0 | 0 | 1 | 0 | 0 | 0 | 0 | 0 | 0 |
| Fissidens metzgeria | 0 | 0 | 1 | 0 | 0 | 0 | 0 | 0 | 0 | 0 |
| Fissidens microcarpus | 0 | 0 | 1 | 0 | 0 | 0 | 0 | 0 | 0 | 0 |
| Fissidens microstictus* | 0 | 1 | 0 | 0 | 0 | 0 | 0 | 0 | 0 | 0 |
| Fissidens minutulus | 0 | 0 | 0 | 1 | 0 | 0 | 0 | 0 | 0 | 0 |
| Fissidens monguillonii | 1 | 1 | 0 | 0 | 0 | 0 | 0 | 0 | 0 | 0 |
| Fissidens nobreganus* | 0 | 1 | 0 | 0 | 0 | 0 | 0 | 0 | 0 | 0 |
| Fissidens noronhensis* | 0 | 0 | 0 | 0 | 0 | 0 | 1 | 0 | 0 | 0 |
| Fissidens ovatifolius | 1 | 1 | 0 | 0 | 1 | 0 | 0 | 0 | 0 | 0 |
| Fissidens ovatus | 0 | 0 | 1 | 0 | 0 | 0 | 0 | 0 | 0 | 0 |
| Fissidens pallidinervis | 0 | 0 | 1 | 0 | 0 | 0 | 0 | 0 | 0 | 0 |
| Fissidens palmatus | 0 | 0 | 1 | 0 | 0 | 0 | 1 | 0 | 0 | 0 |
| Fissidens pellucidus | 0 | 0 | 1 | 0 | 0 | 0 | 0 | 0 | 0 | 0 |
| Fissidens polyphyllus | 0 | 1 | 0 | 0 | 1 | 0 | 0 | 0 | 0 | 0 |
| Fissidens porrectus | 0 | 0 | 1 | 0 | 0 | 0 | 0 | 0 | 1 | 0 |
| Fissidens punctulatus | 0 | 0 | 1 | 0 | 0 | 0 | 0 | 0 | 0 | 0 |
| Fissidens pusillus | 1 | 1 | 0 | 0 | 0 | 0 | 0 | 0 | 0 | 0 |
| Fissidens pygmaeus | 0 | 0 | 0 | 0 | 0 | 0 | 0 | 0 | 1 | 0 |
| Fissidens ramulosus | 0 | 0 | 1 | 0 | 0 | 0 | 0 | 0 | 0 | 0 |
| Fissidens reimersii***** | 0 | 0 | 0 | 0 | 0 | 0 | 0 | 0 | 1 | 0 |
| Fissidens rivularis | 1 | 1 | 0 | 0 | 1 | 0 | 0 | 0 | 0 | 0 |
| Fissidens sciophyllus | 0 | 0 | 1 | 1 | 0 | 0 | 0 | 0 | 0 | 0 |
| Fissidens serratus | 1 | 1 | 1 | 0 | 1 | 0 | 0 | 1 | 1 | 0 |
| Fissidens serratus var. leptochaete | 0 | 0 | 0 | 0 | 0 | 0 | 0 | 0 | 0 | 1 |
| Fissidens serrulatus | 1 | 1 | 0 | 0 | 1 | 0 | 0 | 0 | 0 | 0 |
| Fissidens sublimbatus | 0 | 1 | 0 | 0 | 1 | 0 | 0 | 0 | 0 | 0 |
| Fissidens sublinaefolius | 1 | 1 | 0 | 0 | 0 | 0 | 0 | 0 | 0 | 0 |
| Fissidens submarginatus | 0 | 0 | 1 | 0 | 0 | 0 | 1 | 0 | 0 | 0 |
| Fissidens subobtusus* | 0 | 0 | 0 | 0 | 0 | 0 | 0 | 0 | 0 | 1 |
| Fissidens taxifolius | 1 | 1 | 0 | 0 | 1 | 0 | 0 | 0 | 1 | 0 |
| Fissidens taylorii | 0 | 0 | 0 | 0 | 0 | 0 | 0 | 0 | 1 | 0 |
| Fissidens transluscens* | 0 | 0 | 0 | 0 | 0 | 0 | 0 | 0 | 1 | 1 |
| Fissidens usambaricus | 0 | 0 | 1 | 1 | 0 | 0 | 0 | 0 | 0 | 0 |
| Fissidens viridulus var. viridulus | 0 | 1 | 0 | 0 | 1 | 0 | 0 | 0 | 0 | 0 |
| Fissidens zollingeri | 0 | 0 | 1 | 0 | 0 | 1 | 1 | 1 | 0 | 0 |
| Fissidens sp. | 0 | 1 | 0 | 0 | 0 | 0 | 0 | 0 | 0 | 0 |
| Flexitrichum flexicaule | 0 | 1 | 0 | 0 | 0 | 0 | 0 | 0 | 0 | 0 |
| Floribundaria floribunda | 0 | 0 | 1 | 0 | 0 | 0 | 0 | 0 | 0 | 0 |
| Floribundaria vaginans | 0 | 0 | 1 | 0 | 0 | 0 | 0 | 0 | 0 | 0 |
| Fontinalis antipyretica | 1 | 1 | 0 | 0 | 1 | 0 | 0 | 0 | 0 | 0 |
| Funaria acicularis* | 0 | 0 | 1 | 0 | 0 | 0 | 0 | 0 | 0 | 0 |
| Funaria chevalieri* | 0 | 0 | 0 | 1 | 0 | 0 | 0 | 0 | 0 | 0 |
| Funaria euryloma* | 0 | 0 | 0 | 0 | 0 | 0 | 0 | 0 | 0 | 1 |
| Funaria hygrometrica | 1 | 1 | 1 | 1 | 1 | 0 | 0 | 1 | 0 | 1 |
| Funariella curviseta | 0 | 0 | 0 | 0 | 1 | 0 | 0 | 0 | 0 | 0 |
| Geheebia lurida | 1 | 1 | 0 | 0 | 1 | 0 | 0 | 0 | 0 | 0 |
| Geheebia maschalogena | 0 | 0 | 0 | 1 | 0 | 0 | 0 | 0 | 0 | 0 |
| Geheebia siccula | 1 | 1 | 0 | 1 | 1 | 0 | 0 | 0 | 0 | 0 |
| Gigaspermum mouretii | 1 | 1 | 0 | 0 | 0 | 0 | 0 | 0 | 0 | 0 |
| Glyphomitrium daviesii | 1 | 1 | 0 | 0 | 0 | 0 | 0 | 0 | 0 | 0 |
| Goniomitrium seroi* | 0 | 0 | 0 | 0 | 1 | 0 | 0 | 0 | 0 | 0 |
| Grimmia anodon | 0 | 0 | 0 | 0 | 1 | 0 | 0 | 0 | 0 | 0 |
| Grimmia arenaria | 0 | 1 | 0 | 0 | 1 | 0 | 0 | 0 | 0 | 0 |
| Grimmia crinita | 0 | 0 | 0 | 0 | 1 | 0 | 0 | 0 | 0 | 0 |
| Grimmia decipiens | 0 | 1 | 0 | 0 | 1 | 0 | 0 | 0 | 0 | 0 |
| Grimmia dissimulata | 0 | 1 | 0 | 0 | 0 | 0 | 0 | 0 | 0 | 0 |
| Grimmia donniana | 0 | 1 | 0 | 0 | 0 | 0 | 0 | 0 | 0 | 0 |
| Grimmia elongata | 1 | 0 | 0 | 0 | 0 | 0 | 0 | 0 | 0 | 0 |
| Grimmia funalis | 0 | 1 | 0 | 0 | 1 | 0 | 0 | 0 | 0 | 0 |
| Grimmia hartmanii | 1 | 0 | 0 | 0 | 0 | 0 | 0 | 0 | 0 | 0 |
| Grimmia incurva | 1 | 0 | 0 | 1 | 0 | 0 | 0 | 0 | 0 | 0 |
| Grimmia kidderi | 0 | 0 | 0 | 0 | 0 | 0 | 0 | 0 | 0 | 1 |
| Grimmia laevigata | 1 | 1 | 0 | 1 | 1 | 0 | 0 | 0 | 0 | 0 |
| Grimmia lisae | 1 | 1 | 0 | 1 | 1 | 0 | 0 | 0 | 0 | 0 |
| Grimmia longirostris | 0 | 0 | 0 | 0 | 1 | 0 | 0 | 0 | 0 | 0 |
| Grimmia meridionalis | 1 | 1 | 0 | 0 | 1 | 0 | 0 | 0 | 0 | 0 |
| Grimmia montana | 1 | 1 | 0 | 0 | 1 | 0 | 0 | 0 | 0 | 0 |
| Grimmia nutans | 0 | 0 | 0 | 0 | 1 | 0 | 0 | 0 | 0 | 0 |
| Grimmia orbicularis | 0 | 1 | 0 | 0 | 1 | 0 | 0 | 0 | 0 | 0 |
| Grimmia ovalis | 0 | 1 | 0 | 0 | 1 | 0 | 0 | 0 | 0 | 0 |
| Grimmia pulvinata | 1 | 1 | 0 | 0 | 1 | 0 | 0 | 0 | 0 | 0 |
| Grimmia ramondii | 0 | 1 | 0 | 0 | 1 | 0 | 0 | 0 | 0 | 0 |
| Grimmia tergestina | 0 | 0 | 0 | 0 | 1 | 0 | 0 | 0 | 0 | 0 |
| Grimmia torquata | 0 | 1 | 0 | 0 | 1 | 0 | 0 | 0 | 0 | 0 |
| Grimmia trichophylla | 0 | 1 | 0 | 1 | 1 | 0 | 0 | 0 | 0 | 0 |
| Groutiella tomentosa | 0 | 0 | 0 | 1 | 0 | 0 | 0 | 0 | 0 | 0 |
| Gymnostomiella erosula | 0 | 0 | 1 | 1 | 0 | 0 | 0 | 0 | 0 | 0 |
| Gymnostomiella vernicosa | 0 | 0 | 1 | 0 | 0 | 0 | 0 | 0 | 0 | 0 |
| Gymnostomiella vernicosa var. monodii | 0 | 0 | 0 | 1 | 0 | 0 | 0 | 0 | 0 | 0 |
| Gymnostomiella vernicosa var. tenera | 0 | 0 | 0 | 1 | 0 | 0 | 0 | 0 | 0 | 0 |
| Gymnostomum aeruginosum | 0 | 1 | 0 | 1 | 1 | 0 | 0 | 0 | 0 | 0 |
| Gymnostomum bescherellei* | 0 | 0 | 0 | 0 | 0 | 0 | 0 | 1 | 0 | 0 |
| Gymnostomum calcareum | 1 | 1 | 0 | 1 | 1 | 0 | 0 | 0 | 0 | 1 |
| Gymnostomum calcareum var. atlanticum | 1 | 1 | 0 | 0 | 0 | 0 | 0 | 0 | 0 | 0 |
| Gymnostomum viridulum | 1 | 1 | 0 | 0 | 0 | 0 | 0 | 0 | 0 | 0 |
| Gyroweisia reflexa | 0 | 1 | 0 | 0 | 1 | 0 | 0 | 0 | 0 | 0 |
| Gyroweisia tenuis | 1 | 1 | 0 | 0 | 0 | 0 | 0 | 0 | 0 | 0 |
| Habrodon perpusillus | 0 | 1 | 0 | 0 | 1 | 0 | 0 | 0 | 0 | 0 |
| Haplocladium angustifolium | 0 | 0 | 0 | 1 | 1 | 0 | 0 | 0 | 0 | 0 |
| Hedenasiastrum percurrens* | 0 | 1 | 0 | 0 | 0 | 0 | 0 | 0 | 0 | 0 |
| Hedwigia ciliata | 0 | 1 | 0 | 1 | 1 | 0 | 0 | 0 | 0 | 0 |
| Hedwigia stellata | 0 | 1 | 0 | 0 | 0 | 0 | 0 | 0 | 0 | 0 |
| Hennediella kunzeana | 0 | 0 | 0 | 0 | 0 | 0 | 0 | 0 | 0 | 1 |
| Herpetineuron toccoae | 0 | 0 | 0 | 1 | 0 | 0 | 0 | 0 | 0 | 0 |
| Herzogiella striatella | 1 | 0 | 0 | 0 | 0 | 0 | 0 | 0 | 0 | 0 |
| Heterocladium flaccidum | 1 | 1 | 0 | 0 | 0 | 0 | 0 | 0 | 0 | 0 |
| Heterocladium heteropterum | 1 | 1 | 0 | 0 | 0 | 0 | 0 | 0 | 0 | 0 |
| Heterocladium wulfsbergii | 1 | 1 | 0 | 0 | 1 | 0 | 0 | 0 | 0 | 0 |
| Homalia lusitanica | 1 | 1 | 0 | 0 | 1 | 0 | 0 | 0 | 0 | 0 |
| Homalocladium implanum | 0 | 0 | 0 | 0 | 0 | 0 | 0 | 1 | 0 | 0 |
| Homalothecium aureum | 0 | 1 | 0 | 1 | 1 | 0 | 0 | 0 | 0 | 0 |
| Homalothecium lutescens | 0 | 1 | 0 | 0 | 1 | 0 | 0 | 0 | 0 | 0 |
| Homalothecium meridionale | 0 | 0 | 0 | 0 | 1 | 0 | 0 | 0 | 0 | 0 |
| Homalothecium mandonii* | 1 | 1 | 0 | 1 | 1 | 0 | 0 | 0 | 0 | 0 |
| Hookeria lucens | 1 | 1 | 0 | 0 | 0 | 0 | 0 | 0 | 0 | 0 |
| Hookeriopsis crispifolia | 0 | 0 | 0 | 0 | 0 | 0 | 0 | 0 | 1 | 0 |
| Hookeriopsis pallidifolia | 0 | 0 | 0 | 0 | 0 | 0 | 0 | 1 | 1 | 0 |
| Hydrogonium bolleanum | 1 | 1 | 0 | 1 | 0 | 0 | 0 | 0 | 0 | 0 |
| Hydrogonium consanguineum | 0 | 0 | 0 | 1 | 0 | 0 | 0 | 0 | 0 | 0 |
| Hydrogonium orientale | 0 | 0 | 1 | 1 | 0 | 0 | 0 | 0 | 0 | 0 |
| Hygroamblystegium fluviatile | 1 | 1 | 0 | 0 | 0 | 0 | 0 | 0 | 0 | 0 |
| Hygroamblystegium fuegianum | 0 | 0 | 0 | 0 | 0 | 0 | 0 | 0 | 0 | 1 |
| Hygroamblystegium fuegianum var. excurrens | 0 | 0 | 0 | 0 | 0 | 0 | 0 | 0 | 0 | 1 |
| Hygroamblystegium humile | 1 | 1 | 0 | 0 | 0 | 0 | 0 | 0 | 0 | 0 |
| Hygroamblystegium tenax | 1 | 1 | 0 | 0 | 0 | 0 | 0 | 0 | 0 | 0 |
| Hygroamblystegium varium | 1 | 1 | 0 | 0 | 1 | 0 | 0 | 0 | 0 | 0 |
| Hygrohypnum luridum | 1 | 0 | 0 | 0 | 0 | 0 | 0 | 0 | 0 | 0 |
| Hylocomiadelphus triquetus | 0 | 1 | 0 | 0 | 0 | 0 | 0 | 0 | 0 | 0 |
| Hylocomium splendens | 0 | 1 | 0 | 0 | 1 | 0 | 0 | 0 | 0 | 0 |
| Hymenoloma crispulum | 1 | 0 | 0 | 0 | 0 | 0 | 0 | 0 | 0 | 0 |
| Hymenophyton flabellatum | 0 | 0 | 0 | 0 | 0 | 0 | 0 | 0 | 0 | 1 |
| Hymenostylium recurvirostrum | 0 | 1 | 1 | 0 | 0 | 0 | 0 | 1 | 0 | 0 |
| Hyocomium armoricum | 1 | 1 | 0 | 0 | 0 | 0 | 0 | 0 | 0 | 0 |
| Hyophila ascensionis* | 0 | 0 | 0 | 0 | 0 | 0 | 1 | 0 | 0 | 0 |
| Hyophila involuta | 0 | 0 | 1 | 1 | 0 | 0 | 0 | 0 | 0 | 0 |
| Hyophiladelphus agrarius | 0 | 0 | 0 | 0 | 0 | 1 | 1 | 0 | 0 | 0 |
| Hypnum andoi | 1 | 1 | 0 | 0 | 1 | 0 | 0 | 0 | 0 | 0 |
| Hypnum chrypogaster | 0 | 0 | 0 | 0 | 0 | 0 | 0 | 0 | 1 | 0 |
| Hypnum cupressiforme | 1 | 1 | 0 | 1 | 1 | 0 | 0 | 0 | 1 | 1 |
| Hypnum cupressiforme var. lacunosum | 0 | 1 | 0 | 0 | 0 | 0 | 0 | 0 | 0 | 0 |
| Hypnum cupressiforme var. subjulaceum | 0 | 0 | 0 | 0 | 1 | 0 | 0 | 0 | 0 | 0 |
| Hypnum jutlandicum | 1 | 1 | 0 | 0 | 1 | 0 | 0 | 0 | 0 | 0 |
| Hypnum lacunosum | 0 | 0 | 0 | 0 | 0 | 0 | 0 | 1 | 0 | 0 |
| Hypnum resupinatum | 1 | 1 | 0 | 0 | 1 | 0 | 0 | 0 | 0 | 0 |
| Hypnum uncinulatum | 1 | 1 | 0 | 0 | 1 | 0 | 0 | 0 | 0 | 0 |
| Hypopterygium tamarisci | 0 | 0 | 1 | 0 | 0 | 0 | 0 | 0 | 0 | 0 |
| Imbibryum alpinum | 1 | 1 | 0 | 0 | 1 | 0 | 0 | 0 | 0 | 0 |
| Imbibryum mildeanum | 1 | 1 | 0 | 0 | 0 | 0 | 0 | 0 | 0 | 0 |
| Imbribryum muehlenbeckii | 0 | 1 | 0 | 0 | 0 | 0 | 0 | 0 | 0 | 0 |
| Imbibryum supapiculatum | 1 | 1 | 0 | 0 | 1 | 0 | 0 | 0 | 0 | 0 |
| Imbibryum tenuisetum | 1 | 0 | 0 | 0 | 0 | 0 | 0 | 0 | 0 | 0 |
| Isopterygiopsis pulchella | 1 | 1 | 0 | 0 | 1 | 0 | 0 | 0 | 0 | 0 |
| Isopterygium brownii | 0 | 0 | 0 | 0 | 0 | 0 | 0 | 0 | 0 | 1 |
| Isopterygium nanoglobum* | 0 | 0 | 1 | 0 | 0 | 0 | 0 | 0 | 0 | 0 |
| Isopterygium tenerifolium | 0 | 0 | 0 | 0 | 0 | 1 | 0 | 0 | 0 | 0 |
| Isopterygium tenerum | 0 | 1 | 0 | 0 | 0 | 0 | 0 | 0 | 0 | 0 |
| Isopterygium sp. | 0 | 0 | 0 | 0 | 0 | 0 | 0 | 0 | 1 | 0 |
| Isothecium algarvicum | 0 | 1 | 0 | 0 | 1 | 0 | 0 | 0 | 0 | 0 |
| Isothecium alopecuroides | 1 | 0 | 0 | 0 | 1 | 0 | 0 | 0 | 0 | 0 |
| Isothecium montanum* | 0 | 1 | 0 | 0 | 0 | 0 | 0 | 0 | 0 | 0 |
| Isothecium myosuroides subsp. myosuroides | 1 | 1 | 0 | 1 | 1 | 0 | 0 | 0 | 0 | 0 |
| Isothecium prolixum* | 1 | 1 | 0 | 0 | 0 | 0 | 0 | 0 | 0 | 0 |
| Itatiella tristaniense* | 0 | 0 | 0 | 0 | 0 | 0 | 0 | 0 | 0 | 1 |
| Kiaeria blyttii | 1 | 0 | 0 | 0 | 0 | 0 | 0 | 0 | 0 | 0 |
| Kindbergia praelonga | 0 | 0 | 0 | 0 | 0 | 0 | 0 | 0 | 1 | 0 |
| Lepidopilum lastii | 0 | 0 | 1 | 0 | 0 | 0 | 0 | 0 | 0 | 0 |
| Lepidopilum niveum | 0 | 0 | 1 | 0 | 0 | 0 | 0 | 0 | 0 | 0 |
| Leptobarbula berica | 1 | 1 | 0 | 0 | 0 | 0 | 0 | 0 | 0 | 0 |
| Leptobryum pyriforme | 1 | 1 | 0 | 1 | 1 | 0 | 0 | 0 | 0 | 0 |
| Leptodictyum riparium | 1 | 1 | 0 | 1 | 1 | 0 | 0 | 0 | 0 | 0 |
| Leptodon longisetus | 0 | 1 | 0 | 1 | 1 | 0 | 0 | 0 | 0 | 0 |
| Leptodon smithii | 0 | 1 | 0 | 1 | 1 | 0 | 0 | 0 | 0 | 0 |
| Leptodontium flexifolium | 0 | 1 | 0 | 0 | 0 | 0 | 0 | 0 | 0 | 0 |
| Leptodontium viticulosoides | 0 | 0 | 0 | 1 | 0 | 0 | 0 | 0 | 0 | 0 |
| Lepyrodon alaris* | 0 | 0 | 0 | 0 | 0 | 0 | 0 | 0 | 0 | 1 |
| Lescuraea mutabilis | 0 | 0 | 0 | 0 | 1 | 0 | 0 | 0 | 0 | 0 |
| Leskea polycarpa | 0 | 0 | 0 | 0 | 1 | 0 | 0 | 0 | 0 | 0 |
| Leucobryum albidum | 1 | 0 | 0 | 0 | 1 | 0 | 0 | 0 | 0 | 0 |
| Leucobryum fouta-djalloni | 0 | 0 | 1 | 0 | 0 | 0 | 0 | 0 | 0 | 0 |
| Leucobryum glaucum | 1 | 1 | 0 | 0 | 1 | 0 | 0 | 0 | 0 | 0 |
| Leucobrym homalophyllum* | 0 | 0 | 1 | 0 | 0 | 0 | 0 | 0 | 0 | 0 |
| Leucobryum juniperoideum | 1 | 1 | 0 | 0 | 1 | 0 | 0 | 0 | 0 | 0 |
| Leucobrym leucophanoides* | 0 | 0 | 1 | 0 | 0 | 0 | 0 | 0 | 0 | 0 |
| Leucobryum secundifolium | 0 | 0 | 1 | 0 | 0 | 0 | 0 | 0 | 0 | 0 |
| Leucodon canariensis* | 1 | 1 | 0 | 0 | 1 | 0 | 0 | 0 | 0 | 0 |
| Leucodon sciuroides | 1 | 1 | 0 | 1 | 1 | 0 | 0 | 0 | 0 | 0 |
| Leucodon smithii | 0 | 1 | 0 | 0 | 1 | 0 | 0 | 0 | 0 | 0 |
| Leucodon treleasei* | 1 | 1 | 0 | 0 | 1 | 0 | 0 | 0 | 0 | 0 |
| Leucoloma chrysobasilare | 0 | 0 | 1 | 0 | 0 | 0 | 0 | 0 | 0 | 0 |
| Leucoloma gracilescens | 0 | 0 | 1 | 0 | 0 | 0 | 0 | 0 | 0 | 0 |
| Leucoloma secundifolium | 0 | 0 | 1 | 0 | 0 | 0 | 0 | 0 | 0 | 0 |
| Leucomium strumosum | 0 | 0 | 1 | 0 | 0 | 0 | 0 | 0 | 0 | 0 |
| Leucophanes molleri | 0 | 0 | 1 | 0 | 0 | 0 | 0 | 0 | 0 | 0 |
| Leucophanes unguiculatum | 0 | 0 | 1 | 0 | 0 | 0 | 0 | 0 | 0 | 0 |
| Lewinskya acuminata | 0 | 1 | 0 | 1 | 0 | 0 | 0 | 0 | 0 | 0 |
| Lewinskya affinis | 0 | 1 | 0 | 0 | 0 | 0 | 0 | 0 | 0 | 0 |
| Lewinskya rupestris | 0 | 1 | 0 | 0 | 1 | 0 | 0 | 0 | 0 | 0 |
| Lewinskya striata | 0 | 1 | 0 | 0 | 1 | 0 | 0 | 0 | 0 | 0 |
| Lindbergia patentifolia | 0 | 0 | 0 | 1 | 0 | 0 | 0 | 0 | 0 | 0 |
| Loeskeobryum brevirostre | 1 | 0 | 0 | 0 | 0 | 0 | 0 | 0 | 0 | 0 |
| Lopidium struthiopteris | 0 | 0 | 1 | 0 | 0 | 0 | 0 | 0 | 0 | 0 |
| Macrocoma tenuis subsp. tenuis | 0 | 0 | 0 | 0 | 0 | 0 | 0 | 0 | 1 | 0 |
| Macromitrium acutirameum* | 0 | 0 | 0 | 0 | 0 | 0 | 0 | 0 | 0 | 1 |
| Macromitrium antarcticum* | 0 | 0 | 0 | 0 | 0 | 0 | 0 | 0 | 0 | 1 |
| Macromitrium fimbriatum | 0 | 0 | 0 | 0 | 0 | 0 | 0 | 0 | 0 | 1 |
| Macromitrium microstomum | 0 | 0 | 0 | 0 | 0 | 0 | 0 | 0 | 1 | 0 |
| Macromitrium sulcatum | 0 | 0 | 1 | 0 | 0 | 0 | 0 | 0 | 0 | 0 |
| Macromitrium urceolatum* | 0 | 0 | 0 | 0 | 0 | 0 | 0 | 0 | 1 | 0 |
| Meiothecium urceolatum | 0 | 0 | 0 | 0 | 0 | 0 | 0 | 0 | 0 | 1 |
| Mesonodon flavescens | 0 | 0 | 1 | 0 | 0 | 0 | 0 | 0 | 0 | 0 |
| Mesoptychia bantriensis | 0 | 1 | 0 | 0 | 0 | 0 | 0 | 0 | 0 | 0 |
| Mesoptychia heterocolpos | 0 | 1 | 0 | 0 | 0 | 0 | 0 | 0 | 0 | 0 |
| Mesoptychia turbinata | 0 | 1 | 0 | 0 | 1 | 0 | 0 | 0 | 0 | 0 |
| Microbryum davallianum | 0 | 1 | 0 | 1 | 1 | 0 | 0 | 0 | 0 | 0 |
| Microbryum starckeanum | 0 | 1 | 0 | 1 | 1 | 0 | 0 | 0 | 0 | 0 |
| Microcampylopus laevigatus | 1 | 1 | 0 | 0 | 1 | 0 | 0 | 0 | 0 | 0 |
| Microeurhynchium pumilum | 1 | 1 | 0 | 0 | 1 | 0 | 0 | 0 | 0 | 0 |
| Mitthenothamium leptoreptans* | 0 | 0 | 1 | 0 | 0 | 0 | 0 | 0 | 0 | 0 |
| Mnium hornum | 1 | 1 | 0 | 0 | 0 | 0 | 0 | 0 | 0 | 0 |
| Molendoa clavuligera | 0 | 0 | 0 | 1 | 0 | 0 | 0 | 0 | 0 | 0 |
| Myurium hochstetteri* | 1 | 1 | 0 | 0 | 1 | 0 | 0 | 0 | 0 | 0 |
| Myurium sp. | 1 | 0 | 0 | 0 | 0 | 0 | 0 | 0 | 0 | 0 |
| Neckera cephalonica* | 1 | 1 | 0 | 0 | 1 | 0 | 0 | 0 | 0 | 0 |
| Neckera menziesii | 0 | 0 | 0 | 0 | 1 | 0 | 0 | 0 | 0 | 0 |
| Neckera pennata | 0 | 0 | 0 | 0 | 1 | 0 | 0 | 0 | 0 | 0 |
| Neckera pumila | 0 | 1 | 0 | 0 | 1 | 0 | 0 | 0 | 0 | 0 |
| Neckeromnion lepineanum | 0 | 0 | 1 | 0 | 0 | 0 | 0 | 0 | 0 | 0 |
| Neckeropsis disticha | 0 | 0 | 1 | 0 | 0 | 0 | 0 | 0 | 0 | 0 |
| Nobregaea latinervis | 0 | 1 | 0 | 0 | 0 | 0 | 0 | 0 | 0 | 0 |
| Nogopterium gracile | 1 | 1 | 0 | 1 | 0 | 0 | 0 | 0 | 0 | 0 |
| Notoligotrichum tristaniense* | 0 | 0 | 0 | 0 | 0 | 0 | 0 | 0 | 0 | 1 |
| Notohypnum chrysogater | 0 | 0 | 0 | 0 | 0 | 0 | 0 | 0 | 0 | 1 |
| Octoblepharum albidum | 0 | 1 | 0 | 0 | 0 | 1 | 1 | 0 | 0 | 0 |
| Oedipodiella australis | 1 | 1 | 0 | 0 | 1 | 0 | 0 | 0 | 0 | 0 |
| Oncophorus fuegianus | 0 | 0 | 0 | 0 | 0 | 0 | 0 | 0 | 0 | 1 |
| Orthodontium gracile | 0 | 1 | 0 | 0 | 0 | 0 | 0 | 0 | 0 | 0 |
| Orthodontium pellucens | 0 | 1 | 0 | 0 | 1 | 0 | 0 | 0 | 0 | 0 |
| Orthostichella rigida | 0 | 0 | 1 | 0 | 0 | 0 | 0 | 0 | 0 | 0 |
| Orthostichella versicolor | 0 | 0 | 1 | 0 | 0 | 0 | 0 | 0 | 0 | 0 |
| Orthostichidium involutifolium subsp. involutifolium | 0 | 0 | 1 | 0 | 0 | 0 | 0 | 0 | 0 | 0 |
| Orthostichidium involutifolium subsp. thomeanum | 0 | 0 | 1 | 0 | 0 | 0 | 0 | 0 | 0 | 0 |
| Orthostichopsis subimbricata | 0 | 0 | 0 | 0 | 0 | 0 | 1 | 0 | 0 | 0 |
| Orthotrichum comosum | 0 | 1 | 0 | 0 | 1 | 0 | 0 | 0 | 0 | 0 |
| Orthotrichum cupulatum | 0 | 1 | 0 | 0 | 1 | 0 | 0 | 0 | 0 | 0 |
| Orthotrichum diaphanum | 1 | 1 | 0 | 1 | 1 | 0 | 0 | 0 | 0 | 0 |
| Orthotrichum handiense* | 0 | 0 | 0 | 0 | 1 | 0 | 0 | 0 | 0 | 0 |
| Orthotrichum pallens | 0 | 1 | 0 | 0 | 0 | 0 | 0 | 0 | 0 | 0 |
| Orthotrichum pumilum | 0 | 0 | 0 | 1 | 1 | 0 | 0 | 0 | 0 | 0 |
| Orthotrichum scanicum | 0 | 1 | 0 | 0 | 0 | 0 | 0 | 0 | 0 | 0 |
| Orthotrichum shevockii | 0 | 0 | 0 | 0 | 1 | 0 | 0 | 0 | 0 | 0 |
| Orthotrichum tenellum | 1 | 1 | 0 | 0 | 1 | 0 | 0 | 0 | 0 | 0 |
| Oxyrrhynchium hians | 1 | 1 | 0 | 0 | 1 | 0 | 0 | 0 | 1 | 0 |
| Oxyrrhynchium schleicheri | 0 | 1 | 0 | 0 | 1 | 0 | 0 | 0 | 0 | 0 |
| Oxyrrhynchium speciosum | 1 | 1 | 0 | 1 | 1 | 0 | 0 | 0 | 0 | 0 |
| Palamocladium leskeoides | 0 | 0 | 0 | 1 | 0 | 0 | 0 | 0 | 0 | 0 |
| Palustriella commutata | 0 | 1 | 0 | 0 | 0 | 0 | 0 | 0 | 0 | 0 |
| Palustriella falcata | 0 | 1 | 0 | 0 | 0 | 0 | 0 | 0 | 0 | 0 |
| Paraleucobryum longifolium | 0 | 1 | 0 | 0 | 0 | 0 | 0 | 0 | 0 | 0 |
| Pelekium atlanticum* | 0 | 1 | 0 | 0 | 1 | 0 | 0 | 0 | 0 | 0 |
| Pelekium involvens | 0 | 0 | 1 | 0 | 0 | 0 | 0 | 0 | 0 | 0 |
| Pelekium minutulum | 0 | 1 | 0 | 0 | 0 | 0 | 0 | 0 | 0 | 0 |
| Perssonia sanguinea* | 0 | 0 | 0 | 1 | 0 | 0 | 0 | 0 | 0 | 0 |
| Philonotis capillata* | 0 | 0 | 0 | 0 | 0 | 0 | 0 | 0 | 0 | 1 |
| Philonotis cernua | 0 | 0 | 0 | 0 | 0 | 0 | 1 | 0 | 0 | 0 |
| Philonotis dregeana | 0 | 0 | 0 | 0 | 0 | 0 | 0 | 1 | 0 | 0 |
| Philonotis fontana | 0 | 0 | 0 | 0 | 0 | 0 | 0 | 0 | 1 | 0 |
| Philonotis hastata | 0 | 0 | 0 | 0 | 0 | 1 | 0 | 1 | 0 | 0 |
| Philonotis helenica* | 0 | 0 | 0 | 0 | 0 | 0 | 0 | 0 | 1 | 0 |
| Philonotis penicillata* | 0 | 0 | 0 | 0 | 0 | 0 | 0 | 1 | 0 | 0 |
| Philonotis scabrifolia | 0 | 0 | 0 | 0 | 0 | 0 | 0 | 0 | 0 | 1 |
| Philonotis subolescens* | 0 | 0 | 0 | 0 | 0 | 0 | 0 | 1 | 0 | 0 |
| Philonotis tenuis | 0 | 0 | 0 | 0 | 0 | 0 | 0 | 0 | 0 | 1 |
| Philonotis vagans | 0 | 0 | 0 | 0 | 0 | 0 | 0 | 0 | 0 | 1 |
| Phyllogonium fulgens | 0 | 0 | 0 | 0 | 0 | 0 | 0 | 0 | 0 | 1 |
| Phyllogonium viscosum | 0 | 0 | 0 | 0 | 0 | 0 | 0 | 0 | 0 | 1 |
| Physcomitrium auberti | 0 | 0 | 0 | 0 | 0 | 0 | 0 | 0 | 0 | 1 |
| Physcomitrium flexifolium* | 0 | 0 | 0 | 0 | 0 | 0 | 0 | 1 | 1 | 0 |
| Physcomitrium pyriforme | 1 | 1 | 0 | 0 | 1 | 0 | 0 | 0 | 0 | 0 |
| Physcomitrium sp. | 0 | 0 | 0 | 0 | 0 | 0 | 0 | 0 | 1 | 0 |
| Pinnatella minuta | 0 | 0 | 1 | 0 | 0 | 0 | 0 | 0 | 0 | 0 |
| Pinnatidendron piniforme | 0 | 0 | 1 | 0 | 0 | 0 | 0 | 0 | 0 | 0 |
| Plagiomnium affine | 0 | 1 | 0 | 0 | 1 | 0 | 0 | 0 | 0 | 0 |
| Plagiomnium medium | 0 | 1 | 0 | 0 | 0 | 0 | 0 | 0 | 0 | 0 |
| Plagiomnium rhynchophorum | 0 | 0 | 1 | 0 | 0 | 0 | 0 | 0 | 0 | 0 |
| Plagiomnium rostratum | 1 | 1 | 0 | 0 | 1 | 0 | 0 | 0 | 0 | 0 |
| Plagiomnium undulatum | 1 | 1 | 0 | 0 | 1 | 0 | 0 | 0 | 0 | 0 |
| Plagiomnium undulatum var. madeirense* | 1 | 1 | 0 | 0 | 0 | 0 | 0 | 0 | 0 | 0 |
| Plagiothecium denticulatum | 0 | 1 | 0 | 0 | 0 | 0 | 0 | 0 | 0 | 0 |
| Plagiothecium nemorale | 1 | 1 | 0 | 0 | 1 | 0 | 0 | 0 | 0 | 0 |
| Plagiothecium suculentum | 1 | 1 | 0 | 0 | 0 | 0 | 0 | 0 | 0 | 0 |
| Plasteurhynchium meridionale | 1 | 1 | 0 | 1 | 1 | 0 | 0 | 0 | 0 | 0 |
| Platyneuron praealtum | 0 | 0 | 0 | 0 | 0 | 0 | 0 | 0 | 0 | 1 |
| Platygyriella densa | 0 | 0 | 0 | 1 | 0 | 0 | 0 | 0 | 0 | 0 |
| Plaubelia sprengelii | 0 | 0 | 0 | 0 | 0 | 0 | 1 | 0 | 0 | 0 |
| Pleuridium acuminatum | 1 | 1 | 0 | 0 | 1 | 0 | 0 | 1 | 1 | 0 |
| Pleuridium subulatum | 0 | 1 | 0 | 0 | 1 | 0 | 0 | 0 | 0 | 0 |
| Pleuridium sp. | 0 | 0 | 0 | 1 | 0 | 0 | 0 | 0 | 0 | 0 |
| Pleurochaete squarrosa | 1 | 1 | 0 | 1 | 1 | 0 | 0 | 0 | 0 | 0 |
| Pleuropus leskeoides | 0 | 0 | 0 | 0 | 0 | 0 | 0 | 0 | 0 | 1 |
| Pleurozium schreberi | 1 | 1 | 0 | 0 | 0 | 0 | 0 | 0 | 0 | 0 |
| Pogonatum aloides | 1 | 1 | 0 | 0 | 1 | 0 | 0 | 0 | 0 | 0 |
| Pogonatum gracilifolium | 0 | 0 | 1 | 0 | 1 | 0 | 0 | 0 | 0 | 0 |
| Pogonatum nanum | 1 | 1 | 0 | 0 | 1 | 0 | 0 | 0 | 0 | 0 |
| Pogonatum urnigerum | 1 | 1 | 0 | 0 | 1 | 0 | 0 | 0 | 0 | 0 |
| Pogonatum usambaricum | 0 | 0 | 1 | 0 | 0 | 0 | 0 | 0 | 0 | 0 |
| Pohlia annotina | 1 | 1 | 0 | 0 | 1 | 0 | 0 | 0 | 0 | 0 |
| Pohlia bulbifera | 1 | 0 | 0 | 0 | 0 | 0 | 0 | 0 | 0 | 0 |
| Pohlia cruda | 1 | 1 | 0 | 0 | 1 | 0 | 0 | 0 | 0 | 0 |
| Pohlia elongata | 0 | 1 | 0 | 0 | 1 | 0 | 0 | 0 | 0 | 1 |
| Pohlia excurrens* | 0 | 0 | 0 | 0 | 0 | 0 | 0 | 0 | 0 | 1 |
| Pohlia melanodon | 1 | 1 | 0 | 0 | 1 | 0 | 0 | 0 | 0 | 0 |
| Pohlia nutans | 1 | 1 | 0 | 0 | 0 | 0 | 0 | 0 | 0 | 1 |
| Pohlia proligera | 1 | 0 | 0 | 0 | 1 | 0 | 0 | 0 | 0 | 0 |
| Pohlia wahlenbergii | 0 | 0 | 0 | 0 | 1 | 0 | 0 | 0 | 0 | 1 |
| Polytrichadelphus magellanicus | 0 | 0 | 0 | 0 | 0 | 0 | 0 | 0 | 0 | 1 |
| Polytrichum commune | 1 | 1 | 0 | 0 | 1 | 0 | 0 | 0 | 0 | 0 |
| Polytrichun formosum | 1 | 1 | 0 | 0 | 1 | 0 | 0 | 0 | 0 | 0 |
| Polytrichum juniperinum | 1 | 1 | 0 | 0 | 1 | 0 | 0 | 0 | 0 | 1 |
| Polytrichum piliferum | 1 | 1 | 0 | 0 | 1 | 0 | 0 | 0 | 0 | 0 |
| Porotrichum atlanticum* | 0 | 0 | 0 | 0 | 0 | 0 | 0 | 0 | 1 | 0 |
| Porotrichum stipitatum | 0 | 0 | 0 | 0 | 1 | 0 | 0 | 0 | 0 | 0 |
| Pottiopsis caespitosa | 1 | 1 | 0 | 1 | 0 | 0 | 0 | 0 | 0 | 0 |
| Pseudephemerum nitidum | 1 | 1 | 0 | 0 | 0 | 0 | 0 | 0 | 0 | 0 |
| Pseudocrossidium crinitum | 0 | 0 | 0 | 0 | 0 | 0 | 0 | 0 | 1 | 0 |
| Pseudocrossidium hornschuchianum | 1 | 1 | 0 | 0 | 1 | 0 | 0 | 0 | 0 | 0 |
| Pseudocrossidium revolutum | 1 | 1 | 0 | 0 | 1 | 0 | 0 | 0 | 0 | 0 |
| Pseudohomalia webbiana* | 1 | 1 | 0 | 0 | 1 | 0 | 0 | 0 | 0 | 0 |
| Pseudoleskea pseudoattenuata | 0 | 0 | 0 | 1 | 0 | 0 | 0 | 0 | 0 | 0 |
| Pseudoleskeopsis bollei | 0 | 0 | 0 | 1 | 0 | 0 | 0 | 0 | 0 | 0 |
| Pseudorhynchostegiella duriaei | 1 | 1 | 0 | 0 | 1 | 0 | 0 | 0 | 0 | 0 |
| Pseudoscleropodium purum | 1 | 1 | 0 | 0 | 1 | 0 | 0 | 0 | 0 | 0 |
| Pseudotaxiphyllum elegans | 1 | 1 | 0 | 0 | 1 | 0 | 0 | 0 | 0 | 0 |
| Pseudotaxiphyllum laetevirens* | 1 | 1 | 0 | 0 | 0 | 0 | 0 | 0 | 0 | 0 |
| Psilopilum antarcticum | 0 | 0 | 0 | 0 | 0 | 0 | 0 | 0 | 0 | 1 |
| Psilopilum laxifolium* | 0 | 0 | 0 | 0 | 0 | 0 | 0 | 0 | 0 | 1 |
| Pterigynandrum filiforme | 0 | 1 | 0 | 1 | 1 | 0 | 0 | 0 | 0 | 0 |
| Pterygoneurum ovatum | 0 | 1 | 0 | 0 | 1 | 0 | 0 | 0 | 0 | 0 |
| Pterygoneurum subsessile | 0 | 0 | 0 | 0 | 1 | 0 | 0 | 0 | 0 | 0 |
| Ptychomitrium nigrescens* | 1 | 1 | 0 | 1 | 1 | 0 | 0 | 0 | 0 | 0 |
| Ptychomitrium polyphyllum | 1 | 1 | 0 | 0 | 1 | 0 | 0 | 0 | 0 | 0 |
| Ptychomnion densifolium | 0 | 0 | 0 | 0 | 0 | 0 | 0 | 0 | 0 | 1 |
| Ptychostomum bornholmense | 0 | 0 | 0 | 0 | 1 | 0 | 0 | 0 | 0 | 0 |
| Ptychostomum capillare | 1 | 1 | 0 | 1 | 1 | 0 | 0 | 0 | 0 | 0 |
| Ptychostomum celullare | 0 | 0 | 0 | 1 | 1 | 0 | 0 | 0 | 0 | 0 |
| Ptychostomum creberrimum | 1 | 0 | 0 | 0 | 0 | 0 | 0 | 0 | 0 | 0 |
| Ptychostomum donianum | 1 | 1 | 0 | 0 | 1 | 0 | 0 | 0 | 0 | 0 |
| Ptychostomum funkii | 0 | 0 | 0 | 0 | 1 | 0 | 0 | 0 | 0 | 0 |
| Ptychostomum imbricatulum | 1 | 1 | 0 | 0 | 1 | 0 | 0 | 0 | 0 | 0 |
| Ptychostomum kunzei | 1 | 0 | 0 | 0 | 1 | 0 | 0 | 0 | 0 | 0 |
| Ptychostomum moravicum | 0 | 0 | 0 | 0 | 1 | 0 | 0 | 0 | 0 | 0 |
| Ptychostomum pallens | 0 | 0 | 0 | 0 | 1 | 0 | 0 | 0 | 0 | 0 |
| Ptychostomum pallescens | 0 | 0 | 0 | 0 | 1 | 0 | 0 | 0 | 0 | 0 |
| Ptychostomum pseudotriquetrum | 1 | 1 | 0 | 0 | 1 | 0 | 0 | 0 | 0 | 0 |
| Ptychostomum pseudotriquetrum var. bimum | 0 | 1 | 0 | 0 | 0 | 0 | 0 | 0 | 0 | 0 |
| Ptychostomum rubens | 1 | 1 | 0 | 0 | 1 | 0 | 0 | 0 | 0 | 0 |
| Ptychostomum schleicheri | 1 | 0 | 0 | 0 | 0 | 0 | 0 | 0 | 0 | 0 |
| Ptychostomum torquescens | 1 | 1 | 0 | 1 | 1 | 0 | 0 | 0 | 0 | 0 |
| Pulvigera lyellii | 0 | 1 | 0 | 0 | 1 | 0 | 0 | 0 | 0 | 0 |
| Pylaisia polyantha | 0 | 0 | 0 | 0 | 1 | 0 | 0 | 0 | 0 | 0 |
| Pyrrhobryum spiniforme | 0 | 0 | 0 | 0 | 0 | 0 | 0 | 0 | 1 | 1 |
| Racomitrium aciculare | 1 | 1 | 0 | 0 | 1 | 0 | 0 | 0 | 0 | 0 |
| Racomitrium affine | 0 | 1 | 0 | 0 | 0 | 0 | 0 | 0 | 0 | 0 |
| Racomitrium aquaticum | 1 | 1 | 0 | 0 | 1 | 0 | 0 | 0 | 0 | 0 |
| Racomitrium crispulum | 0 | 0 | 0 | 0 | 0 | 0 | 0 | 0 | 0 | 1 |
| Racomitrium decurrens* | 0 | 0 | 0 | 0 | 0 | 0 | 0 | 0 | 0 | 1 |
| Racomitrium ellipticum | 0 | 0 | 0 | 0 | 1 | 0 | 0 | 0 | 0 | 0 |
| Racomitrium elongatum | 1 | 1 | 0 | 0 | 0 | 0 | 0 | 0 | 0 | 0 |
| Racomitrium ericoides | 1 | 0 | 0 | 0 | 0 | 0 | 0 | 0 | 0 | 0 |
| Racomitrium fasciculare | 1 | 1 | 0 | 0 | 0 | 0 | 0 | 0 | 0 | 0 |
| Racomitrium gracillimum* | 0 | 0 | 0 | 0 | 0 | 0 | 0 | 0 | 0 | 1 |
| Racomitrium heterostichum | 1 | 1 | 0 | 0 | 1 | 0 | 0 | 0 | 0 | 0 |
| Racomitrium laevigatum | 0 | 0 | 0 | 0 | 0 | 0 | 0 | 0 | 0 | 1 |
| Racomitrium lamprocarpum | 0 | 0 | 0 | 0 | 0 | 0 | 0 | 0 | 0 | 1 |
| Racomitrium lanuginosum | 1 | 1 | 0 | 0 | 1 | 0 | 0 | 0 | 0 | 1 |
| Racomitrium symphyodontum | 0 | 0 | 0 | 0 | 0 | 0 | 0 | 0 | 0 | 1 |
| Racopilum naumannii* | 0 | 0 | 0 | 0 | 0 | 0 | 0 | 1 | 0 | 0 |
| Racopilum orthocarpioides | 0 | 0 | 1 | 0 | 0 | 0 | 0 | 0 | 0 | 0 |
| Racopilum thomeanum | 0 | 0 | 1 | 0 | 0 | 0 | 0 | 0 | 0 | 0 |
| Rhabdoweisia fugax | 1 | 1 | 0 | 0 | 1 | 0 | 0 | 0 | 0 | 0 |
| Rhacocarpus purpuracens | 0 | 0 | 0 | 0 | 0 | 0 | 0 | 0 | 0 | 1 |
| Rhacopilopsis trinitensis | 0 | 0 | 1 | 0 | 0 | 0 | 0 | 0 | 0 | 0 |
| Rhamphidium purpuratum | 1 | 1 | 0 | 0 | 1 | 0 | 0 | 0 | 0 | 0 |
| Rhizofabronia persoonii | 0 | 0 | 1 | 0 | 0 | 0 | 0 | 0 | 0 | 0 |
| Rhizofabronia persoonii var. sphaerocarpa | 0 | 0 | 1 | 0 | 0 | 0 | 0 | 0 | 0 | 0 |
| Rhizomnium punctatum | 1 | 1 | 0 | 0 | 0 | 0 | 0 | 0 | 0 | 0 |
| Rhynchostegiella azorica* | 1 | 0 | 0 | 0 | 0 | 0 | 0 | 0 | 0 | 0 |
| Rhynchostegiella bourgaeana* | 0 | 0 | 0 | 0 | 1 | 0 | 0 | 0 | 0 | 0 |
| Rhynchostegiella pseudolitorea* | 0 | 0 | 0 | 0 | 1 | 0 | 0 | 0 | 0 | 0 |
| Rhynchostegiella teneriffae | 0 | 1 | 0 | 0 | 1 | 0 | 0 | 0 | 0 | 0 |
| Rhynchostegiella trichophylla* | 0 | 1 | 0 | 0 | 1 | 0 | 0 | 0 | 0 | 0 |
| Rhynchostegium confertum | 1 | 1 | 0 | 0 | 1 | 0 | 0 | 0 | 0 | 0 |
| Rhynchostegium hopfferi* | 0 | 0 | 1 | 0 | 0 | 0 | 0 | 0 | 0 | 0 |
| Rhynchostegium irriguum* | 0 | 0 | 0 | 0 | 0 | 0 | 0 | 0 | 0 | 1 |
| Rhynchostegium isopterygioides* | 0 | 0 | 0 | 0 | 0 | 0 | 0 | 0 | 0 | 1 |
| Rhynchostegium megapolitanum | 1 | 1 | 0 | 1 | 1 | 0 | 0 | 0 | 0 | 0 |
| Rhynchostegium murale | 0 | 1 | 0 | 0 | 1 | 0 | 0 | 0 | 0 | 0 |
| Rhynchostegium riparioides | 1 | 1 | 0 | 1 | 1 | 0 | 0 | 0 | 0 | 0 |
| Rhytidiadelphus loreus | 1 | 1 | 0 | 0 | 0 | 0 | 0 | 0 | 0 | 0 |
| Rhytidiadelphus squarrosus | 1 | 1 | 0 | 0 | 0 | 0 | 0 | 0 | 0 | 0 |
| Rhytidiadelphus subpinnatus | 1 | 0 | 0 | 0 | 0 | 0 | 0 | 0 | 0 | 0 |
| Sainthelenia athroclada* | 0 | 0 | 0 | 0 | 0 | 0 | 0 | 0 | 1 | 0 |
| Sanionia uncinata | 0 | 1 | 0 | 0 | 1 | 0 | 0 | 0 | 0 | 1 |
| Scabrellifolium elongatum | 0 | 0 | 1 | 0 | 0 | 0 | 0 | 0 | 0 | 0 |
| Scabrellifolium substriatum | 0 | 0 | 1 | 0 | 0 | 0 | 0 | 0 | 0 | 0 |
| Schistidium agassizii | 1 | 1 | 0 | 0 | 0 | 0 | 0 | 0 | 0 | 0 |
| Schistidium apocarpum | 1 | 1 | 0 | 0 | 1 | 0 | 0 | 0 | 0 | 0 |
| Schistidium confertum | 0 | 0 | 0 | 0 | 1 | 0 | 0 | 0 | 0 | 0 |
| Schistidium flaccidum | 0 | 1 | 0 | 0 | 1 | 0 | 0 | 0 | 0 | 0 |
| Schistidium frigidum | 0 | 0 | 0 | 0 | 1 | 0 | 0 | 0 | 0 | 0 |
| Schistidium rivulare | 0 | 1 | 0 | 0 | 0 | 0 | 0 | 0 | 0 | 0 |
| Schistidium strictum | 0 | 1 | 0 | 0 | 0 | 0 | 0 | 0 | 0 | 0 |
| Schizymenium pontevedrense* | 0 | 1 | 0 | 0 | 0 | 0 | 0 | 0 | 0 | 0 |
| Schlotheimia atlantica* | 0 | 0 | 0 | 0 | 0 | 0 | 0 | 0 | 0 | 1 |
| Schlotheimia jamesonii | 0 | 0 | 0 | 0 | 0 | 0 | 0 | 1 | 0 | 0 |
| Schlotheimia sp. | 0 | 0 | 1 | 0 | 0 | 0 | 0 | 0 | 0 | 0 |
| Sciuro-hypnum plumosum | 1 | 1 | 0 | 0 | 1 | 0 | 0 | 0 | 0 | 0 |
| Sciuro-hypnum populeum | 1 | 1 | 0 | 0 | 0 | 0 | 0 | 0 | 0 | 0 |
| Scleropodium touretii | 1 | 1 | 0 | 1 | 1 | 0 | 0 | 0 | 0 | 0 |
| Scopelophila cataractae | 0 | 0 | 0 | 0 | 0 | 0 | 0 | 1 | 0 | 0 |
| Scopelophila ligulata | 1 | 0 | 0 | 0 | 1 | 0 | 0 | 0 | 0 | 0 |
| Scorpiurium circinatum | 1 | 1 | 0 | 1 | 1 | 0 | 0 | 0 | 0 | 0 |
| Scorpiurium deflexifolium | 0 | 1 | 0 | 0 | 1 | 0 | 0 | 0 | 0 | 0 |
| Sematophyllum amblystegiocarpum* | 0 | 0 | 1 | 0 | 0 | 0 | 0 | 0 | 0 | 0 |
| Sematophyllum crassiusculum | 0 | 0 | 0 | 0 | 0 | 0 | 0 | 0 | 0 | 1 |
| Sematophyllum erythrocaulon* | 0 | 0 | 0 | 0 | 0 | 0 | 0 | 0 | 1 | 0 |
| Sematophyllum helenicum* | 0 | 0 | 0 | 0 | 0 | 0 | 0 | 0 | 1 | 0 |
| Sematophyllum substrumulosum | 1 | 1 | 0 | 0 | 1 | 0 | 0 | 0 | 0 | 0 |
| Sphagnum affine | 1 | 0 | 0 | 0 | 0 | 0 | 0 | 0 | 0 | 0 |
| Sphagnum auriculatum | 1 | 1 | 0 | 0 | 0 | 0 | 0 | 0 | 0 | 0 |
| Sphagnum capillifolium | 1 | 1 | 0 | 0 | 0 | 0 | 0 | 0 | 0 | 0 |
| Sphagnum centrale | 1 | 0 | 0 | 0 | 0 | 0 | 0 | 0 | 0 | 0 |
| Sphagnum compactum | 1 | 1 | 0 | 0 | 0 | 0 | 0 | 0 | 0 | 0 |
| Sphagnum cuspidatum | 1 | 0 | 0 | 0 | 0 | 0 | 0 | 0 | 0 | 0 |
| Sphagnum fimbriatum | 0 | 0 | 0 | 0 | 0 | 0 | 0 | 0 | 0 | 1 |
| Sphagnum girgensohnii | 1 | 0 | 0 | 0 | 0 | 0 | 0 | 0 | 0 | 0 |
| Sphagnum helenicum* | 0 | 0 | 0 | 0 | 0 | 0 | 0 | 0 | 1 | 0 |
| Sphagnum inundatum | 1 | 1 | 0 | 0 | 0 | 0 | 0 | 0 | 0 | 0 |
| Sphagnum magellanicum | 1 | 0 | 0 | 0 | 0 | 0 | 0 | 0 | 0 | 1 |
| Sphagnum nitidulum* | 1 | 0 | 0 | 0 | 0 | 0 | 0 | 0 | 0 | 0 |
| Sphagnum palustre | 1 | 0 | 0 | 0 | 0 | 0 | 0 | 1 | 0 | 0 |
| Sphagnum papillosum | 1 | 0 | 0 | 0 | 0 | 0 | 0 | 0 | 0 | 0 |
| Sphagnum recurvum | 1 | 0 | 0 | 0 | 0 | 0 | 0 | 0 | 0 | 1 |
| Sphagnum rubellum | 1 | 1 | 0 | 0 | 0 | 0 | 0 | 0 | 0 | 0 |
| Sphagnum squarrosum | 1 | 1 | 0 | 0 | 0 | 0 | 0 | 0 | 0 | 0 |
| Sphagnum subnitens | 1 | 1 | 0 | 0 | 0 | 0 | 0 | 0 | 0 | 0 |
| Sphagnum subsecundum | 1 | 0 | 0 | 0 | 0 | 0 | 0 | 0 | 0 | 0 |
| Sphagnum violascens | 0 | 0 | 0 | 0 | 0 | 0 | 0 | 0 | 0 | 1 |
| Splachnobryum obtusum | 0 | 1 | 1 | 0 | 0 | 0 | 1 | 1 | 0 | 0 |
| Streblotrichum convolutum | 1 | 1 | 0 | 1 | 1 | 0 | 0 | 0 | 0 | 0 |
| Syntrichia amphidiaceae | 0 | 0 | 0 | 1 | 0 | 0 | 0 | 0 | 0 | 0 |
| Syntrichia bogotensis | 0 | 1 | 0 | 0 | 0 | 0 | 0 | 0 | 0 | 0 |
| Syntrichia calcicola | 0 | 1 | 0 | 0 | 0 | 0 | 0 | 0 | 0 | 0 |
| Syntrichia fragilis | 0 | 1 | 0 | 1 | 1 | 0 | 0 | 0 | 0 | 0 |
| Syntrichia laevipila | 1 | 1 | 0 | 1 | 1 | 0 | 0 | 0 | 0 | 0 |
| Syntrichia latifolia | 0 | 1 | 0 | 0 | 0 | 0 | 0 | 0 | 0 | 0 |
| Syntrichia minor | 0 | 0 | 0 | 0 | 1 | 0 | 0 | 0 | 0 | 0 |
| Syntrichia montana | 0 | 1 | 0 | 0 | 1 | 0 | 0 | 0 | 0 | 0 |
| Syntrichia montana var. calva | 0 | 1 | 0 | 0 | 0 | 0 | 0 | 0 | 0 | 0 |
| Syntrichia norvegica | 0 | 1 | 0 | 0 | 0 | 0 | 0 | 0 | 0 | 0 |
| Syntrichia papillosa | 0 | 0 | 0 | 0 | 1 | 0 | 0 | 0 | 0 | 0 |
| Syntrichia princeps | 0 | 1 | 0 | 0 | 1 | 0 | 0 | 0 | 0 | 0 |
| Syntrichia ruralis | 1 | 1 | 0 | 0 | 1 | 0 | 0 | 0 | 0 | 0 |
| Syntrichia ruralis var. ruraliformis | 0 | 1 | 0 | 0 | 1 | 0 | 0 | 0 | 0 | 0 |
| Syntrichia virescens | 0 | 0 | 0 | 0 | 1 | 0 | 0 | 0 | 0 | 0 |
| Syrrhopodon armatus | 0 | 1 | 0 | 0 | 0 | 0 | 0 | 0 | 0 | 0 |
| Syrrhopodon gardneri | 0 | 1 | 0 | 0 | 0 | 0 | 0 | 0 | 0 | 0 |
| Syrrhopodon gaudichaudii | 0 | 1 | 0 | 0 | 0 | 0 | 0 | 0 | 0 | 1 |
| Syrrhopodon lamprocarpus | 0 | 1 | 0 | 0 | 0 | 0 | 0 | 0 | 0 | 0 |
| Syrrhopodon planifolius | 0 | 1 | 0 | 0 | 0 | 0 | 0 | 0 | 0 | 0 |
| Tayloria solitaria | 0 | 0 | 1 | 0 | 0 | 0 | 0 | 0 | 0 | 0 |
| Tetrastichium fontanum* | 1 | 1 | 0 | 0 | 1 | 0 | 0 | 0 | 0 | 0 |
| Tetrastichium virens* | 1 | 1 | 0 | 0 | 1 | 0 | 0 | 0 | 0 | 0 |
| Thamnobryum alopecurum var. alopecurum* | 1 | 1 | 0 | 0 | 1 | 0 | 0 | 0 | 0 | 0 |
| Thamnobryum corticola | 0 | 0 | 1 | 0 | 0 | 0 | 0 | 0 | 0 | 0 |
| Thamnobryum fernandesii* | 0 | 1 | 0 | 0 | 0 | 0 | 0 | 0 | 0 | 0 |
| Thamnobryum maderense* | 1 | 1 | 0 | 0 | 1 | 0 | 0 | 0 | 0 | 0 |
| Thamnobryum rudolphianum* | 1 | 0 | 0 | 0 | 0 | 0 | 0 | 0 | 0 | 0 |
| Thuidiopsis furfurosa | 0 | 0 | 0 | 0 | 0 | 0 | 0 | 0 | 0 | 1 |
| Thuidiopsis sparsa | 0 | 1 | 0 | 0 | 0 | 0 | 0 | 0 | 0 | 0 |
| Thuidium alvarezianum | 0 | 0 | 0 | 0 | 0 | 0 | 0 | 0 | 0 | 1 |
| Thuidium delicatulum | 1 | 0 | 0 | 0 | 0 | 0 | 0 | 0 | 0 | 0 |
| Thuidium tamariscinum | 1 | 1 | 0 | 0 | 0 | 0 | 0 | 0 | 0 | 0 |
| Timmiella anomala | 0 | 0 | 0 | 0 | 1 | 0 | 0 | 0 | 0 | 0 |
| Timmiella barbuloides | 1 | 1 | 0 | 1 | 1 | 0 | 0 | 0 | 0 | 0 |
| Timmiella cameruniae | 0 | 0 | 0 | 1 | 0 | 0 | 0 | 0 | 0 | 0 |
| Timmiella flexiseta | 0 | 0 | 0 | 0 | 1 | 0 | 0 | 0 | 0 | 0 |
| Tortella alpicola | 0 | 0 | 0 | 0 | 1 | 0 | 0 | 0 | 0 | 0 |
| Tortella fasciculata | 0 | 1 | 0 | 0 | 0 | 0 | 0 | 0 | 0 | 0 |
| Tortella flavovirens | 1 | 1 | 0 | 0 | 1 | 0 | 0 | 0 | 0 | 0 |
| Tortella fragilis | 1 | 0 | 0 | 0 | 0 | 0 | 0 | 0 | 0 | 0 |
| Tortella humilis | 0 | 1 | 0 | 0 | 0 | 0 | 0 | 0 | 0 | 0 |
| Tortella inflexa | 1 | 0 | 0 | 0 | 1 | 0 | 0 | 0 | 0 | 0 |
| Tortella limbata* | 0 | 1 | 0 | 0 | 1 | 0 | 0 | 0 | 0 | 0 |
| Tortella nitida | 1 | 1 | 0 | 0 | 1 | 0 | 0 | 0 | 0 | 0 |
| Tortella squarrosa | 1 | 1 | 0 | 1 | 1 | 0 | 0 | 0 | 0 | 0 |
| Tortella tortuosa | 1 | 1 | 0 | 0 | 1 | 0 | 0 | 0 | 0 | 0 |
| Tortula acaulon | 0 | 1 | 0 | 0 | 1 | 0 | 0 | 0 | 0 | 0 |
| Tortula ampliretis* | 0 | 0 | 0 | 0 | 1 | 0 | 0 | 0 | 0 | 0 |
| Tortula atrovirens | 1 | 1 | 0 | 1 | 1 | 0 | 0 | 0 | 1 | 0 |
| Tortula bogosica | 1 | 0 | 0 | 1 | 1 | 0 | 0 | 0 | 0 | 0 |
| Tortula bolanderi | 0 | 1 | 0 | 1 | 1 | 0 | 0 | 0 | 0 | 0 |
| Tortula brevissima | 0 | 0 | 0 | 0 | 1 | 0 | 0 | 0 | 0 | 0 |
| Tortula canescens | 0 | 0 | 0 | 0 | 1 | 0 | 0 | 0 | 0 | 0 |
| Tortula cuneifolia | 0 | 0 | 0 | 0 | 1 | 0 | 0 | 0 | 0 | 0 |
| Tortula cuspidatissima* | 0 | 0 | 0 | 0 | 0 | 0 | 0 | 1 | 0 | 0 |
| Tortula israelis | 0 | 0 | 0 | 0 | 1 | 0 | 0 | 0 | 0 | 0 |
| Tortula lindbergii | 0 | 1 | 0 | 0 | 0 | 0 | 0 | 0 | 0 | 0 |
| Tortula marginata | 1 | 1 | 0 | 1 | 1 | 0 | 0 | 0 | 0 | 0 |
| Tortula marginata subsp. limbata | 0 | 0 | 0 | 1 | 0 | 0 | 0 | 0 | 0 | 0 |
| Tortula muralis var. aestiva | 0 | 1 | 0 | 0 | 0 | 0 | 0 | 0 | 0 | 0 |
| Tortula muralis var. muralis | 1 | 1 | 0 | 1 | 1 | 0 | 0 | 0 | 1 | 0 |
| Tortula pallida | 0 | 1 | 0 | 0 | 1 | 0 | 0 | 0 | 0 | 0 |
| Tortula protobryoides | 0 | 0 | 0 | 0 | 1 | 0 | 0 | 0 | 0 | 0 |
| Tortula revolvens | 1 | 0 | 0 | 1 | 1 | 0 | 0 | 0 | 0 | 0 |
| Tortula solmsii | 1 | 1 | 0 | 1 | 1 | 0 | 0 | 0 | 0 | 0 |
| Tortula subulata | 0 | 1 | 0 | 0 | 1 | 0 | 0 | 0 | 0 | 0 |
| Tortula truncata | 1 | 1 | 0 | 0 | 1 | 0 | 0 | 0 | 0 | 0 |
| Tortula vahliana | 1 | 0 | 0 | 1 | 1 | 0 | 0 | 0 | 0 | 0 |
| Tortula viridifolia | 0 | 0 | 0 | 0 | 1 | 0 | 0 | 0 | 0 | 0 |
| Tortula sp. | 0 | 0 | 0 | 0 | 0 | 0 | 0 | 1 | 0 | 0 |
| Trachypodopsis serrulata | 0 | 0 | 1 | 0 | 0 | 0 | 0 | 0 | 0 | 0 |
| Trachypus bicolor var. viridulus | 0 | 0 | 1 | 0 | 0 | 0 | 0 | 0 | 0 | 0 |
| Trematodon divaricatus | 0 | 0 | 1 | 0 | 0 | 0 | 0 | 0 | 0 | 0 |
| Trematodon intermixtus | 0 | 0 | 0 | 0 | 0 | 0 | 0 | 0 | 0 | 1 |
| Trematodon longicollis | 0 | 0 | 1 | 0 | 0 | 0 | 0 | 0 | 0 | 0 |
| Trematodon perssoniorum* | 1 | 0 | 0 | 0 | 0 | 0 | 0 | 0 | 0 | 0 |
| Trichodon cylindricus | 0 | 0 | 0 | 0 | 1 | 0 | 0 | 0 | 0 | 0 |
| Trichosteleum dicranelloides | 0 | 0 | 1 | 0 | 0 | 0 | 0 | 0 | 0 | 0 |
| Trichostomum aequatoriale | 0 | 0 | 0 | 0 | 0 | 0 | 0 | 0 | 0 | 1 |
| Trichostomum brachydontium | 1 | 1 | 0 | 1 | 1 | 0 | 0 | 1 | 1 | 0 |
| Trichostomum crispulum | 1 | 1 | 0 | 1 | 1 | 0 | 0 | 1 | 1 | 0 |
| Trismegistia rigidicaulis | 0 | 0 | 1 | 0 | 0 | 0 | 0 | 0 | 0 | 0 |
| Ulota crispa | 1 | 1 | 0 | 0 | 1 | 0 | 0 | 0 | 0 | 0 |
| Ulota hutchinsiae | 0 | 0 | 0 | 0 | 1 | 0 | 0 | 0 | 0 | 0 |
| Venturiella glaziovii | 0 | 0 | 0 | 1 | 0 | 0 | 0 | 0 | 0 | 0 |
| Venturiella perrottetii | 0 | 0 | 0 | 1 | 0 | 0 | 0 | 0 | 0 | 0 |
| Vesicularia glaucula | 0 | 0 | 1 | 0 | 0 | 0 | 0 | 0 | 0 | 0 |
| Vesicularia scaturigina | 0 | 0 | 1 | 0 | 0 | 0 | 0 | 0 | 0 | 0 |
| Vesicularia strephomischos | 0 | 0 | 1 | 0 | 0 | 0 | 0 | 0 | 0 | 0 |
| Warnstorfia fluitans | 0 | 1 | 0 | 0 | 0 | 0 | 0 | 0 | 0 | 0 |
| Weissia angustifolia | 0 | 1 | 0 | 0 | 0 | 0 | 0 | 0 | 0 | 0 |
| Weissia brachycarpa | 1 | 0 | 0 | 1 | 0 | 0 | 0 | 0 | 0 | 0 |
| Weissia condensa | 1 | 1 | 0 | 1 | 1 | 0 | 0 | 0 | 0 | 0 |
| Weissia controversa | 1 | 1 | 0 | 1 | 1 | 0 | 0 | 0 | 1 | 0 |
| Weissia longifolia | 0 | 1 | 0 | 0 | 1 | 0 | 0 | 0 | 0 | 0 |
| Weissia squarrosa | 0 | 0 | 0 | 0 | 1 | 0 | 0 | 0 | 0 | 0 |
| Wijkia trichocoleoides* | 0 | 0 | 1 | 0 | 0 | 0 | 0 | 0 | 0 | 0 |
| Zygodon conoideus | 1 | 1 | 0 | 1 | 1 | 0 | 0 | 0 | 0 | 0 |
| Zygodon insularum* | 0 | 0 | 0 | 0 | 0 | 0 | 0 | 0 | 0 | 1 |
| Zygodon rupestris | 1 | 1 | 0 | 0 | 1 | 0 | 0 | 0 | 0 | 0 |
| Zygodon viridissimus | 1 | 1 | 0 | 0 | 1 | 0 | 0 | 0 | 0 | 0 |
