# Supplementary material for: Atlantic oceanic islands and archipelagos: Physical structures, plant diversity, and affinities of the bryofloras
Source: Biodivers Data J. 2025 Feb 28;13:e141577. doi: 10.3897/BDJ.13.e141577 (PMC11889432; doi:10.3897/BDJ.13.e141577)
Supplement: Supplementary material 3 — Table S3. Phytogeographical patterns of the bryofloras in the North Atlantic Ocean [file bdj-13-e141577-s003.docx]

**Supplemental Data**

**Table S3.** Phytogeographical patterns of the bryofloras of the five **North Atlantic Ocean** islands and archipelagos.

| Patterns | | Liverworts | Mosses | Total |
| --- | --- | --- | --- | --- |
| Worldwide | 21 | | 86 | 107 |
| Africa | 99 | | 83 | 182 |
| Endemic | 38 | | 69 | 107 |
| Europe | 0 | | 40 | 40 |
| N America, Europe and Asia | 26 | | 20 | 46 |
| Europe, Macaronesia, Africa | 7 | | 1 | 8 |
| Europe, Macaronesia, Asia | 8 | | 8 | 16 |
| Europe and Macaronesia | 5 | | 9 | 14 |
| South America and Africa | 5 | | -- | 5 |
| Others | 215 | | 382 | 597 |
| Total | 424 | | 698 | 1122** |

** This number is different because there are taxa identified only at genera level
