# Supplementary material for: Atlantic oceanic islands and archipelagos: Physical structures, plant diversity, and affinities of the bryofloras
Source: Biodivers Data J. 2025 Feb 28;13:e141577. doi: 10.3897/BDJ.13.e141577 (PMC11889432; doi:10.3897/BDJ.13.e141577)
Supplement: Supplementary material 4 — Table S4. Phytogeographical patterns of the bryofloras in the South Atlantic Ocean [file bdj-13-e141577-s004.docx]

**Supplemental Data**

**Table S4.** Phytogeographical patterns of the bryofloras of the five **South Atlantic Ocean** islands and archipelagos (Trindade, Fernando de Noronha, Ascension, St Helena, and Tristan da Cunha).

| Patterns | Liverworts | Mosses | Total |
| --- | --- | --- | --- |
| Worldwide | 15 | 35 | 50 |
| Pantropical | 10 | 10 | 20 |
| Tropical America | 11 | 10 | 21 |
| Africa | 6 | 13 | 19 |
| South America | 75 | 16 | 91 |
| Endemic | 36 | 55 | 91 |
| Others | 86 | 85 | 171 |
| Total | 239 | 224 | 463** |

** This number is different because there are taxa identified only at genera level
